# Psychometric properties and score distributions of the Clinical Outcomes in Routine Evaluation measures within a non-help-seeking population from Spain

**DOI:** 10.1186/s41155-025-00341-6

**Published:** 2025-09-02

**Authors:** Clara Paz, Luis Ángel Saúl, Pedro Ramírez Lafuente, Chris Evans

**Affiliations:** 1 Grupo de Investigación Bienestar, Salud y Sociedad, Escuela de Psicología y Educación, Universidad de Las Américas, Quito, Ecuador; 2 Facultad de Psicología, Universidad Nacional de Educación a Distancia (UNED), Madrid, Spain; 3 Investigador en Formación, Escuela Internacional de Doctorado, Programa de Doctorado en Psicología, Universidad Nacional de Educación a Distancia (UNED), Madrid, Spain

**Keywords:** Psychological distress, Referential data, CORE system, Spain, Psychometric properties

## Abstract

**Background:**

The Clinical Outcomes in Routine Evaluation (CORE) system is widely used to assess psychological well-being and clinical symptoms across various settings, but most studies on its psychometric properties have focused on clinical populations and the 34-item version, leaving a gap in understanding the performance of shorter versions and its applicability in non-help-seeking samples.

**Objective:**

This study investigates the acceptability, reliability, and score distributions of various forms within the CORE system among a non-help-seeking Spanish population.

**Methods:**

Data from 1667 participants were analyzed, with a mean age of 37.16 years and a predominance of women (59.1%). The majority had higher education (53.1%), and over half were employed at the time of the study.

**Results:**

Acceptability was high, with low item omission rates (<0.1%) across all forms during both initial and retest assessments. Internal consistency was strong, with Cronbach's alpha and McDonald's Omega exceeding 0.80 for all forms. Test-retest reliability showed correlations above 0.59 for all scores, with no significant differences between assessment intervals. Score distributions were compared by gender, age, and education, revealing significant differences between gender and education but not for age.

**Conclusion:**

The study provides key reference data for the CORE system in Spain, supporting outcome comparisons in non-help-seeking samples. Despite an overrepresentation of highly educated individuals, it offers crucial insights into its psychometric properties and score distributions. The findings highlight potential applications of these distributions and underscore the need for further research into the psychometric performance of individual CORE forms.

**Supplementary Information:**

The online version contains supplementary material available at 10.1186/s41155-025-00341-6.

## Introduction

The Clinical Outcomes in Routine Evaluation (CORE) system comprises a set of measures designed to assess psychological distress (Barkham et al., 1998). These measures support routine outcome monitoring of change and outcomes in mental health interventions (Evans et al., 2002). The CORE-OM, where OM stands for outcome measure, is the central measure of this system and comprises 34 items that measure four domains of psychological distress: well-being (4 items), problems/symptoms (12 items), functioning (12 items), and risk (6 items).

Several short forms have been derived from the CORE-OM, each created by selecting specific sets of items from the original 34-item measure to serve different purposes. One purpose was to reduce the burden of completion of the measure when offered on session by session. In this regard, two parallel forms of 18 items were created at the launch of the CORE system in 1998, called Short Form A (SFA) and Short Form B (SFB). These forms were created with the intention being used alternately during therapies to minimize memory effects. Later, the CORE-10 (Barkham et al., 2013), an even shorter form with only 10 items, was generated to provide a simpler repeat-use short form. The other reason for the creation of another short form was to have a measure suitable for the general population: GP-CORE (Evans et al., 2005). This measure has only 14 items omitting risk domain items and other high-tuned items of the CORE-OM. By selecting and adapting subsets of items from the full CORE-OM, these short forms maintain the overall structure and validity of the original measure while offering flexibility for different contexts and populations.

These measures were selected for the present study due to their well-established role in routine outcome monitoring worldwide and their flexibility to be used across clinical and non-clinical populations. These measures are copyleft, meaning they can be used without charges if they are not modified. The CORE-OM, and therefore all items included in the short forms, have been translated into more than 30 languages, including Spanish. By the end of 2021, 562 peer-reviewed papers using CORE measures had been published in English or Spanish, including 71 reporting data from non-clinical samples. The Spanish translation was conducted in Spain, and to date, only one psychometric study has been conducted in the country (Trujillo et al., 2016). That study analyzed responses to the CORE-OM from 192 participants in a clinical sample and 452 participants in a non-clinical sample, which included 127 community volunteers and 325 university students. In total, 78 participants from the non-clinical sample participated in a retest that occurred 15 to 30 days after the first administration. Although this study provided valuable information about the psychometric properties of the CORE-OM, there is still a lack of information about these properties for the short forms derived from the CORE-OM, limiting their use in Spanish.

With this background, the present study aims to address the following research question: What are the acceptability, reliability, and score distributions of the CORE-OM and its derived short forms (CORE-SFA, CORE-SFB, CORE-10, and GP-CORE) in a larger sample of the non-help-seeking Spanish population? We examined all embedded versions of the CORE system, as they span a range of applications—from clinical assessment (CORE-OM and its derivatives) to evaluations in the general population (GP-CORE). These measures are particularly relevant as they not only capture the multidimensional nature of psychological distress but also address the need for robust, Spanish-language tools for outcome monitoring and clinical assessment across diverse settings.

Alongside evaluating psychometric properties, we aim to establish a reference dataset specific to the Spanish population for the various forms of the CORE system. This reference dataset includes quantiles, which serve as a valuable resource for contextualizing individual scores in relation to the general non-help-seeking population. In psychotherapy research and clinical practice, the “Clinical Significant Change” (CSC) methodology introduced by Jacobson and Truax in 1991 is used with a binary classification that categorizes individuals into either the help-seeking (clinical) or non-help-seeking (general) distribution. Routine use of the CSC methods gains considerably when large non-help-seeking datasets contribute their means and SDs to the calculation of the CSC cutting point. However, by employing quantiles, clinicians and researchers gain the ability to not only compare scores above or below the CSC threshold but also to assess their placement within a broader distribution, allowing for a more comprehensive and nuanced analysis.

## Methods

### Participants

This study utilized a convenience sampling method. Undergraduate psychology students at the Universidad Nacional de Educación a Distancia (UNED) across Spain were tasked with recruiting participants from their personal networks as part of a class project designed to enhance their understanding of the psychological assessment process. They were encouraged to identify and invite two to three individuals who met the study’s inclusion criteria. These criteria required participants to be adults (18 years or older), residing in Spain, without psychological diagnosis and not undergoing psychological treatment.

Students were provided with clear instructions on the recruitment process to ensure ethical practices. This included informing potential participants about the voluntary nature of the study, explaining the study’s purpose, and emphasizing that their responses would remain anonymous and confidential. Participants were also required to provide informed consent before taking part in the research. This approach facilitated the collection of data from a non-help-seeking population across various regions of the country.

### Measures

CORE System Measures: the CORE-OM items are scored on a five-point Likert scale from 0 (“not at all”) to 4 (“most or all of the time”), with higher scores indicating higher levels of psychological distress. The CORE-OM was translated into Spanish in Spain (Trujillo et al., 2016), and it has demonstrated good internal consistency for the total score (α = 0.92, 95% CI [0.90, 0.94]) and for the non-risk items score (α = 0.93, 95% CI [0.91, 0.95]) when used with community samples, similar to the results found for the original version in the UK ( Evans et al., 2002), total score α = 0.94, 95% CI [0.93, 0.93], non-risk items α = 0.94, 95% CI [0.94, 0.95].

The embedded measures within the CORE-OM include the CORE-SFA (18 items), CORE-SFB (18 items), GP-CORE (14 items) and CORE-10 (10 items). Table 1 presents the mapping of the CORE-OM items included in each of the shorter forms. The psychometric properties of the CORE-10 have been explored in the UK by Barkham et al. (2013) with a clinical sample, revealing good internal consistency (α = 0.90, 95% CI [0.86, 0.92]), and in Ecuador by Valdiviezo-Oña et al. (2024) with a help-seeking sample, showing similar results (α = 0.81, 95% CI [0.78, 0.84]).

**Table 1:** Mapping of the CORE-OM items included in each of the shorter forms

	Included Items by Form				
Items	CORE-OM	CORE-SFA	CORE-SFB	CORE-10	CORE-GP
I have felt terribly alone and isolated	Included	–	Included	–	–
I have felt tense, anxious or nervous	Included	Included	–	Included	Included
I have felt I have someone to turn to for support when needed	Included	–	Included	Included	Included
I have felt O.K. about myself	Included	Included	Included	–	Included
I have felt totally lacking in energy and enthusiasm	Included	–	Included	–	–
I have been physically violent to others	Included	Included	–	–	–
I have felt able to cope when things go wrong	Included	Included	–	Included	Included
I have been troubled by aches, pains or other physical problems	Included	–	Included	–	Included
I have thought of hurting myself	Included	–	–	–	–
Talking to people has felt too much for me	Included	-	Included	Included	–
Tension and anxiety have prevented me doing important things	Included	–	Included	–	–
I have been happy with the things I have done	Included	–	Included	–	Included
I have been disturbed by unwanted thoughts and feelings	Included	–	Included	–	–
I have felt like crying	Included	Included	Included	–	–
I have felt panic or terror	Included	Included	–	Included	–
I made plans to end my life	Included	–	Included	Included	–
I have felt overwhelmed by my problems	Included	Included	Included	–	–
I have difficulty getting to sleep or staying asleep	Included	–	Included	Included	Included
I have felt warmth and affection for someone	Included	Included	–	–	Included
My problems have been impossible to put to one side	Included	Included	–	–	–
I have been able to do most things I needed to	Included	–	Included	–	Included
I have threatened or intimidated another person	Included	–	Included	–	–
I have felt despairing or hopeless	Included	Included	–	Included	–
I have thought it would be better if I were dead	Included	–	–	–	–
I have felt criticised by other people	Included	Included	–	–	Included
I have thought I have no friends	Included	–	Included	–	–
I have felt unhappy	Included	Included	–	Included	Included
Unwanted images or memories have been distressing me	Included	Included	–	Included	–
I have been irritable when with other people	Included	Included	–	–	Included
I have thought I am to blame for my problems and difficulties	Included	-	Included	–	–
I have felt optimistic about my future	Included	Included	Included	–	Included
I have achieved the things I wanted to	Included	Included	–	–	Included
I have felt humiliated or shamed by other people	Included	Included	–	–	–
I have hurt myself physically or taken dangerous risks with my health	Included	Included	–	–	–
Number of Items	34	18	18	10	14

### Procedures

Participants were invited to join the study as described above. Those who expressed interest in participating were informed about the study’s objectives, and a consent form was provided to them. Participants who agreed to take part signed a written consent and confidentiality form.

All participants then provided sociodemographic information, including age, gender, education, marital status, employment status, nationality, and province of residence in Spain. Afterward, they completed several questionnaires, including the CORE-OM. The other questionnaires administered were the Symptom Checklist-90-Revised, NEO Five-Factor Inventory, Inventory of Stressful Life Events, Childhood Abuse Assessment, Social Vulnerability Resilience Scale, Zung Self-Rating Depression Scale, Past Adverse Situations, Current Adverse Situations, Cognitive Emotional Dysfunction Test, Relationship Questionnaire, and the Repertory Grid: Repertory Grid Technique (commonly used in Personal Construct Theory). In the 2010–2011 cohort, participants were contacted again after 6 months to complete the questionnaires once more. In the 2012–2013 cohort, participants were contacted for the same purpose after 1 week.

### Analyses

The analyses followed the Problem, Plan, Data, Analyses, Conclusions and Communication (PPDAC) framework (MacKay & Oldford, 2000; Spiegelhalter, 2020). The identified Problem was the lack of information about the reliability and distribution of the scores for the various forms of the CORE system embedded with the CORE-OM. The plan was to analyze responses to the CORE-OM (Data) from a community sample collected between 2010 and 2018.

The analyses focused on four objectives. First, to describe the characteristics of the sample, we reported the mean, standard deviation, median, and confidence intervals for age (the only continuous variable), while frequencies were reported for other sociodemographic variables, with all these variables reported by tranche. Second, to assess the measure's acceptability, the omission rate was calculated for each item and each form. Third, to evaluate the reliability of the CORE-OM scores and the embedded short forms of the CORE system, we analyzed internal consistency and test–retest stability. Internal consistency was assessed using Cronbach’s alpha (Cronbach, 1951) and McDonald’s Omega (ω) coefficient for one-factor models (unidimensional measures with continuous items; Flora, 2020) for each form.

Test–retest stability was evaluated by examining Pearson correlation coefficients between assessment occasions and calculating the mean difference and 95% CI between those occasions for each form. For mean change, we reported both Cohen’s d1 and Cohen’s dz to allow comparisons with other studies that may have reported one but not the other due to a lack of consensus in the literature. Cohen’s d1 is calculated as the mean change divided by the standard deviation (SD) of the baseline values, while dz is the mean change divided by the SD of the change values. In test–retest studies, where a strong positive correlation between first and second scores is typical, dz tends to be larger than d1.

Fourth, we examined the mean differences between scores based on age, gender, and level of education. Based on those results, we reported the descriptive information (mean, Confidence Intervals [CI], minimum, maximum, and standard deviation) of the scores of the different forms of the CORE-OM for each category of the mentioned variables. Finally, we provided the distributions of the scores for each form to be used as reference data.

All the analyses were conducted using R (R Core Team, 2021) in RStudio (Posit team, 2022). Bootstrapped CI, shown as .xx[.xx,.xx], were calculated for Cronbach’s alpha coefficient, Pearson’s correlation, mean differences, and percentiles using the CECPfuns package (Evans, 2021). This package was also used to generate percentile distribution plots, while the other plots were created using ggplot2 (Wickham, 2016).

## Results

### Characteristics of the sample

In total, we analyzed the data of 1667 participants. The mean age was 37.16 (SD = 11.95) [36.60, 37.79], with ages ranging from 18 to 74 years. However, age information was missing for 3 participants. Regarding gender, 983 (59.1%) were women, 681(40.9%) were men and gender information was missing for 3 participants. More than half of the participants had higher education (*n* = 877, 53.1%), 39% (*n* = 645) had completed high school education, 77.7% (*n* = 128) had elementary education, 0.1% (*n* = 2) indicated that they had no formal education. Information about education was missing for 15 participants. In addition, more than half of the participants (*n* = 906, 55.3%) were employed at the time of completing the questionnaires, 21.6% (*n* = 353) were studying, 15.1% were unemployed, 6% (*n* = 98) were engaged in housework and 1.5% (*n* = 25) were retired. Employment status information was missing for 29 participants. In terms of marital status, 854 participants (51.7%) were single, 629 (38.1%) were married, 154 (9.3%) were separated or divorced, and 15 (0.9%) were widowed. Information about marital status was missing for 15 participants. The majority of the sample were Spanish nationals (*n* = 1607, 97%), and a quarter of the sample was living in the province of Madrid (*n* = 403, 25.2%) at the time of questionnaire completion. A comprehensive description of these sociodemographic characteristics by tranche is presented in Supplementary material.

When comparing our sample with the characteristics of the general population in Spain (Instituto Nacional de Estadística, 2022), we observed some differences. First, in terms of gender, our sample presents a greater proportion of women compared to the Spanish population (51%). Second, regarding age, the mean age of the Spanish population is 44.07 years, which is higher than the mean age in our sample. Finally, in terms of education, among the Spanish population aged between 24 to 64 years, 40% have higher education, whereas our sample includes a greater proportion of individuals with higher education.

### Acceptability

A total of 101 participants completed the retest questionnaire after 1 week in the 2012–2013 tranche, and 65 participants did so after 6 months in the 2010–2011 tranche. Table 2 shows the number of participants who completed all items and those who completed the minimum number of required items, allowing for a total score to be prorated based on the items they completed. Due to the sampling process used, we do not know the non-response rate for the entire questionnaire; however, we do have the non-completion rate for the CORE-OM among those who participated in the study as a whole.

**Table 2:** Number of participants that completed all the items and the minimum for proration by occasion (*n* = 1667)

Form	Number of items	T1^a^ *n*(%)	T2^b^ *n*(%)	Minimum for proration	T1 *n*(%)	T2 *n*(%)
CORE-OM	34	1,657(99.4)	165(99.4%)	31	1666(99.9%)	166(100%)
CORE-OM-NR	28	1,661(99.6)	165(99.4%)	26	1666(99.9%)	166(100%)
CORE-SFA	18	1,661(99.6)	165(99.4%)	16	1666(99.9%)	166(100%)
CORE-SFB	18	1,661(99.6)	165(99.4%)	16	1666(99.9%)	166(100%)
GP-CORE	14	1,663(99.8)	165(99.4%)	13	1666(99.9%)	166(100%)
CORE-10	10	1,663(99.8)	165(99.4%)	9	1666(99.9%)	166(100%)

^a^*T1* = initial assessment

^b^T2 = retest

**Fig. 1:**
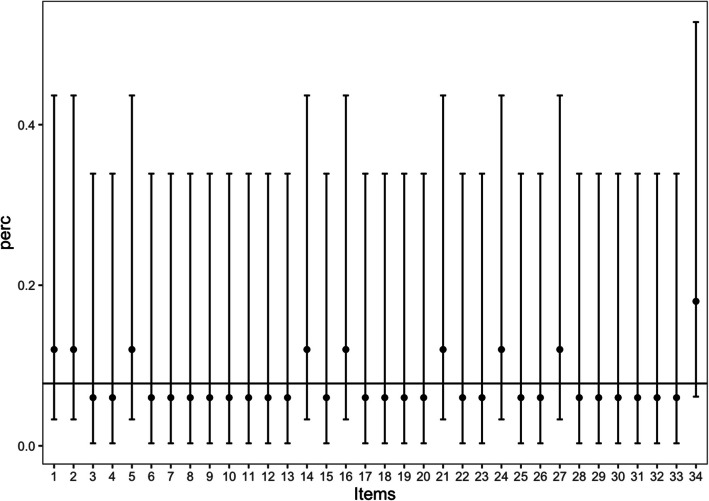
Omission rates and 95% confidence intervals for each item at initial assessment (T1). We detected effects for items 6, 9, 16, 22, 24, and 34, which received a high number of responses equal to 0. All of these items belong to the risk domain

For the CORE-OM, the item omission rate was very low during the initial assessment (T1), with a total omission rate of 0.07%. During the retest (T2), this rate further decreased to 0.02%. Figure 1 presents the omission rates with their 95% confidence intervals for each item at T1, with item 34 being the most frequently omitted, followed by items 1, 2, 5, 14, 16, 21, and 27. For the non-risk score, the omission rate was 0.06% at T1 and 0.02% at T2. For the CORE-SFA, the omission rate was 0.08% at T1 and 0.03% at T2. For CORE-SFB, the parallel form, the rate was 0.08% at T1 and 0.03% at T2. For the GP-CORE, the omission rate was 0.07% at T1 and 0.04% at T2. For the CORE-10, the rate was 0.08% at T1, with no omitted responses detected at T2. These findings highlight the high acceptability and completion rate of responses across all forms.

### Reliability

#### Internal consistency

At T1, the internal consistency, as measured by Cronbach’s alpha and McDonald’s omega, was above 0.80 for all the forms. As expected, forms with a greater number of items demonstrated greater consistency coefficients, as described in Table 3.

**Table 3:** Observed Cronbach’s alpha, bootstrapped 95% confidence intervals, and observed McDonald’s omega for each form by assessment occasion

Score	T1^b^ (*n* between 1657 and 1,667)		T2^c^ (*n* between 165 and 166)	
	Cronbach’s alpha [95% CI]^a^	McDonald’s omega	Cronbach’s alpha [95% CI]^a^	McDonald’s omega
CORE-OM	0.93 [0.93,0.94]	0.94	0.94 [0.92, 0.95]	0.95
CORE-OM-NR	0.93 [0.93,0.94]	0.94	0.94 [0.92, 0.95]	0.95
CORE-SFA	0.90 [0.89, 0.90]	0.91	0.91 [0.88, 0.92]	0.92
CORE-SFB	0.88 [0.87, 0.89]	0.90	0.88 [0.85, 0.91]	0.91
CORE-GP	0.85[0.84, 0.86]	0.88	0.85 [0.82, 0.88]	0.89
CORE-10	0.81 [0.79, 0.83]	0.84	0.83 [0.76, 0.87]	0.87

^a^Bootstrap confidence interval used the percentile method with 1000 bootstrap replications

^b^T1 = initial assessment

^c^T2 = retest

Reliability did not show significant differences among genders, age groups, education, or tranches. Detailed information about reliability concerning these variables is included in the supplementary material.

**Fig. 2:**
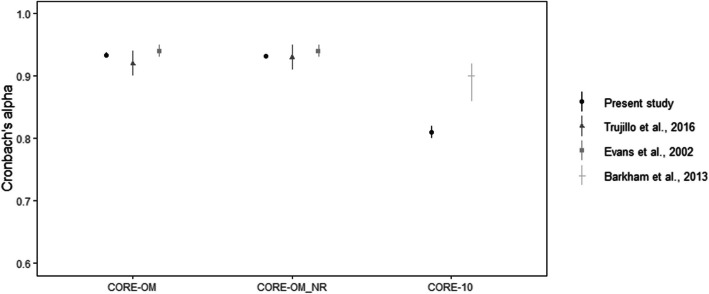
Observed Cronbach’s alpha and 95% confidence intervals reported by previous studies in the UK and Spain, as well as our data. Internal consistency was acceptable for all forms, with the CORE-10 exhibiting the lowest coefficients. The results of the present study are consistent with previous research, except for the CORE-10

Figure 2 displays the observed Cronbach’s alpha and 95% confidence intervals reported by previous studies conducted in the UK and Spain, along with our data. The comparisons are made with the available data of these studies. Cronbach’s alpha for the CORE-OM and its non-risk items is similar to the one reported by Trujillo et al, (2016) in Spain and Evans et al. (2002) in the UK, based on the overlap of the confidence intervals. By contrast, Cronbach’s alpha for the embedded CORE-10 items was significantly lower in our data than reported by Barkham et al. (2013) for the English version of teh CORE-10.

##### Test–retest stability

Overall, the correlations between scores at T1 and T2 were consistently above 0.70 for all forms. In the 1-week retest for the tranche “2012–2013”, the lowest correlation with T1 scores was 0.72 for the CORE-OM. In contrast, for the retest after 6 weeks in the tranche “2010–2011”, the lowest correlation with T1 scores was 0.59 for the CORE-SFA. Pearson’s correlations were lower for the longest period of 6 months; however, the broad confidence intervals indicate no statistically significant difference between the correlations across both assessment periods.

Stability was also assessed by calculating the mean difference between scores at T1 and T2, along with their confidence intervals. The confidence intervals for these mean differences included 0, indicating no difference between the assessment occasions for all forms. This lack of difference is evident for those who completed the retest at 1 week and 6 months. Table 4 presents the correlations, mean differences, and confidence intervals for the entire sample, as well as separately for the two tranches (1 week and 6 months). See the supplementary materials for scattergrams illustrating test–retest reliability for each form.

**Table 4:** Test–retest stability and changes of mean values between the first and second survey in a non-help-seeking sample

Form	Assessment	Stability	Change		
		R [95%CI]^a^	Mean [95%CI]^a^	Cohend1 [95%CI]^a^	Cohendz [95%CI]^a^
CORE-OM	1 week (*n* = 101)	0.81 [0.67, 0.89]	0.06 [0.00, 0.12]	0.13 [0.01, 0.26]	0.19 [− 0.01, 0.43]
	6 months (*n* = 65)	0.62 [0.43, 0.77]	− 0.03 [− 0.12, 0.05]	− 0.08 [− 0.33, 0.12]	− 0.09 [− 0.35, 0.17]
CORE-OM-NR	1 week (*n* = 101)	0.79 [0.68, 0.87]	0.08 [0.00, 0.15]	0.14 [0, 0.28]	0.21 [0, 0.44]
	6 months (*n* = 65)	0.63 [0.40, 0.78]	− 0.04 [− 0.14, 0.06]	− 0.1 [− 0.35, 0.11]	− 0.11 [− 0.36, 0.14]
CORE-SFA	1 week (*n* = 101)	0.78 [0.65, 0.88]	0.07 [0.00, 0.14]	0.14 [0, 0.29]	0.2 [0.01, 0.42]
	6 months (*n* = 65)	0.59 [0.37, 0.76]	− 0.05 [− 0.15, 0.04]	− 0.12 [− 0.38, 0.11]	− 0.13 [− 0.37, 0.12]
CORE-SFB	1 week (*n* = 101)	0.79 [0.67, 0.87]	0.06 [− 0.01, 0.14]	0.12 [− 0.01, 0.25]	0.18 [− 0.01, 0.38]
	6 months (*n* = 65)	0.61 [0.39, 0.77]	− 0.01 [− 0.10, 0.06]	− 0.02 [− 0.27, 0.18]	− 0.03 [− 0.29, 0.23]
GP-CORE	1 week (*n* = 101)	0.77 [0.67, 0.85]	0.07 [− 0.01, 0.14]	0.13 [− 0.02, 0.29]	0.17 [− 0.03, 0.39]
	6 months (*n* = 65)	0.65 [0.46, 0.79]	− 0.03 [− 0.12, 0.06]	− 0.07 [− 0.29, 0.14]	− 0.08 [− 0.35, 0.19]
CORE-10	1 week (*n* = 101)	0.72 [0.53, 0.85]	0.07 [− 0.01, 0.15]	0.13 [− 0.02, 0.29]	0.17 [− 0.04, 0.39]
	6 months (*n* = 65)	0.64 [0.48, 0.81]	− 0.05 [− 0.14, 0.03]	− 0.12 [− 0.37, 0.07]	− 0.14 [− 0.37, 0.11]

^a^Bootstrap confidence interval used the percentile method with 1000 bootstrap replications

In summary, the test–retest reliability was high for all forms, with the lowest correlation observed for the CORE-SFA at the 6-week retest. The stability analysis showed no significant differences between T1 and T2 scores, suggesting that the forms remained stable across both the 1-week and 6-month retests.

### Description of the scores

We compared mean scores based on sociodemographic characteristics, including gender (men vs. women), age (< 36 vs. > 35), and education (higher education vs. less than 12 years of education) for each form. We found 95% confidence intervals encompassing 0 for age, but not for gender and education, indicating significant differences in mean scores by gender and education (see supplementary material). Table 5 displays the mean and standard deviation for each form categorized by gender and education. Additional details regarding mean differences and plots of the mean scores can be found in the supplementary material.

**Table 5:** Descriptive statistics for each form, categorized by gender and education

Form		Gender (*n* = 1663)^*a*^		Education (*n* = 1651)^*a*^	
		Men (*n* = 681)	Women (*n* = 982)	Less than 12 years of education (*n* = 774)	Higher education (*n* = 877)
CORE-OM	Min–max	0.03–3.06	0–2.97	0–3.06	0.03–2.97
	Mean [95%CI]^b^	0.79 [0.75, 0.83]	0.89[0.86, 0.93]	0.91[0.87, 0.95]	0.80[0.77, 0.83]
	SD	0.49	0.52	0.54	0.47
CORE-OM-NR	Min–max	0.04–3.36	0–3.11	0–3.36	0–3.11
	Mean [95%CI]^b^	0.79 [0.76, 0.83]	0.89[0.86, 0.93]	1.07[1.02, 1.11]	0.95 [0.91, 0.99]
	SD	0.55	0.59	0.61	0.54
CORE-SFA	Min–max	0–2.89	0–3.17	0–2.89	0–3.17
	Mean [95%CI]^b^	0.79[0.76, 0.83]	0.89 [0.86, 0.93]	0.97[0.92, 1.01]	0.86 [0.82, 0.89]
	SD	0.52	0.56	0.58	0.52
CORE-SFB	Min–max	0–3.33	0.294	0–3.33	0–3.28
	Mean [95%CI]^b^	0.79[0.76, 0.83]	0.89 [0.86, 0.93]	1.02 [0.98, 1.06]	0.90[0.87, 0.94]
	SD	0.53	0.57	0.59	0.52
CORE-GP	Min–max	0–3.14	0–3.14	0–3.14	0–3
	Mean [95%CI]^b^	0.79[0.76, 0.83]	0.89 [0.86, 0.93]	1.16[1.12, 1.21]	1.05[1.02, 1.09]
	SD	0.55	0.58	0.60	0.53
CORE-10	Min–max	0–3.1	0–3.1	0–3.1	0–3
	Mean [95%CI]^b^	0.79[0.76, 0.83]	0.89 [0.86, 0.93]	0.89[0.85, 0.93]	0.78[0.75, 0.82]
	SD	0.53	0.57	0.59	0.53

^a^The total sample size was 1667. However, three participants did not report their gender (*n* = 1664), 15 did not report their education level (*n* = 1652), and one lacked the minimum information for score proration. This accounts for discrepancies in participant totals across groups

^b^Bootstrap confidence interval used the percentile method with 1000 bootstrap replications

In summary, significant differences in mean scores were found by gender and education, but not by age.

### Distributions of the scores

**Fig. 3:**
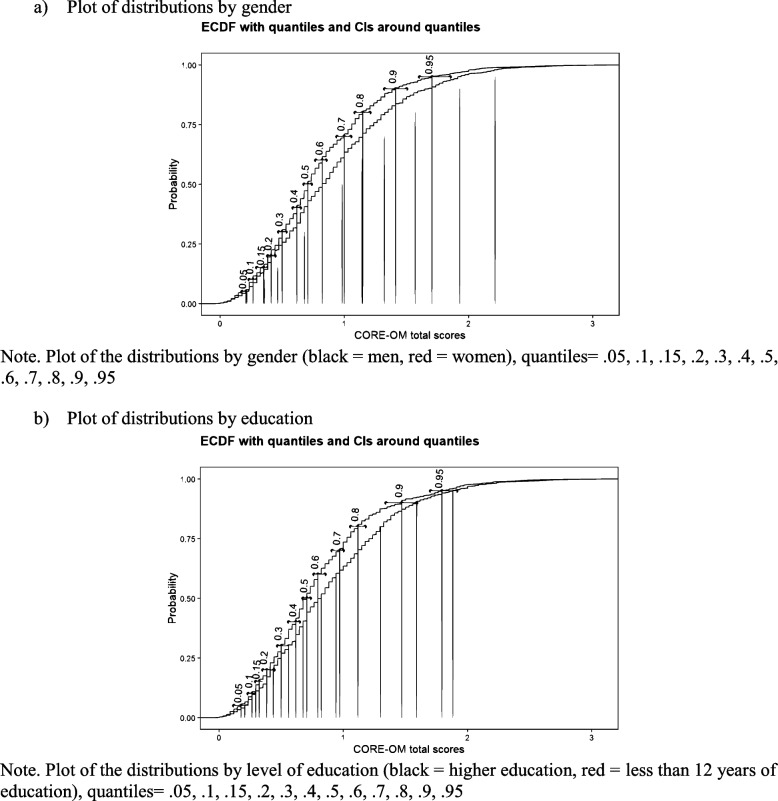
Plots of the distributions by gender and education. **a** Plot of distributions by gender. Note. Plot of the distributions by gender (black = men, red = women), quantiles = 0.05, 0.1, 0.15, 0.2, 0.3, 0.4, 0.5, 0.6, 0.7, 0.8, 0.9, 0.95. **b** Plot of distributions by education. Note. Plot of the distributions by level of education (black = higher education, red = less than 12 years of education), quantiles = 0.05, 0.1, 0.15, 0.2, 0.3, 0.4, 0.5, 0.6, 0.7, 0.8, 0.9, 0.95

Considering the two sociodemographic variables—gender and educational level—where significant mean differences were observed, we calculated and compared the score distributions for each form. All distributions are provided in the supplementary material. Figure 3 presents visual representations of these distributions.

## Discussion

The present study explores and describes the scores of various forms of the CORE-OM in a non-help-seeking sample of Spanish speakers living in Spain. This study expands upon the information presented in Trujillo et al. (2016) by utilizing a substantially larger non-help-seeking sample and providing reference scores for multiple CORE system forms. Furthermore, it offers detailed distributions by gender and education, providing valuable insights for researchers and clinicians interested in comparing their outcomes with those obtained from a non-help-seeking sample in Spain.

The sample was recruited over a 9-year period, indicating that the information is not specific to a single year, and variations between years were noted. The convenience sampling used in this study prevents us from categorizing this data as representative of the entire Spanish population; nevertheless, it provides insights into anticipated scores and psychometric properties within this population. We acknowledge that this sampling approach resulted in an overrepresentation of individuals with higher education levels, prompting us to advise caution when interpreting the results. However, we provided information about distributions by education in two categories: individuals with higher education and those without.

We did not collect information on the number of individuals approached to participate in the study, which prevents us from gauging the willingness to complete such surveys in Spain. Nonetheless, among those who consented to participate, we can affirm that the acceptability of completing the questionnaire was favorable; only one person did not complete the CORE-OM. Also, the omission rates were low, with less than 1% of participants omitting items at T1 and none at T2. The overall omission rate at T1 is comparable to the findings of Trujillo et al. (2016). However, in their study, the most frequently omitted items were item 3, “I have felt I have someone to turn to for support when needed,” and item, 25 “I have felt criticized by other people,” both from the Functioning domain and associated with interpersonal interactions. In contrast, our study identified item 34, “I have hurt myself physically or taken dangerous risks with my health,” which pertains to the risk domain, as the most omitted item. The reason for this difference is unclear, as the omission rates for these items were low in both studies, measuring less than 1%.

Regarding floor effects, we observed a notably high proportion of responses at the minimum value (i.e., 0) for items within the risk domain. These effects may be attributed to the nature of the population under study—a non-help-seeking population. The floor effects of risk scores in non-help-seeking populations can impact the measurement of the construct, potentially leading to an underestimation of psychological difficulties. To address this, Nonetheless, we have reported non-risk score distributions to address this issue and provide a more balanced perspective.

Regarding reliability, as anticipated, internal consistency diminishes in accordance with the number of items on the form (i.e., forms with a greater number of items exhibit higher levels of internal consistency). This highlights a key trade-off between brevity and psychometric robustness. Shorter measures like the CORE-10, while practical for clinical use, may compromise reliability by reducing the breadth of content coverage and inter-item correlations. Importantly, reliability may partially reflect the strengths and weaknesses of different CORE measures, providing a valuable metric for selecting forms based on specific research or clinical needs. For instance, the CORE-OM’s higher reliability underscores its suitability for more comprehensive assessments, while the CORE-10 offers a more pragmatic option for routine use, despite its relatively lower reliability (Valdiviezo-Oña et al., 2024).

Upon comparing Cronbach’s alpha coefficients from our study with those reported in studies conducted in Spain and the UK, we found no significant disparities concerning CORE-OM scores and CORE-OM-NR scores. However, a notable discrepancy emerged between our findings and those for the CORE-10 in the UK (Barkham et al., 2013). Although the UK sample is small, it is evident from Fig. 2 that the difference between these new Spanish data and the UK data is substantial and unlikely to be due to sampling variability. It seems highly likely that the difference is attributable to the very low rate of non-zero responses in risk items. This also underlines the need for language and culture/country-specific datasets and analyses and the dangers of assuming that psychometric statistics will be stable across languages and localities.

Moreover, the high alpha from the UK sample, with the relatively small size of 77, makes it possible that non-random sampling, which might have made that an unusually high value even for a UK help-seeking population. This highlights the need for further exploration of the internal reliability of the CORE-10, both in embedded and standalone formats, as well as across non-help-seeking populations both English and Spanish-speaking samples. Future research should also investigate whether the lower reliability in shorter forms like the CORE-10 affects their utility in clinical and non-clinical settings, particularly when assessing constructs like risk that rely on sufficient item coverage.

Another difference from the UK findings was that the correlations between assessment occasions were lower than those found in the UK and Spain for the same scores, although all values still reflected acceptable test–retest reliability. The mean shift between occasions in our data had confidence intervals encompassing zero for both the 1-week and 6-month intervals. This is similar to the findings of the earlier study conducted in Spain (with a 2-week interval) but different from the results of the UK study (with a 1-week interval), where the confidence intervals for differences did not include zero, aligned with the widely noted “test–retest artifact” (Durham et al., 2002). We suspect that linguistic and cultural nuances concerning how questionnaires are perceived and understood contribute to the strength of this mean shift. Nonetheless, it would be insightful to gather data from various countries to comprehend the impact of repeated assessment of psychological distress in non-help-seeking populations.

The significant differences observed in score distributions by gender and education suggest the need for further exploration of the underlying factors contributing to these variations. Gender differences in psychological scores may reflect societal constructions that shape emotional expression and the reporting of psychological distress (Compañ Felipe, 2024). For example, women may be more inclined to express and report emotional difficulties, while societal expectations of masculinity may discourage men from acknowledging emotional experiences, thus affecting how distress is perceived and reported. It is worth noting that this gender difference was not observed in the Trujillo et al. (2016) study, highlighting the importance of continuing to explore these differences within the Spanish population.

Differences by education level may arise from varying stress levels, coping mechanisms, or differences in mental health awareness (Sironi, 2012). Individuals with less formal education may face stressors related to socioeconomic status, which could impact their mental well-being and influence their scores on psychological assessments.

In conclusion, gender, education, and other demographic factors likely play an important role in shaping how psychological experiences are reported. These factors should be carefully considered when interpreting psychological scores, and future research could benefit from a more thorough investigation of the societal and psychological mechanisms underlying these differences.

### Limitations

While this study has provided valuable insights into the psychometric properties and score distributions of multiple forms within the CORE system for a non-help-seeking Spanish sample, it is essential to acknowledge certain limitations that may affect the interpretation of these findings.

As previously mentioned, the sample is not representative of the entire Spanish population, with individuals with higher education being notably overrepresented due to the use of convenience sampling. Nonetheless, we have included scores categorized by education for the benefit of clinicians and researchers. Given the non-representative nature of the sample, we strongly advise exercising caution when comparing your own data with the distributions presented in this article.

It is also important to note that the analysis of the various CORE family system forms is based on item extraction from the CORE-OM. Consequently, the findings may not fully capture the behavior of the forms when administered individually. Nonetheless, the data provide an approximation of score distributions and psychometric properties for less-explored, shorter forms such as the CORE-SFA, CORE-SFB, GP-CORE, and CORE-10—forms that may be preferred to reduce respondent burden when completing questionnaires. Further research, employing these forms in isolation, is necessary to confirm their efficacy within this specific population.

### Potential applications

The current study offers valuable insights into the application of the CORE-OM and its related forms in non-help-seeking populations, providing reference scores that are relevant in various professional settings.

For clinicians, these findings underscore the importance of considering population-specific characteristics when interpreting psychological distress scores. For example, the floor effects observed in risk items highlight the need for cautious interpretation in non-help-seeking populations, where the limited variability may obscure subtle signs of psychological distress. These score distributions serve as reference benchmarks for general population studies and as tools for mapping changes in clients across mental health interventions, relative to non-help-seeking score distributions.

In research contexts, the detailed breakdown of scores by gender and education offers a valuable framework for comparative studies. Researchers can use these reference scores to benchmark findings against similar populations or identify outliers that may reflect specific psychological trends in certain subgroups. Additionally, cut-off points for the calculation of clinically significant change (CSC) can be refined using this data in comparison with clinical samples. Moreover, the study’s findings on reliability and test–retest stability across different CORE forms provide essential guidance for researchers in selecting the most appropriate tool, balancing the trade-offs between brevity and psychometric rigor. These distributions also enable comparisons with other non-help-seeking samples and serve as benchmarks for evaluating changes in clients following mental health interventions.

## Conclusion

In conclusion, this study provides valuable insights into the performance of measures within the CORE system when applied to a non-help-seeking Spanish population. Our findings indicate that all forms in the system demonstrate reliability, showing commendable levels of internal consistency and test–retest reliability. Furthermore, we have presented distributions stratified by gender and education, which serve as a valuable resource for both researchers and clinicians. These distributions can be useful for comparative analyses with other non-help-seeking samples or as benchmarks for assessing the magnitude of change in clients following mental health interventions.

It is important to emphasize that our findings and data offer a preliminary approximation of the properties and distributions characterizing these various forms within the CORE system. We encourage invite further exploration and investigation in this area, as continued research efforts have the potential to enhance the applicability and effectiveness of the CORE system’s diverse forms within the Spanish population.

## Supplementary Information

Supplementary material 1: A comprehensive description of sociodemographic characteristics, reliability analyses and score distributions by tranche and form.

## Data Availability

The anonymized data collected is available at https://osf.io/bwnrm/?view_only=7475163c8dae4ecc88ddfae65718043f.

## Abbreviations

CORE: Clinical Outcomes in Routine Evaluation
CORE-OM: Clinical Outcomes in Routine Evaluation-Outcome Measure
CORE-OM-NR: Clinical Outcomes in Routine Evaluation-Outcome Measure Non-risk Items
CORE-SFA: Clinical Outcomes in Routine Evaluation Short Form A
CORE-SFB: Clinical Outcomes in Routine Evaluation Short Form B
CORE-10: Clinical Outcomes in Routine Evaluation 10
GP-CORE: Clinical Outcomes in Routine Evaluation for General Population
CI: Confidence interval
PPDAC: Problem, Plan, Data, Analyses, Conclusions and Communication
T1: Initial assessment
T2: Retest
