# Supplementary material for: Psychometric properties and score distributions of the Clinical Outcomes in Routine Evaluation measures within a non-help-seeking population from Spain
Source: Psicol Reflex Crit. 2025 Sep 2;38:25. doi: 10.1186/s41155-025-00341-6 (PMC12405070; doi:10.1186/s41155-025-00341-6)
Supplement: Supplementary file 1 — Supplementary material 1: A comprehensive description of sociodemographic characteristics, reliability analyses and score distributions by tranche and form. [file 41155_2025_341_MOESM1_ESM.pdf]

Supplementary material for: Psychometric properties and score distributions of the Clinical Outcomes in Routine Evaluation measures within a non-help-seeking population from Spain

- 1. Characteristics of the sample
  - Sociodemographic characteristics by tranche
    - Age
    - Gender
    - Level of Education
    - Employment status
    - Marital status
    - Nationality
    - Province
- 3. Reliability analyses
  - Internal consistency
    - By ocasions
    - Reliability by gender
    - Reliability by age
    - Reliability by level of education
    - Reliability by tranche
  - Test-Retest Reliability
    - Correlation total (any T2)
    - Correlation one week
    - Correlation six months
  - Mean Difference
  - Augmented scattergrams
    - CORE-OM
    - CORE-OM-NR
    - CORE-SFA
    - CORE-SFB
    - CORE-GP

        - CORE-10
- 4. Description of the scores
    - Total
    - By gender
    - By age
    - By level of education
- Distributions
    - By Gender
        - CORE-OM
        - CORE-OM-NR
        - CORE-SFA
        - CORE-SFB
        - GP-CORE
        - CORE-10
    - BY Education
        - CORE-OM
        - CORE-OM-NR
        - CORE-SFA
        - CORE-SFB
        - GP-CORE
        - CORE-10

The headings in this document are organized based on the order of presentation in the article's results section. Here, we expand upon the information provided in the article.

1. Characteristics of the sample

Sociodemographic characteristics by tranche

Age

Table presenting descriptives of age by tranche

	Total	2010-2011	2011-2012	2012-2013	2013-2014	2014-2015	2017-2018
n	1664.00	69.00	73.00	408.00	420.00	309.00	385.00
na	3.00	0.00	0.00	0.00	0.00	0.00	3.00
mean	37.16	36.99	36.71	36.48	36.31	37.18	38.90
sd	11.95	8.85	10.44	12.40	11.58	11.61	12.75
se	0.29	1.07	1.22	0.61	0.56	0.66	0.65
LCL	36.59	34.90	34.32	35.27	35.21	35.89	37.63
UCL	37.73	39.07	39.11	37.68	37.42	38.48	40.17
median	36.00	37.00	36.00	35.00	35.50	36.00	37.00
min	18.00	22.00	21.00	18.00	18.00	18.00	18.00

	Total	2010-2011	2011-2012	2012-2013	2013-2014	2014-2015	2017-2018
max	74.00	56.00	56.00	65.00	65.00	71.00	74.00
range	56.00	34.00	35.00	47.00	47.00	53.00	56.00
skewness	0.39	0.11	0.25	0.36	0.40	0.47	0.32
kurtosis	-0.81	-1.06	-1.17	-0.91	-0.85	-0.69	-0.94
LCLb	36.58	34.94	34.44	35.23	35.22	35.90	37.69
UCLb	37.77	39.32	39.01	37.60	37.37	38.57	40.13
Plot							

Gender

Table presenting descriptives of gender by tranche

	General	2010-2011	2011-2012	2012-2013	2013-2014	2014-2015	2017-2018
men	681 (40.9%)	39 (56.5%)	32 (43.8%)	173 (42.4%)	158 (37.6%)	105 (34%)	174 (45.2%)
women	983 (59.1%)	30 (43.5%)	41 (56.2%)	235 (57.6%)	262 (62.4%)	204 (66%)	211 (54.8%)
Missing	3	0	0	0	0	0	3

Percentage of women

Table showing the percentage of women by tranche

wave	total	women	percwomen	LCL	UCL
2010-2011	69	30	43.47826	32.43280	55.21160
2011-2012	73	41	56.16438	44.75778	66.95465
2012-2013	408	235	57.59804	52.75377	62.30057
2013-2014	420	262	62.38095	57.65551	66.88197
2014-2015	309	204	66.01942	60.57050	71.07492
2017-2018	385	211	54.80519	49.81078	59.70467

The total percentage of women is 59.07 with 95% CI from 56.69 to 61.41.

Plot

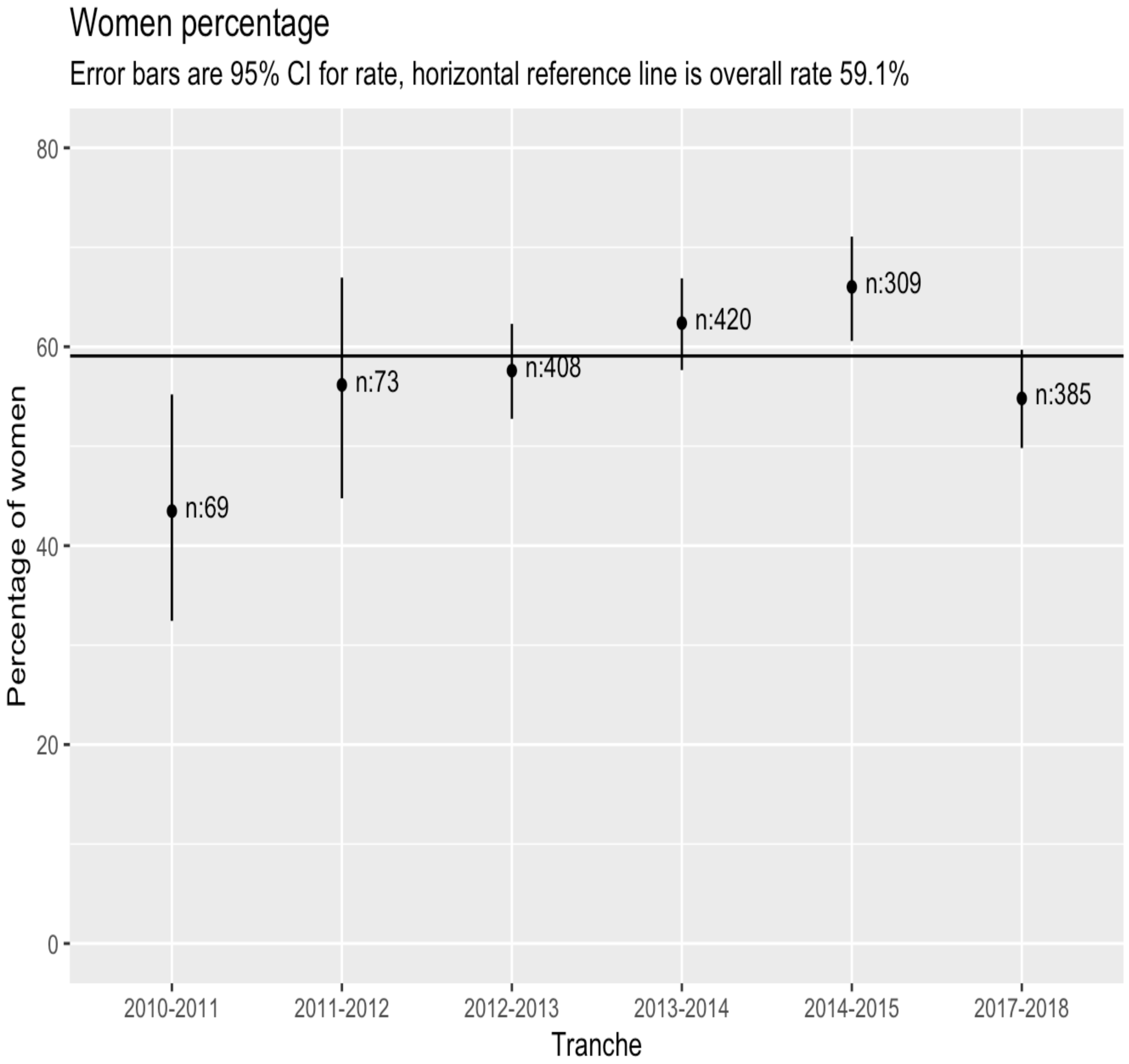

The chi-square test shows dependence between tranches and rates of women (X = 18.53(df = 5), p = 0.0023484)

That shows very clearly how large the differences between the tranches are in rates of women. Cramer's V for the association is 0.09 with 95% CI from 0 to 0.13.

Level of Education

Table presenting descriptives of gender by tranche

	General	2010-2011	2011-2012	2012-2013	2013-2014	2014-2015	2017-2018
elementary	128 (7.7%)	2 (2.9%)	4 (5.5%)	26 (6.5%)	29 (7%)	22 (7.1%)	45 (11.7%)
high_school	645 (39%)	16 (23.2%)	30 (41.1%)	180 (44.9%)	154 (36.9%)	114 (36.9%)	151 (39.4%)
higher_education	877 (53.1%)	51 (73.9%)	39 (53.4%)	194 (48.4%)	233 (55.9%)	173 (56%)	187 (48.8%)
no_education	2 (0.1%)	0 (0%)	0 (0%)	1 (0.2%)	1 (0.2%)	0 (0%)	0 (0%)
Missing	15	0	0	7	3	0	5

Percentage of participants with higher education

Table showing the percentage of participants with higher education by tranche

wave	total	higher	perchigher	LCL	UCL
2010-2011	69	51	73.91304	62.48943	82.81444
2011-2012	73	39	53.42466	42.09895	64.40796
2012-2013	401	194	48.37905	43.52645	53.26242
2013-2014	417	233	55.87530	51.07742	60.56592
2014-2015	309	173	55.98705	50.41232	61.41476
2017-2018	383	187	48.82507	43.85555	53.81792

The total percentage of participants with higher education is 53.09 with 95% CI from 50.68 to 55.48.

Plot

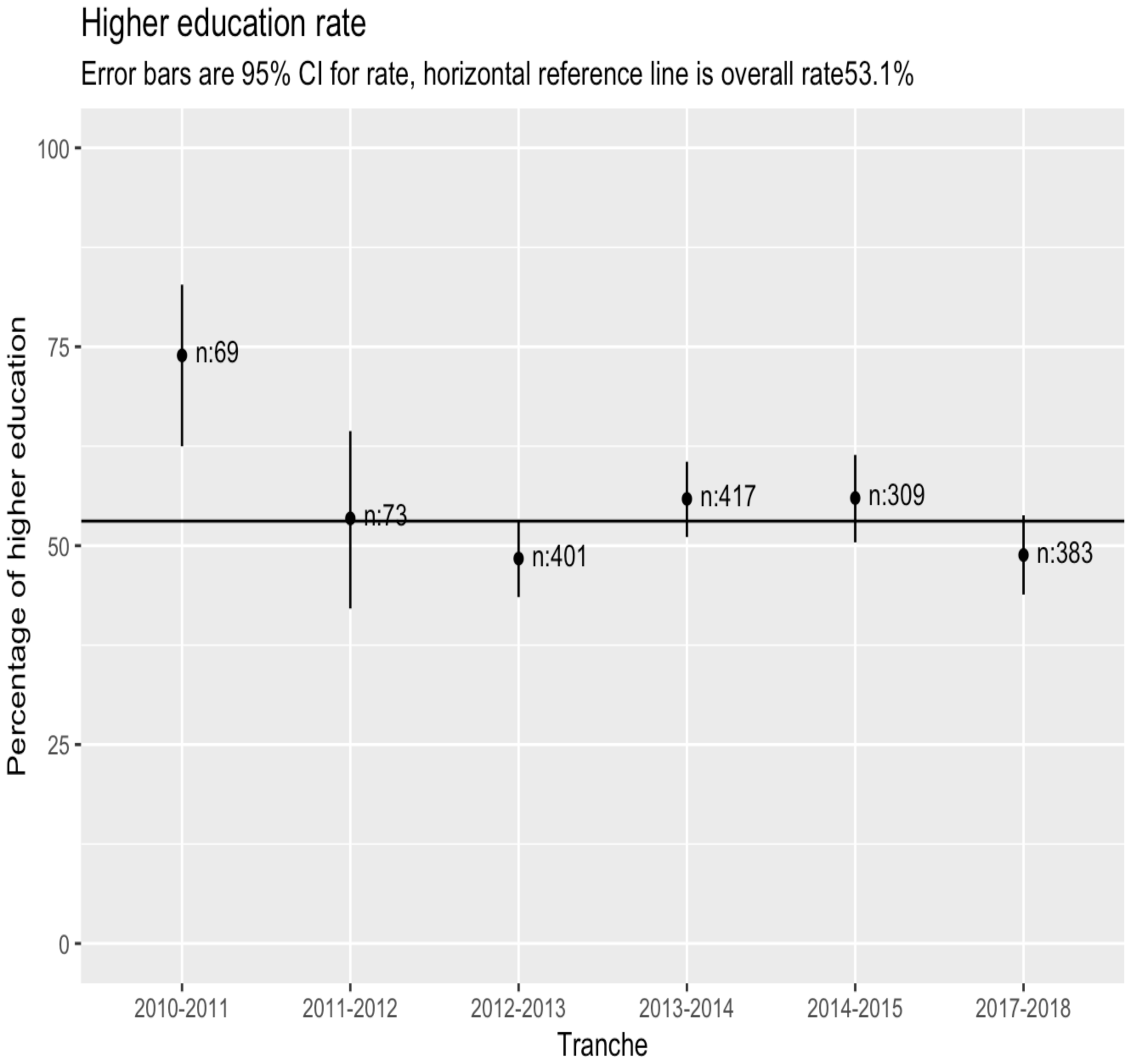

The chi-square test shows dependence between tranches and rates of women (X = 20.73(df = 5), p = 9.1194543^{-4})

That shows very clearly how large the differences between the waves are in rates of higher education. Cramer's V for the association is 0.1 with 95% CI from 0 to 0.14.

Employment status

Table presenting descriptives of employement status by tranche

	General	2010-2011	2011-2012	2012-2013	2013-2014	2014-2015	2017-2018
employed	906 (55.3%)	40 (74.1%)	48 (65.8%)	125 (31.2%)	248 (59.3%)	182 (59.1%)	263 (68.5%)
housework	98 (6%)	1 (1.9%)	4 (5.5%)	48 (12%)	17 (4.1%)	13 (4.2%)	15 (3.9%)
retired	25 (1.5%)	0 (0%)	0 (0%)	5 (1.2%)	7 (1.7%)	5 (1.6%)	8 (2.1%)
sick_leave	8 (0.5%)	0 (0%)	0 (0%)	4 (1%)	3 (0.7%)	1 (0.3%)	0 (0%)
studying	353 (21.6%)	7 (13%)	7 (9.6%)	162 (40.4%)	75 (17.9%)	52 (16.9%)	50 (13%)
unemployment	248 (15.1%)	6 (11.1%)	14 (19.2%)	57 (14.2%)	68 (16.3%)	55 (17.9%)	48 (12.5%)
Missing	29	15	0	7	2	1	4

Percentage of employed participants

Table showing the percentage of participants who are employed by tranche

wave	total	employed	percemployed	LCL	UCL
2010-2011	54	40	74.07407	61.06911	83.88135
2011-2012	73	48	65.75342	54.32663	75.60513
2012-2013	401	125	31.17207	26.83517	35.86628
2013-2014	418	248	59.33014	54.55684	63.93352
2014-2015	308	182	59.09091	53.52080	64.43704
2017-2018	384	263	68.48958	63.67944	72.93346

The total percentage of employed participants is 55.31 with 95% CI from 52.89 to 57.7.

Plot

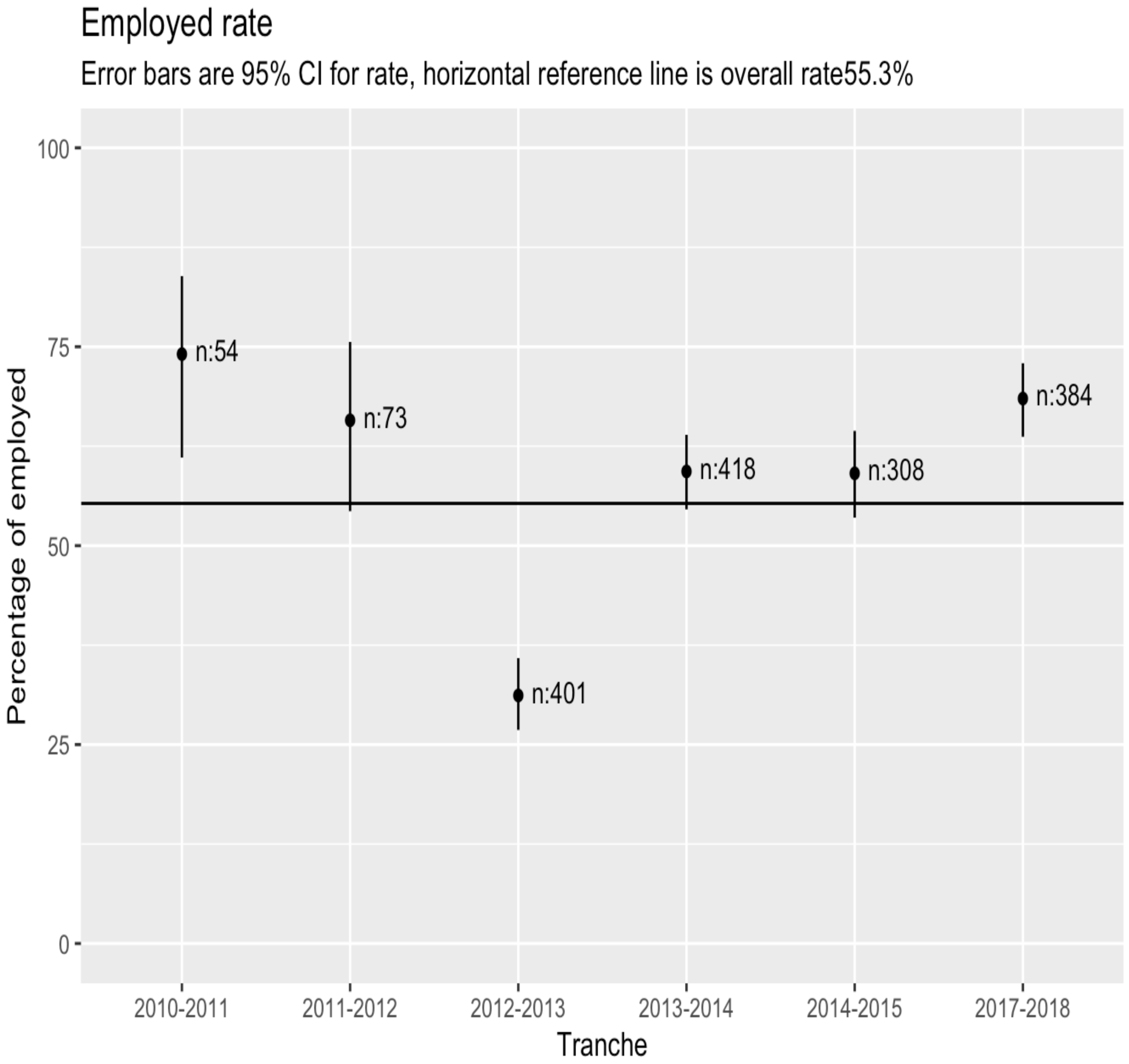

The chi-square test shows dependence between tranches and rates of women (X = 136.93(df = 5), p = 8.0194427^{-28})

That shows very clearly how large the differences between the waves are in rates of higher education. Cramer's V for the association is 0.28 with 95% CI from 0.23 to 0.33.

Marital status

Table presenting descriptives of marital status by tranche

	General	2010-2011	2011-2012	2012-2013	2013-2014	2014-2015	2017-2018
married	629 (38.1%)	23 (33.3%)	24 (32.9%)	138 (34.4%)	143 (34.3%)	145 (47.1%)	156 (40.6%)
separated/divorced	154 (9.3%)	9 (13%)	7 (9.6%)	31 (7.7%)	38 (9.1%)	37 (12%)	32 (8.3%)
single	854 (51.7%)	37 (53.6%)	42 (57.5%)	228 (56.9%)	230 (55.2%)	125 (40.6%)	192 (50%)
widowed	15 (0.9%)	0 (0%)	0 (0%)	4 (1%)	6 (1.4%)	1 (0.3%)	4 (1%)
Missing	15	0	0	7	3	1	4

Percentage of married participants

Table showing the percentage of participants who are married by tranche

wave	total	married	percmarried	LCL	UCL
2010-2011	69	23	33.33333	23.35105	45.07352
2011-2012	73	24	32.87671	23.19449	44.27099
2012-2013	401	138	34.41397	29.93165	39.19206
2013-2014	417	143	34.29257	29.89848	38.97341
2014-2015	308	145	47.07792	41.57382	52.65402
2017-2018	384	156	40.62500	35.82911	45.60661

The total percentage of married participants is 38.08 with 95% CI from 35.76 to 40.44.

Plot

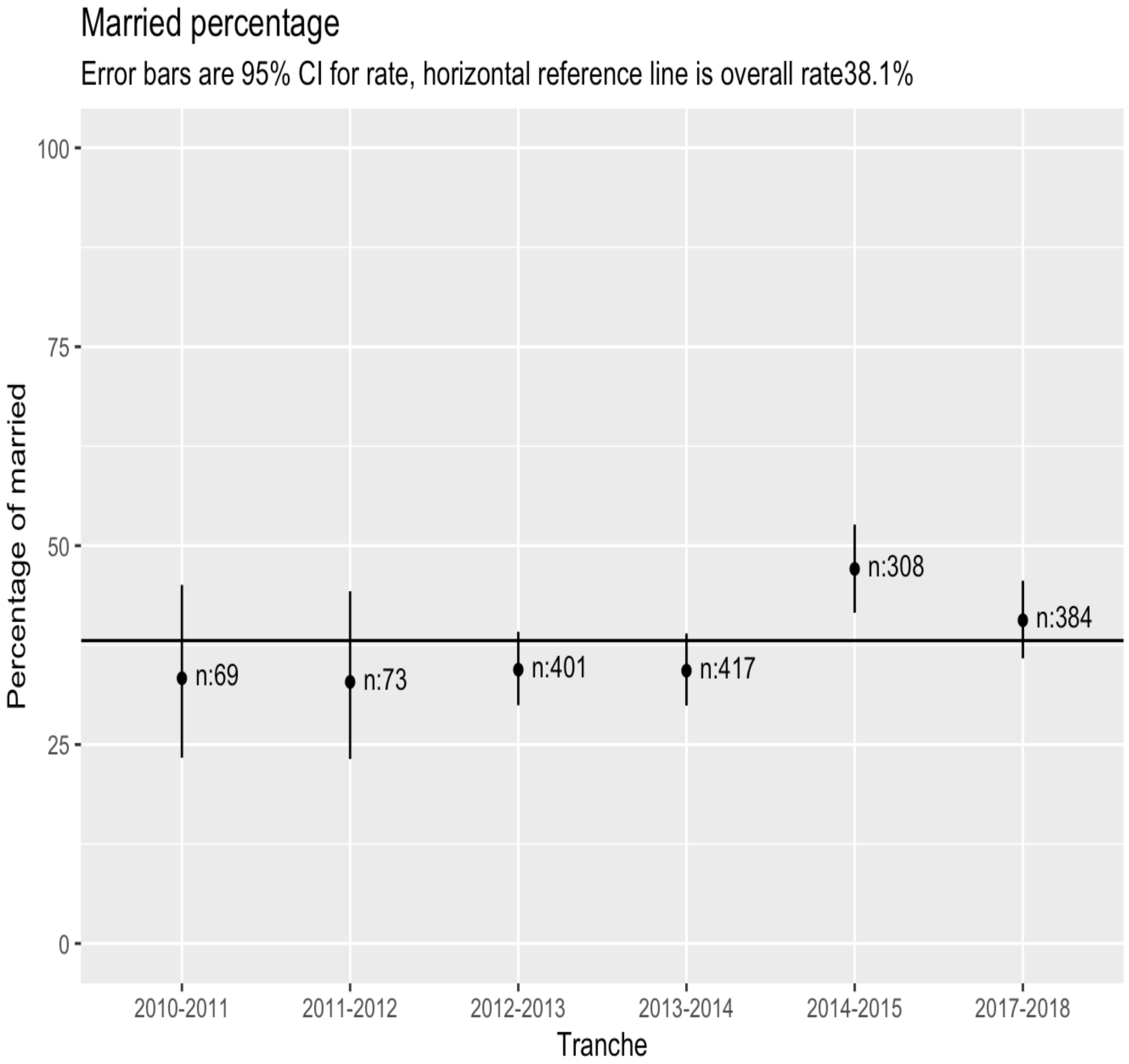

The chi-square test shows dependence between tranches and rates of women (X = 17.95(df = 5), p = 0.0030079)

That shows very clearly how large the differences between the waves are in rates of higher education. Cramer's V for the association is 0.09 with 95% CI from 0 to 0.13.

Nationality

Table presenting descriptives of the nationality of the participants by tranche

	General	2010-2011	2011-2012	2012-2013	2013-2014	2014-2015	2017-2018
Andorrana	3 (0.2%)	0 (0%)	0 (0%)	0 (0%)	0 (0%)	0 (0%)	3 (0.8%)
Argentina	5 (0.3%)	0 (0%)	0 (0%)	2 (0.5%)	3 (0.7%)	0 (0%)	0 (0%)
Bulgara	2 (0.1%)	0 (0%)	0 (0%)	0 (0%)	0 (0%)	0 (0%)	2 (0.5%)
Checo	1 (0.1%)	0 (0%)	0 (0%)	0 (0%)	0 (0%)	0 (0%)	1 (0.3%)
Chilena	1 (0.1%)	0 (0%)	0 (0%)	0 (0%)	0 (0%)	1 (0.3%)	0 (0%)
Colombiana	9 (0.5%)	0 (0%)	1 (1.4%)	0 (0%)	4 (1%)	3 (1%)	1 (0.3%)
Dominicana	1 (0.1%)	0 (0%)	0 (0%)	0 (0%)	0 (0%)	1 (0.3%)	0 (0%)
Espanola	1607 (97%)	69 (100%)	71 (97.3%)	400 (99.3%)	402 (96.2%)	293 (94.8%)	372 (96.6%)
Finlandesa	1 (0.1%)	0 (0%)	0 (0%)	0 (0%)	1 (0.2%)	0 (0%)	0 (0%)
Francesa	4 (0.2%)	0 (0%)	0 (0%)	1 (0.2%)	0 (0%)	3 (1%)	0 (0%)
Holandesa	1 (0.1%)	0 (0%)	0 (0%)	0 (0%)	0 (0%)	1 (0.3%)	0 (0%)
Italiana	5 (0.3%)	0 (0%)	0 (0%)	0 (0%)	2 (0.5%)	3 (1%)	0 (0%)
Letona	2 (0.1%)	0 (0%)	0 (0%)	0 (0%)	0 (0%)	2 (0.6%)	0 (0%)
Marroqui	1 (0.1%)	0 (0%)	0 (0%)	0 (0%)	0 (0%)	0 (0%)	1 (0.3%)
Mexicana	3 (0.2%)	0 (0%)	0 (0%)	0 (0%)	3 (0.7%)	0 (0%)	0 (0%)
Nicaraguense	1 (0.1%)	0 (0%)	0 (0%)	0 (0%)	1 (0.2%)	0 (0%)	0 (0%)
Paraguaya	1 (0.1%)	0 (0%)	0 (0%)	0 (0%)	0 (0%)	1 (0.3%)	0 (0%)
Peruana	1 (0.1%)	0 (0%)	0 (0%)	0 (0%)	0 (0%)	0 (0%)	1 (0.3%)
Portuguesa	1 (0.1%)	0 (0%)	0 (0%)	0 (0%)	1 (0.2%)	0 (0%)	0 (0%)
Rumana	1 (0.1%)	0 (0%)	0 (0%)	0 (0%)	0 (0%)	1 (0.3%)	0 (0%)
Rusa	1 (0.1%)	0 (0%)	0 (0%)	0 (0%)	1 (0.2%)	0 (0%)	0 (0%)
Uruguaya	1 (0.1%)	0 (0%)	1 (1.4%)	0 (0%)	0 (0%)	0 (0%)	0 (0%)
Venezolana	4 (0.2%)	0 (0%)	0 (0%)	0 (0%)	0 (0%)	0 (0%)	4 (1%)
Missing	10	0	0	5	2	0	3

Percentage of Spanish participants

Table showing the percentage of participants who are Spanish by tranche

wave	total	spanish	percspanish	LCL	UCL
2010-2011	69	69	100.00000	94.72627	100.00000
2011-2012	73	71	97.26027	90.54985	99.24542
2012-2013	403	400	99.25558	97.83449	99.74651
2013-2014	418	402	96.17225	93.87320	97.63037
2014-2015	309	293	94.82201	91.75530	96.78795
2017-2018	385	372	96.62338	94.30930	98.01625

The total percentage of Spanish participants is 96.98 with 95% CI from 96.04 to 97.7.

Plot

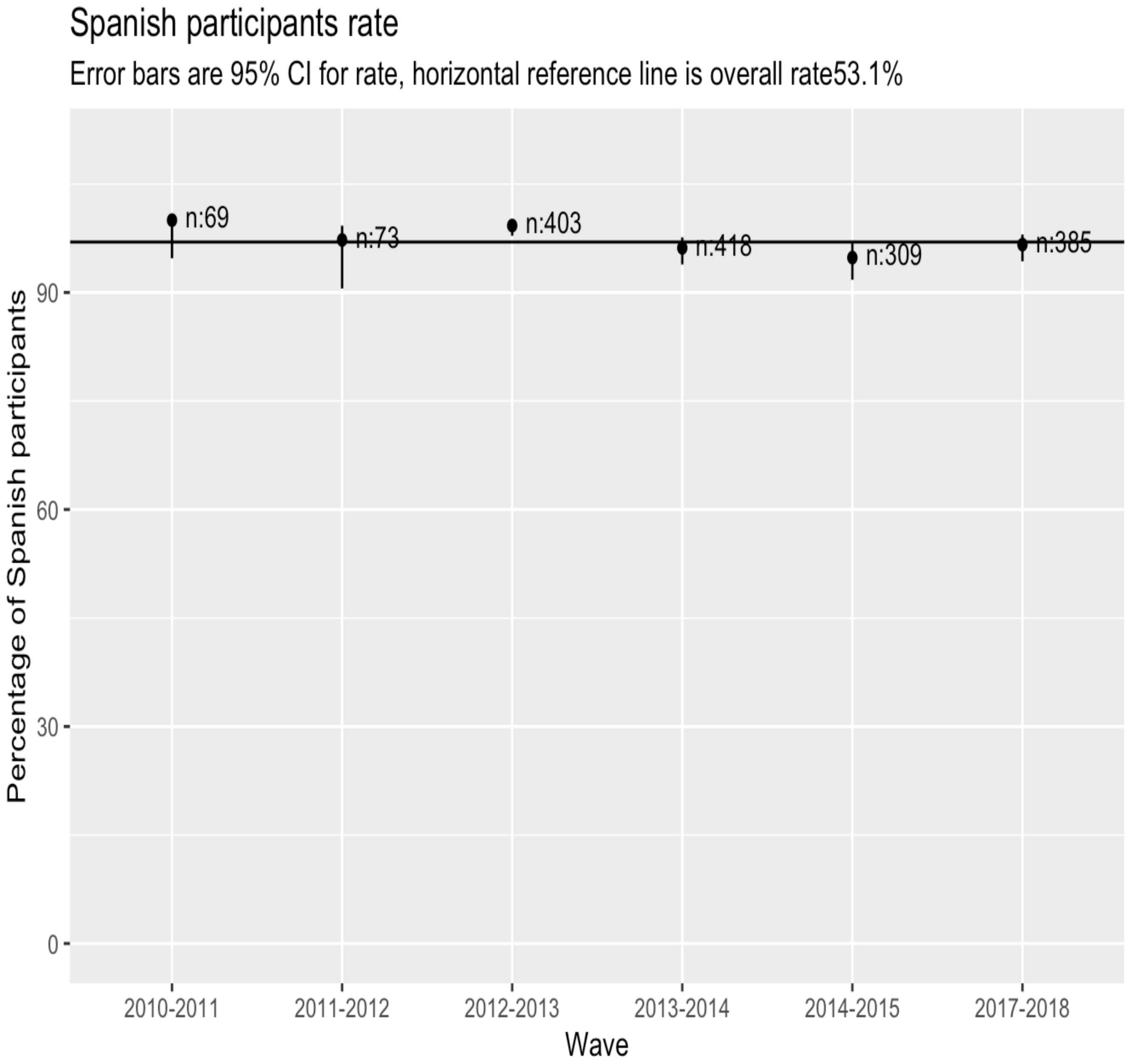

The chi-square test shows dependence between tranches and rates of women (X = 15.32(df = 5), p = 0.0090887)

That shows very clearly how large the differences between the waves are in rates of higher education. Cramer's V for the association is 0.08 with 95% CI from 0 to 0.12.

Province

Table presenting descriptives of the province of the participants by tranche

	General	2010-2011	2011-2012	2012-2013	2013-2014	2014-2015	2017-2018
Alava	4 (0.3%)	2 (3%)	0 (0%)	0 (0%)	0 (0%)	0 (0%)	2 (0.5%)
Albacete	39 (2.4%)	1 (1.5%)	2 (2.7%)	10 (2.5%)	12 (3%)	5 (1.7%)	9 (2.5%)
Alicante	34 (2.1%)	4 (6%)	2 (2.7%)	10 (2.5%)	7 (1.8%)	5 (1.7%)	6 (1.6%)
Almeria	12 (0.8%)	0 (0%)	0 (0%)	3 (0.8%)	6 (1.5%)	0 (0%)	3 (0.8%)
Asturias	14 (0.9%)	1 (1.5%)	2 (2.7%)	0 (0%)	2 (0.5%)	6 (2%)	3 (0.8%)
Avila	3 (0.2%)	0 (0%)	0 (0%)	0 (0%)	0 (0%)	3 (1%)	0 (0%)
Badajoz	19 (1.2%)	0 (0%)	3 (4.1%)	0 (0%)	11 (2.8%)	5 (1.7%)	0 (0%)
Barcelona	64 (4%)	2 (3%)	2 (2.7%)	24 (6%)	8 (2%)	13 (4.4%)	15 (4.1%)
Bizkaia	49 (3.1%)	1 (1.5%)	0 (0%)	21 (5.3%)	9 (2.3%)	5 (1.7%)	13 (3.6%)
Burgos	18 (1.1%)	0 (0%)	1 (1.4%)	0 (0%)	0 (0%)	0 (0%)	17 (4.7%)
Caceres	8 (0.5%)	0 (0%)	3 (4.1%)	2 (0.5%)	0 (0%)	0 (0%)	3 (0.8%)
Cadiz	35 (2.2%)	4 (6%)	7 (9.6%)	5 (1.3%)	4 (1%)	3 (1%)	12 (3.3%)
Cantabria	35 (2.2%)	2 (3%)	4 (5.5%)	0 (0%)	9 (2.3%)	12 (4%)	8 (2.2%)
Ciudad Real	27 (1.7%)	1 (1.5%)	0 (0%)	0 (0%)	3 (0.8%)	23 (7.7%)	0 (0%)
Cordoba	28 (1.8%)	1 (1.5%)	0 (0%)	17 (4.3%)	4 (1%)	6 (2%)	0 (0%)
Cuenca	4 (0.3%)	1 (1.5%)	0 (0%)	0 (0%)	0 (0%)	3 (1%)	0 (0%)
Girona	4 (0.3%)	0 (0%)	1 (1.4%)	0 (0%)	0 (0%)	0 (0%)	3 (0.8%)
Granada	17 (1.1%)	0 (0%)	0 (0%)	4 (1%)	5 (1.3%)	2 (0.7%)	6 (1.6%)
Guadalajara	16 (1%)	0 (0%)	0 (0%)	1 (0.3%)	6 (1.5%)	3 (1%)	6 (1.6%)
Guipuzcoa	25 (1.6%)	1 (1.5%)	1 (1.4%)	5 (1.3%)	9 (2.3%)	0 (0%)	9 (2.5%)
Huelva	3 (0.2%)	0 (0%)	0 (0%)	0 (0%)	0 (0%)	0 (0%)	3 (0.8%)
Huesca	3 (0.2%)	0 (0%)	0 (0%)	0 (0%)	3 (0.8%)	0 (0%)	0 (0%)
Islas Baleares	26 (1.6%)	1 (1.5%)	0 (0%)	2 (0.5%)	5 (1.3%)	9 (3%)	9 (2.5%)
Jaen	12 (0.8%)	0 (0%)	0 (0%)	5 (1.3%)	4 (1%)	0 (0%)	3 (0.8%)
La Coruna	31 (1.9%)	2 (3%)	2 (2.7%)	5 (1.3%)	8 (2%)	8 (2.7%)	6 (1.6%)
La Rioja	6 (0.4%)	0 (0%)	0 (0%)	0 (0%)	0 (0%)	3 (1%)	3 (0.8%)
Las Palmas	47 (2.9%)	1 (1.5%)	0 (0%)	15 (3.8%)	15 (3.8%)	7 (2.3%)	9 (2.5%)
Leon	22 (1.4%)	1 (1.5%)	3 (4.1%)	3 (0.8%)	3 (0.8%)	6 (2%)	6 (1.6%)
Lleida	4 (0.3%)	0 (0%)	0 (0%)	0 (0%)	1 (0.3%)	3 (1%)	0 (0%)
Lugo	12 (0.8%)	0 (0%)	0 (0%)	2 (0.5%)	1 (0.3%)	3 (1%)	6 (1.6%)
Madrid	403 (25.2%)	24 (35.8%)	22 (30.1%)	114 (28.6%)	92 (23.1%)	80 (26.8%)	71 (19.5%)
Malaga	28 (1.8%)	0 (0%)	5 (6.8%)	0 (0%)	11 (2.8%)	6 (2%)	6 (1.6%)
Melilla	5 (0.3%)	0 (0%)	0 (0%)	5 (1.3%)	0 (0%)	0 (0%)	0 (0%)
Murcia	34 (2.1%)	1 (1.5%)	0 (0%)	8 (2%)	9 (2.3%)	7 (2.3%)	9 (2.5%)
Navarra	49 (3.1%)	0 (0%)	0 (0%)	10 (2.5%)	3 (0.8%)	12 (4%)	24 (6.6%)
Ourense	7 (0.4%)	1 (1.5%)	1 (1.4%)	0 (0%)	2 (0.5%)	0 (0%)	3 (0.8%)
Palencia	10 (0.6%)	3 (4.5%)	0 (0%)	7 (1.8%)	0 (0%)	0 (0%)	0 (0%)
Pontevedra	54 (3.4%)	0 (0%)	0 (0%)	21 (5.3%)	17 (4.3%)	12 (4%)	4 (1.1%)
Salamanca	9 (0.6%)	0 (0%)	0 (0%)	6 (1.5%)	3 (0.8%)	0 (0%)	0 (0%)
Segovia	2 (0.1%)	1 (1.5%)	0 (0%)	0 (0%)	0 (0%)	1 (0.3%)	0 (0%)
Sevilla	71 (4.4%)	2 (3%)	3 (4.1%)	20 (5%)	16 (4%)	9 (3%)	21 (5.8%)
Soria	5 (0.3%)	0 (0%)	0 (0%)	0 (0%)	0 (0%)	0 (0%)	5 (1.4%)
Tarragona	6 (0.4%)	0 (0%)	0 (0%)	0 (0%)	6 (1.5%)	0 (0%)	0 (0%)
Tenerife	23 (1.4%)	2 (3%)	1 (1.4%)	10 (2.5%)	10 (2.5%)	0 (0%)	0 (0%)

	General	2010-2011	2011-2012	2012-2013	2013-2014	2014-2015	2017-2018
Teruel	3 (0.2%)	0 (0%)	1 (1.4%)	0 (0%)	2 (0.5%)	0 (0%)	0 (0%)
Toledo	30 (1.9%)	0 (0%)	0 (0%)	13 (3.3%)	8 (2%)	3 (1%)	6 (1.6%)
Valencia	64 (4%)	0 (0%)	2 (2.7%)	9 (2.3%)	28 (7%)	7 (2.3%)	18 (4.9%)
Valladolid	17 (1.1%)	0 (0%)	0 (0%)	8 (2%)	6 (1.5%)	3 (1%)	0 (0%)
zamora	3 (0.2%)	0 (0%)	0 (0%)	3 (0.8%)	0 (0%)	0 (0%)	0 (0%)
Zamora	10 (0.6%)	0 (0%)	0 (0%)	4 (1%)	0 (0%)	0 (0%)	6 (1.6%)
Zaragoza	145 (9.1%)	7 (10.4%)	5 (6.8%)	27 (6.8%)	50 (12.6%)	25 (8.4%)	31 (8.5%)
Missing	68	2	0	9	22	11	24

Percentage of participants from Madrid

Table showing the percentage of participants who live in Madrid by tranche

wave	total	madrid	percmadrid	LCL	UCL
2010-2011	67	24	35.82090	25.39806	47.78149
2011-2012	73	22	30.13699	20.82259	41.43737
2012-2013	399	114	28.57143	24.35957	33.19197
2013-2014	398	92	23.11558	19.24273	27.50244
2014-2015	298	80	26.84564	22.13227	32.14837
2017-2018	364	71	19.50549	15.76216	23.88575

total	madrid	percmadrid	LCL	UCL
1599	403	25.20325	23.1363	27.38907

The total percentage of participants living in Madrid is 25.2 with 95% CI from 23.14 to 27.39.

Plot

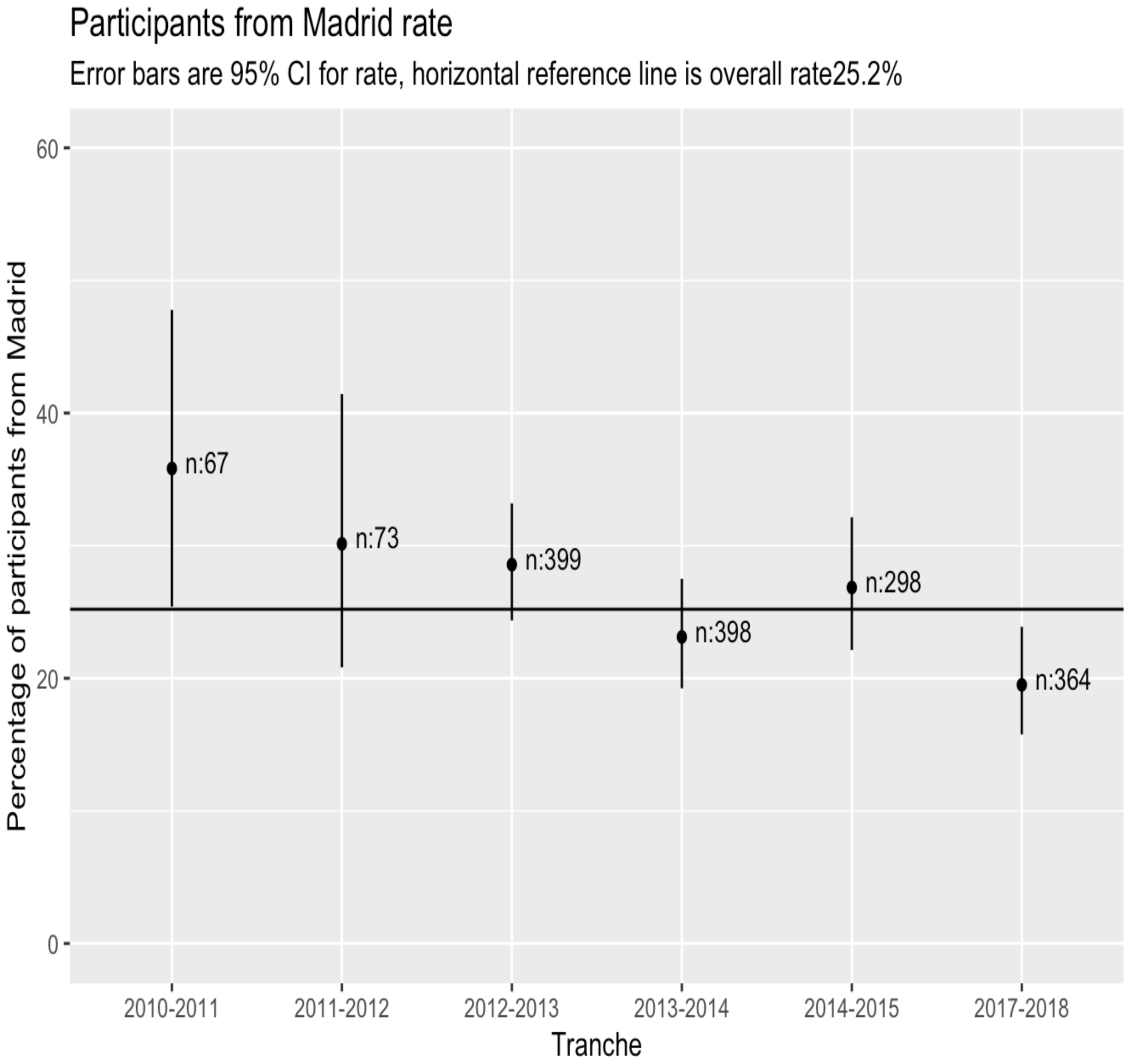

The chi-square test shows dependence between tranches and rates of women (X = 14.97(df = 5), p = 0.0105099)

That shows very clearly how large the differences between the waves are in rates of higher education. Cramer’s V for the association is 0.08 with 95% CI from 0 to 0.12.

3. Reliability analyses

Internal consistency

By ocasions

T1

Table of internal consistency coefficients by form at T1

	alpha	LCI	UCI	bLCI	bUCI	omega
CORE-OM	0.93	0.93	0.94	0.93	0.94	0.94
CORE-OM-NR	0.93	0.93	0.94	0.93	0.94	0.94
CORE-SFA	0.90	0.89	0.90	0.89	0.90	0.91
CORE-SFB	0.88	0.88	0.89	0.87	0.89	0.90
CORE-GP	0.85	0.84	0.86	0.84	0.86	0.88
CORE-10	0.81	0.80	0.82	0.79	0.83	0.84

T2

Table of internal consistency coefficients by form at T2

	alpha	LCI	UCI	bLCI	bUCI	omega
CORE-OM	0.94	0.93	0.95	0.92	0.95	0.95
CORE-OM-NR	0.94	0.92	0.95	0.91	0.95	0.95
CORE-SFA	0.91	0.89	0.92	0.88	0.93	0.92
CORE-SFB	0.88	0.86	0.90	0.85	0.91	0.91
CORE-GP	0.85	0.83	0.88	0.81	0.88	0.89
CORE-10	0.83	0.80	0.86	0.77	0.87	0.87

Plot of Cronbach’s alpha and 95%CI by form at T1 and T2

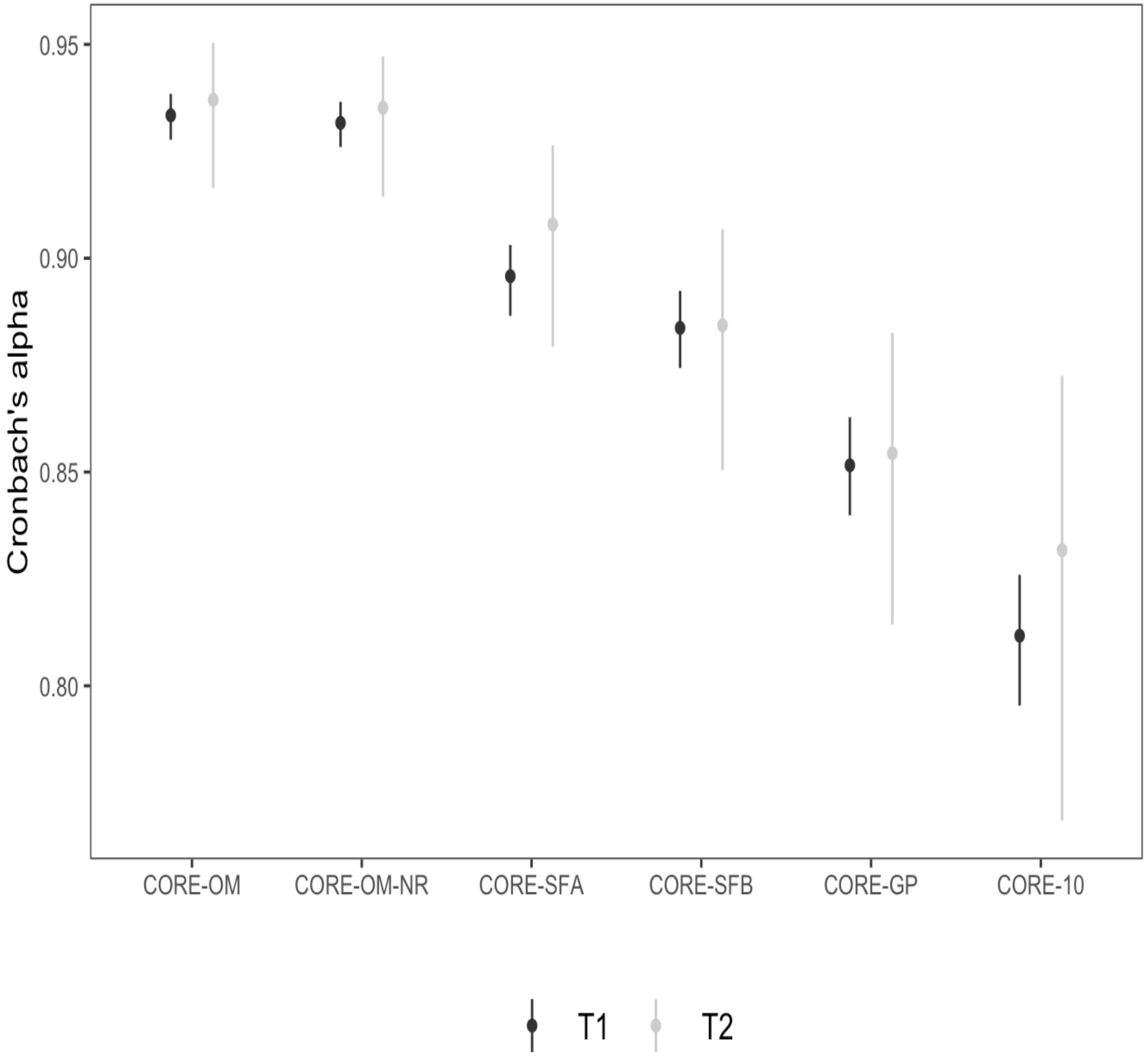

Reliability by gender

Table of internal consistency coefficients by form at T1 and by gender

	alpha	LCI	UCI	bLCI	bUCI	omega	gender
CORE-OM	0.93	0.92	0.94	0.92	0.94	0.94	men
CORE-OM-NR	0.93	0.92	0.93	0.91	0.93	0.94	men
CORE-SFA	0.89	0.88	0.90	0.87	0.90	0.91	men
CORE-SFB	0.87	0.86	0.88	0.85	0.89	0.89	men
CORE-GP	0.84	0.82	0.85	0.82	0.86	0.87	men

	alpha	LCI	UCI	bLCI	bUCI	omega	gender
CORE-10	0.80	0.78	0.82	0.76	0.82	0.84	men
CORE-OM1	0.94	0.93	0.94	0.93	0.94	0.95	women
CORE-OM-NR1	0.93	0.93	0.94	0.93	0.94	0.94	women
CORE-SFA1	0.90	0.89	0.91	0.89	0.91	0.91	women
CORE-SFB1	0.89	0.88	0.90	0.88	0.90	0.90	women
CORE-GP1	0.86	0.85	0.87	0.84	0.87	0.89	women
CORE-101	0.82	0.80	0.83	0.80	0.84	0.85	women

Plot of Cronbach's alpha and 95%CI by form and gender at T1

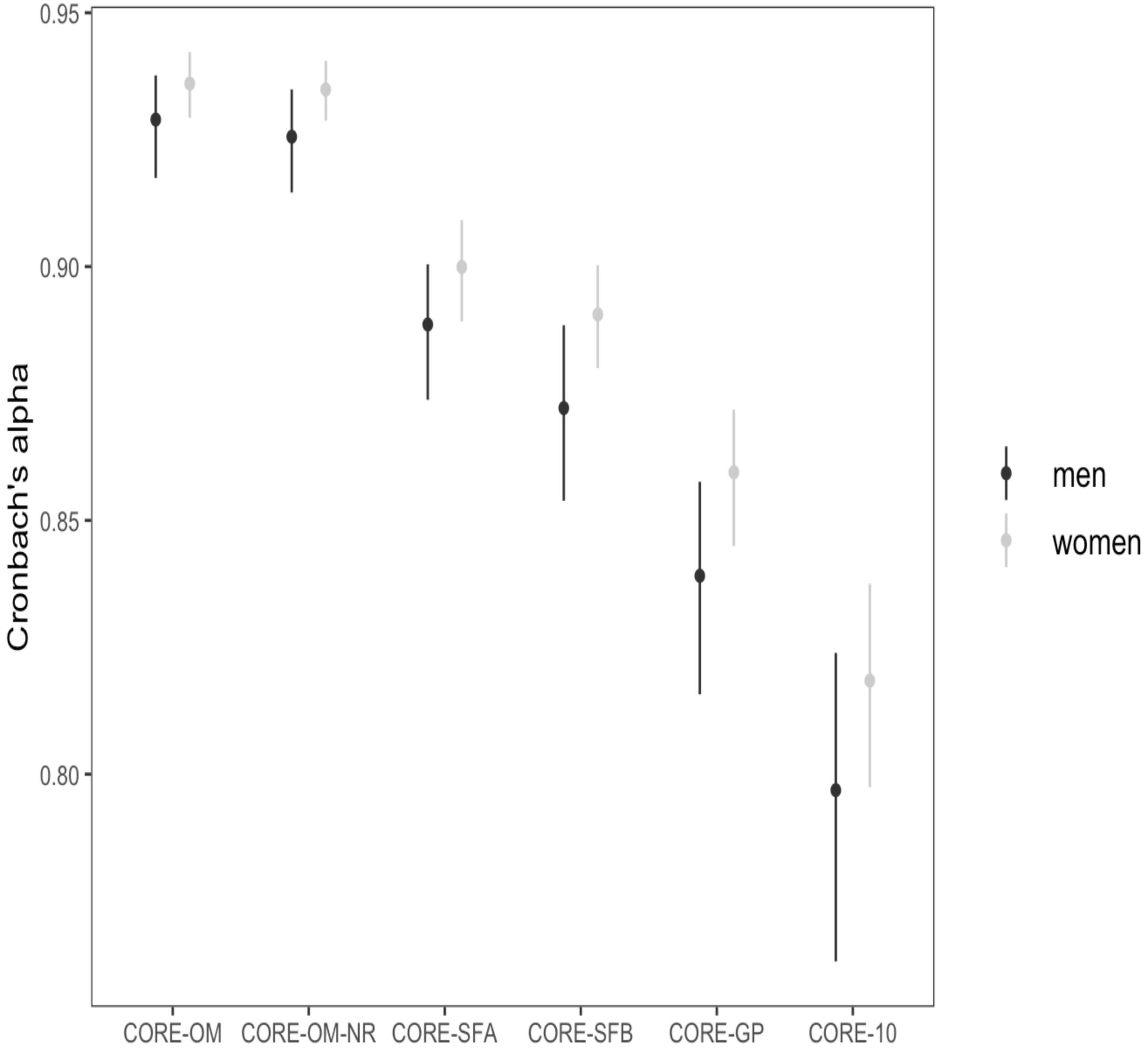

Reliability by age

Table of internal consistency coefficients by form at T1 and by age

	**alpha**	**LCI**	**UCI**	**bLCI**	**bUCI**	**omega**	**age**
CORE-OM	0.93	0.93	0.94	0.93	0.94	0.94	<36
CORE-OM-NR	0.93	0.93	0.94	0.92	0.94	0.94	<36
CORE-SFA	0.90	0.89	0.91	0.89	0.91	0.91	<36
CORE-SFB	0.88	0.87	0.89	0.87	0.89	0.90	<36
CORE-GP	0.85	0.83	0.86	0.83	0.86	0.88	<36
CORE-10	0.81	0.79	0.83	0.79	0.83	0.85	<36
CORE-OM1	0.93	0.93	0.94	0.93	0.94	0.95	>35
CORE-OM-NR1	0.93	0.93	0.94	0.92	0.94	0.94	>35
CORE-SFA1	0.89	0.89	0.90	0.88	0.91	0.91	>35
CORE-SFB1	0.89	0.88	0.90	0.87	0.90	0.90	>35
CORE-GP1	0.86	0.84	0.87	0.84	0.87	0.88	>35
CORE-101	0.82	0.80	0.83	0.79	0.84	0.85	>35

Plot of Cronbach’s alpha and 95%CI by form at T1 by age groups

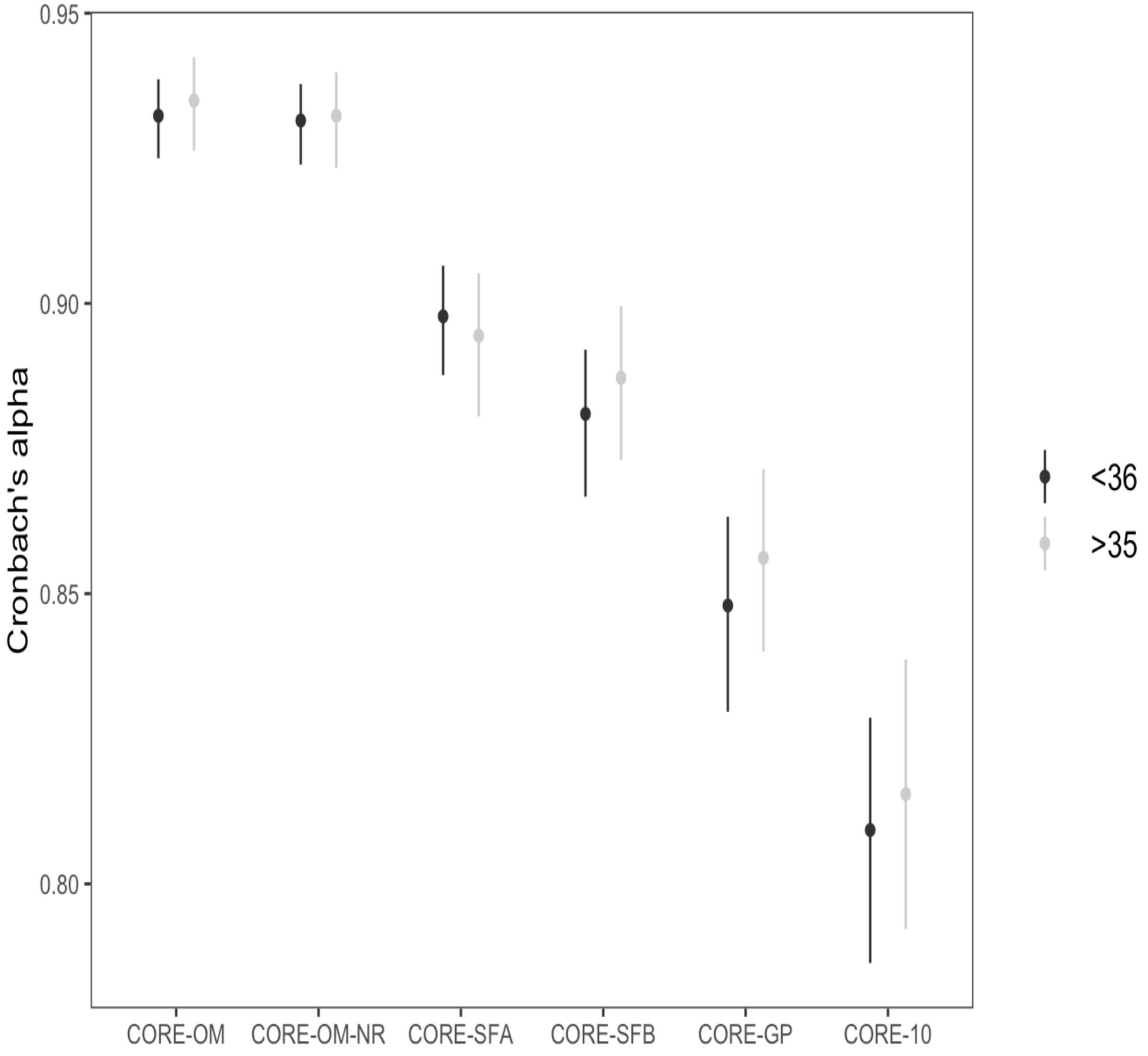

Reliability by level of education

Table of internal consistency coefficients by form at T1 and by education (higher education vs less than 12 years of education)

	alpha	LCI	UCI	bLCI	bUCI	omega	education
CORE-OM	0.94	0.93	0.94	0.93	0.94	0.95	12 or less
CORE-OM-NR	0.94	0.93	0.94	0.93	0.94	0.94	12 or less
CORE-SFA	0.90	0.89	0.91	0.89	0.91	0.91	12 or less
CORE-SFB	0.89	0.88	0.90	0.88	0.90	0.90	12 or less

	alpha	LCI	UCI	bLCI	bUCI	omega	education
CORE-GP	0.86	0.85	0.87	0.84	0.87	0.89	12 or less
CORE-10	0.82	0.80	0.84	0.80	0.84	0.85	12 or less
CORE-OM1	0.93	0.92	0.93	0.92	0.94	0.94	higher_education
CORE-OM-NR1	0.93	0.92	0.93	0.92	0.93	0.94	higher_education
CORE-SFA1	0.89	0.88	0.90	0.88	0.90	0.91	higher_education
CORE-SFB1	0.87	0.86	0.88	0.86	0.89	0.89	higher_education
CORE-GP1	0.84	0.83	0.85	0.82	0.86	0.88	higher_education
CORE-101	0.80	0.78	0.82	0.77	0.82	0.83	higher_education

Plot of Cronbach's alpha and 95%CI by form at T1 by education

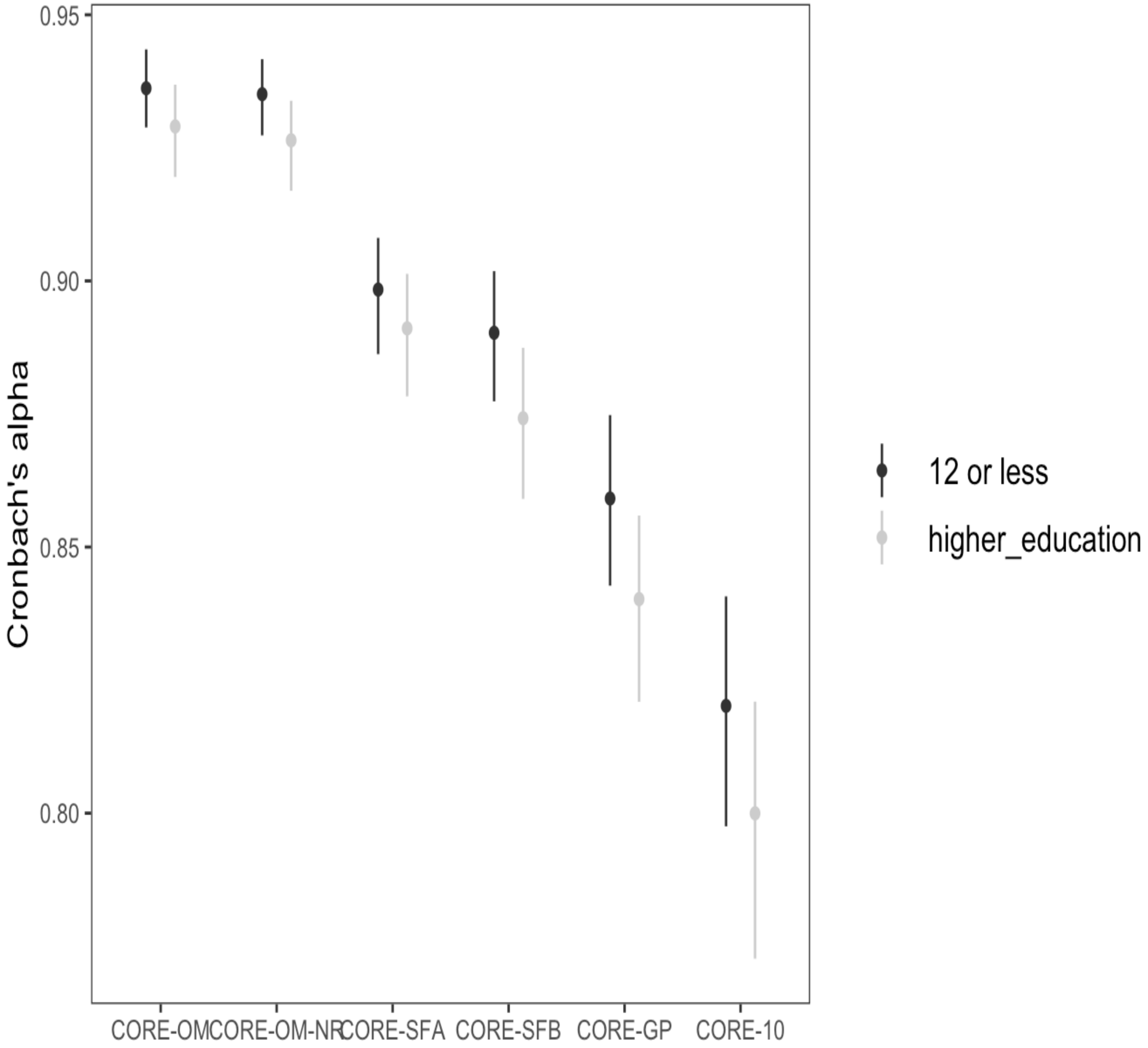

Reliability by tranche

Table of internal consistency coefficients by form at T1 and by tranche

	alpha	LCI	UCI	bLCI	bUCI	omega	wave
CORE-OM	0.92	0.89	0.94	0.87	0.95	0.94	2010-2011
CORE-OM-NR	0.92	0.89	0.94	0.86	0.94	0.94	2010-2011
CORE-SFA	0.89	0.86	0.92	0.84	0.92	0.92	2010-2011
CORE-SFB	0.84	0.80	0.89	0.76	0.89	0.88	2010-2011
CORE-GP	0.84	0.79	0.88	0.75	0.89	0.85	2010-2011
CORE-10	0.76	0.68	0.82	0.60	0.84	0.84	2010-2011
CORE-OM1	0.92	0.90	0.94	0.89	0.94	0.94	2011-2012
CORE-OM-NR1	0.92	0.90	0.94	0.89	0.94	0.94	2011-2012
CORE-SFA1	0.88	0.84	0.91	0.83	0.91	0.90	2011-2012
CORE-SFB1	0.87	0.83	0.90	0.81	0.90	0.90	2011-2012
CORE-GP1	0.85	0.81	0.89	0.80	0.89	0.90	2011-2012
CORE-101	0.79	0.72	0.84	0.70	0.85	0.84	2011-2012
CORE-OM2	0.93	0.92	0.94	0.92	0.94	0.94	2012-2013
CORE-OM-NR2	0.93	0.92	0.93	0.91	0.94	0.94	2012-2013
CORE-SFA2	0.89	0.87	0.90	0.87	0.90	0.91	2012-2013
CORE-SFB2	0.88	0.86	0.89	0.86	0.90	0.90	2012-2013
CORE-GP2	0.85	0.83	0.86	0.82	0.87	0.88	2012-2013
CORE-102	0.79	0.76	0.81	0.75	0.82	0.82	2012-2013
CORE-OM3	0.93	0.93	0.94	0.92	0.94	0.95	2013-2014
CORE-OM-NR3	0.93	0.92	0.94	0.92	0.94	0.94	2013-2014
CORE-SFA3	0.90	0.88	0.91	0.88	0.91	0.91	2013-2014
CORE-SFB3	0.89	0.87	0.90	0.86	0.90	0.90	2013-2014
CORE-GP3	0.85	0.83	0.87	0.82	0.87	0.88	2013-2014
CORE-103	0.82	0.80	0.84	0.78	0.85	0.87	2013-2014
CORE-OM4	0.94	0.93	0.95	0.93	0.95	0.95	2014-2015
CORE-OM-NR4	0.94	0.93	0.95	0.93	0.95	0.95	2014-2015
CORE-SFA4	0.90	0.89	0.91	0.88	0.92	0.92	2014-2015
CORE-SFB4	0.90	0.88	0.91	0.87	0.91	0.91	2014-2015
CORE-GP4	0.86	0.84	0.88	0.84	0.89	0.89	2014-2015
CORE-104	0.83	0.80	0.85	0.79	0.85	0.85	2014-2015
CORE-OM5	0.93	0.93	0.94	0.92	0.94	0.95	2017-2018
CORE-OM-NR5	0.93	0.92	0.94	0.92	0.94	0.94	2017-2018
CORE-SFA5	0.90	0.89	0.91	0.89	0.91	0.92	2017-2018
CORE-SFB5	0.88	0.87	0.90	0.86	0.90	0.90	2017-2018
CORE-GP5	0.85	0.83	0.87	0.82	0.87	0.88	2017-2018
CORE-105	0.82	0.79	0.84	0.78	0.84	0.85	2017-2018

Plot of Cronbach's alpha and 95%CI by form at T1 by tranche

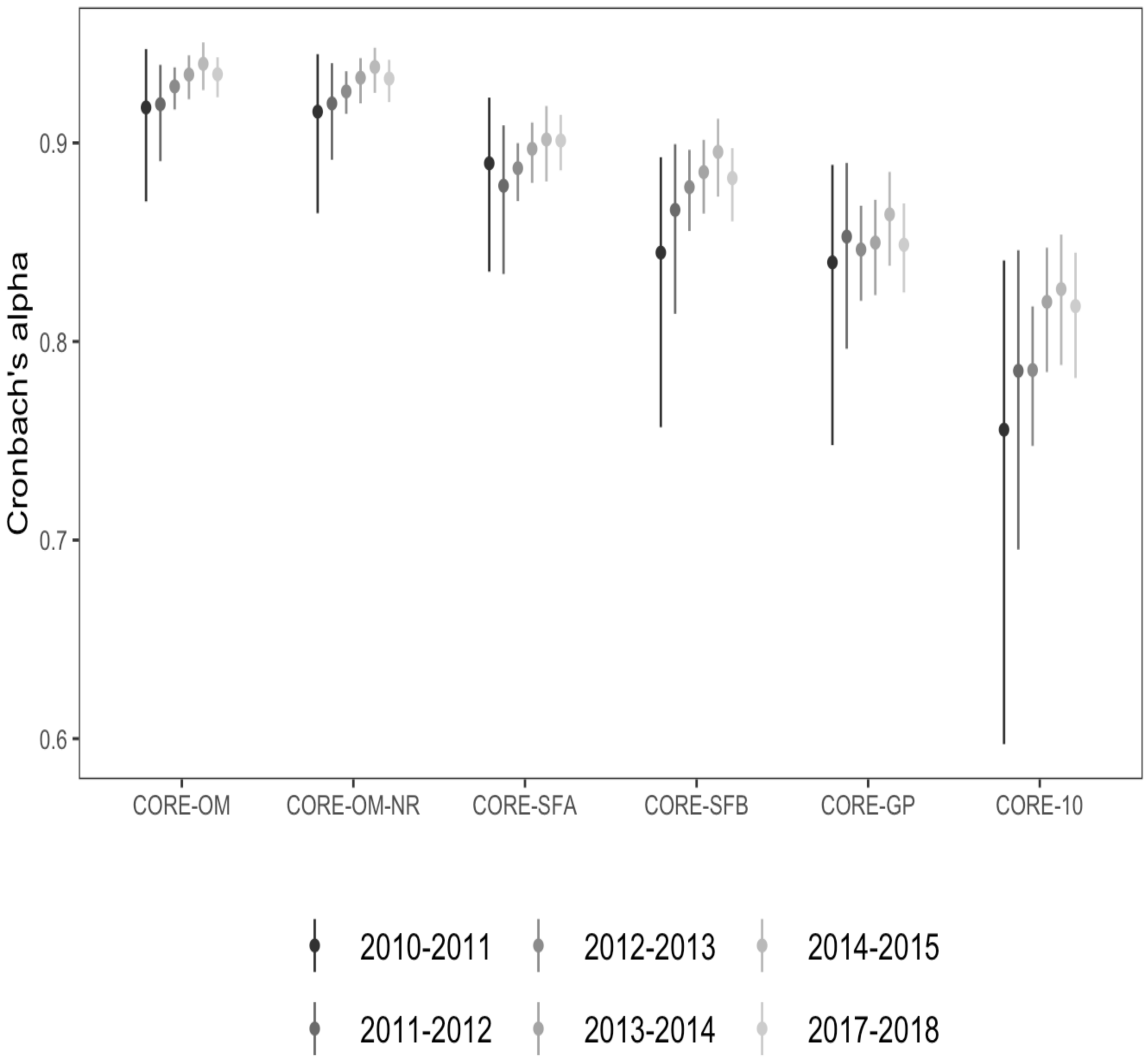

Test-Retest Reliability

Correlation total (any T2)

	obsCorr	LCLCorr	UCLCorr
CORE-OM	0.76	0.64	0.84
CORE-OM-NR	0.75	0.64	0.83
CORE-SFA	0.72	0.61	0.82
CORE-SFB	0.75	0.65	0.83

	obsCorr	LCLCorr	UCLCorr
CORE-GP	0.74	0.64	0.81
CORE-10	0.70	0.56	0.81

Correlation one week

	obsCorr	LCLCorr	UCLCorr
CORE-OM	0.81	0.69	0.89
CORE-OM-NR	0.79	0.67	0.87
CORE-SFA	0.78	0.65	0.87
CORE-SFB	0.79	0.68	0.87
CORE-GP	0.77	0.66	0.85
CORE-10	0.72	0.53	0.84

Correlation six months

	obsCorr	LCLCorr	UCLCorr
CORE-OM	0.62	0.43	0.77
CORE-OM-NR	0.63	0.42	0.77
CORE-SFA	0.59	0.37	0.77
CORE-SFB	0.61	0.39	0.76
CORE-GP	0.65	0.46	0.80
CORE-10	0.64	0.48	0.79

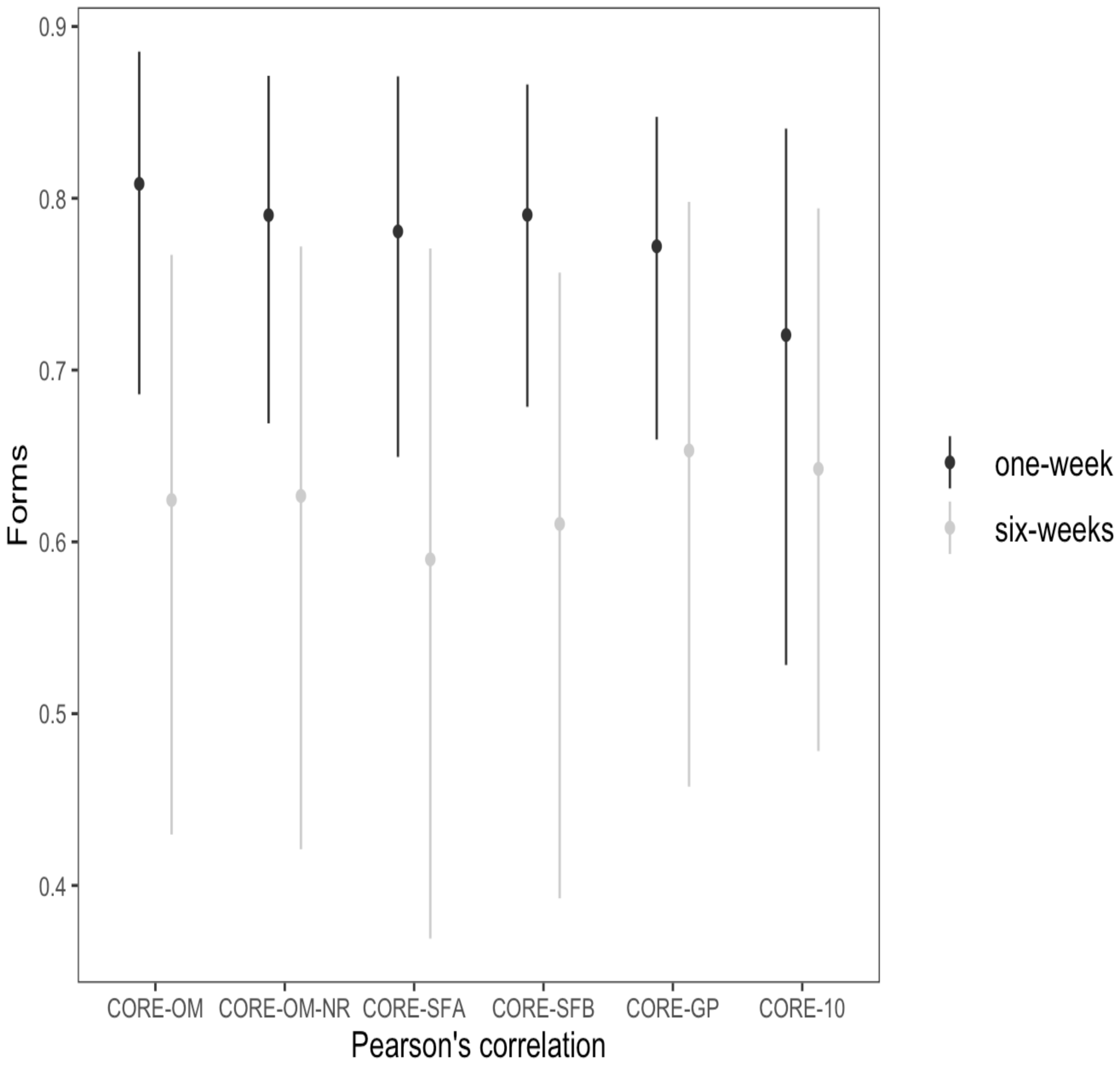
0.9
0.8
0.7
0.6
0.5
0.4
Forms
CORE-OM
CORE-OM-NR
CORE-SFA
CORE-SFB
CORE-GP
CORE-10
Pearson's correlation
one-week
six-weeks

Mean Difference

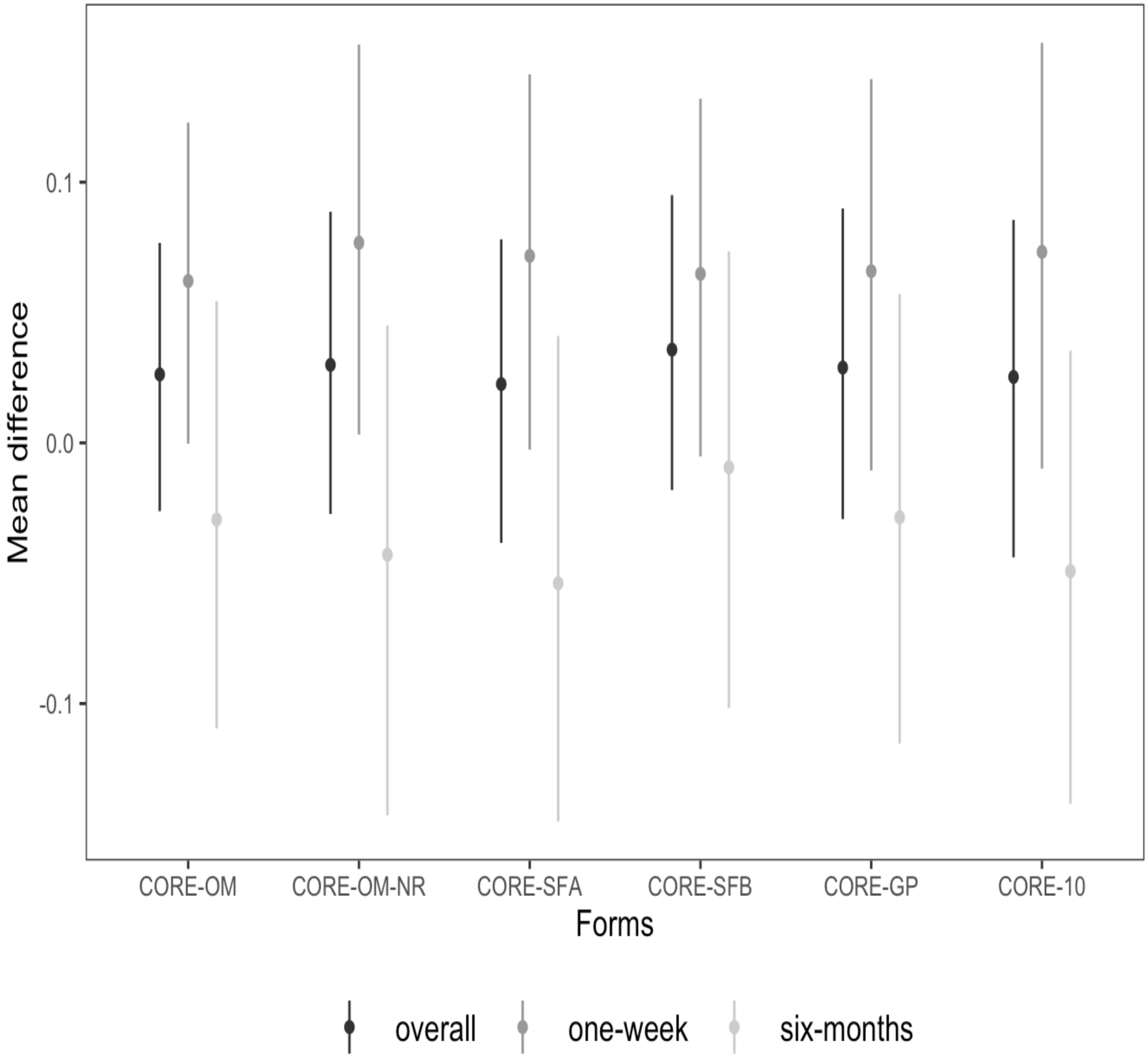

Augmented scattergrams

That plot packs quite a lot of information in but it tells us a lot about the data and the test-retest reliability. The title shows the rates of missing data. The marginal histograms show the distribution of the scores on the two occasions. The points use shading to show overprinting of more than one participant with exactly the same scores on the two occasions. The red regression line is the best fit linear regression and the blue wavy line is the local smoothed regression with its

95% confidence interval (CI) around that in cyan. The means are indicated by the dotted lines, the vertical line for the t1 data, the horizontal for the t2. The black points and error bars labelled “m1” and “m2” mark the means and their 95% CIs for occasion 1 and 2 so the t1 mean and CI are on the left and the t2 at the bottom. That enables us to see how they relate to the other mean. The annotation in the top left tells us the Pearson R correlation coefficient and the equation of the best fitting linear regression.

CORE-OM

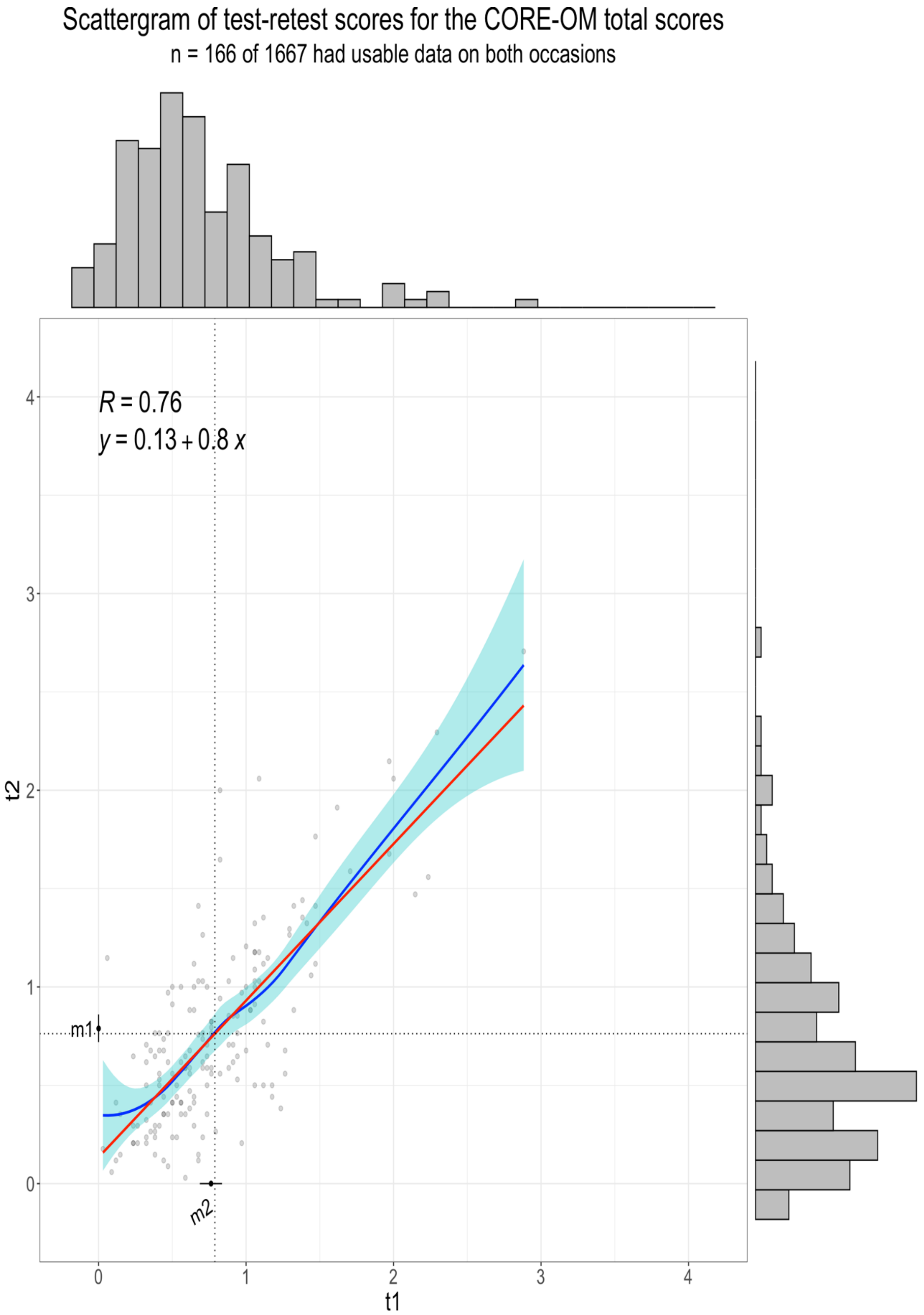

For the CORE-OM the test-retest data is from a small subset of the full set of participants.The marginal histograms show, as you'd expect for this non-help-seeking dataset, that there is a

strong right skew in the data and the maximum observed scores on either occasion are well short of the maximum possible score on the measure. The linear regression line lies entirely within the CI of the smoothed regression suggesting that there is no marked non-linear aspect to the mapping between scores on the two occasions. The fact that t1 CI cuts the t2 mean, and *vice versa* tells us that there was no marked mean shift between the two occasions and shows the precision of estimation of the shift. The Pearson correlation is high, the intercept of the linear regression is low, close to zero and the slope, at .82, is high.

CORE-OM-NR

Scattergram of test-retest scores for the CORE-OM/NR scores
n = 166 of 1667 had usable data on both occasions

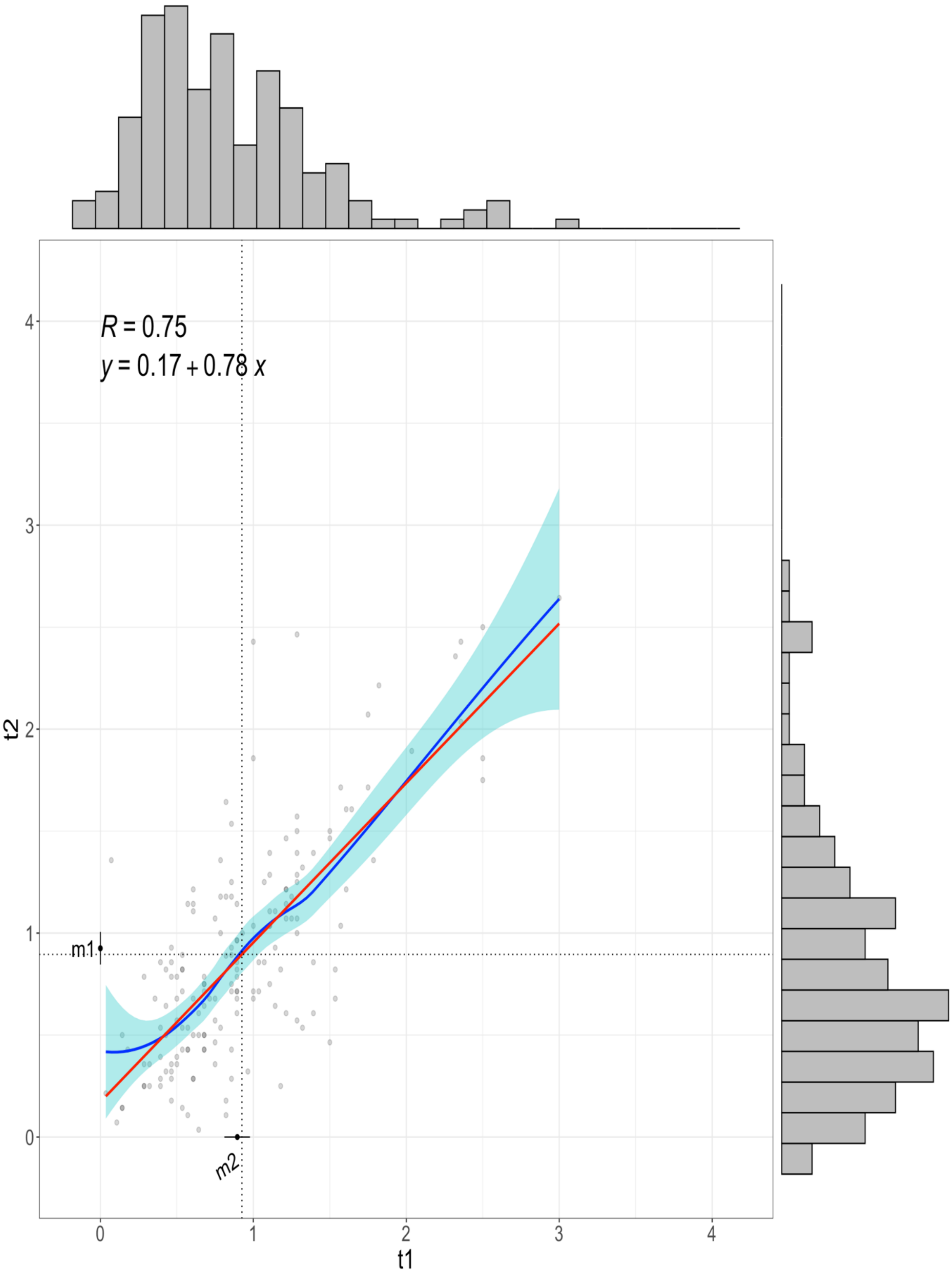

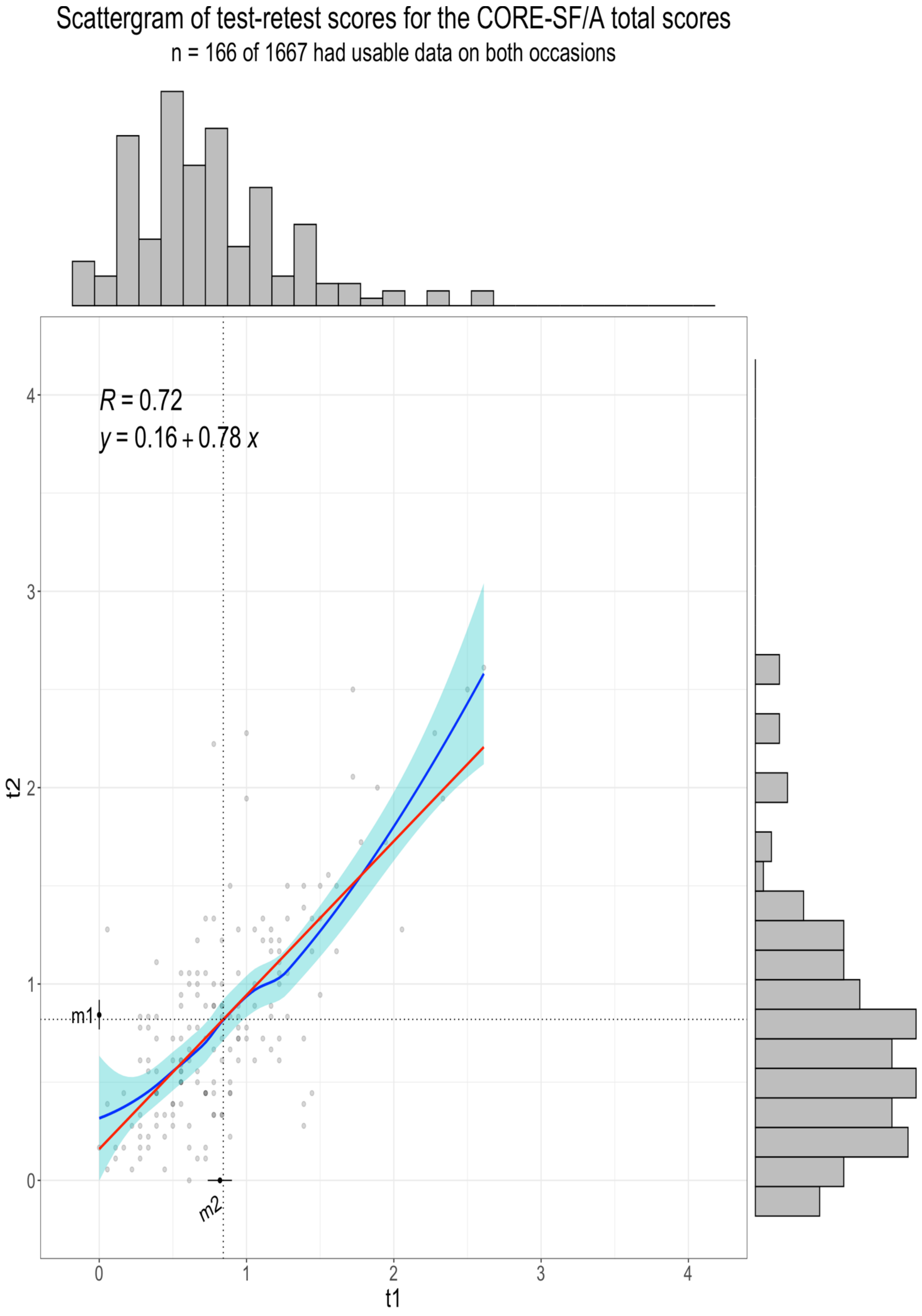

Scattergram of test-retest scores for the CORE-SF/A total scores
n = 166 of 1667 had usable data on both occasions
R = 0.72
y = 0.16 + 0.78 x
m1
m2
t1
t2
0
1
2
3
4

CORE-SFB

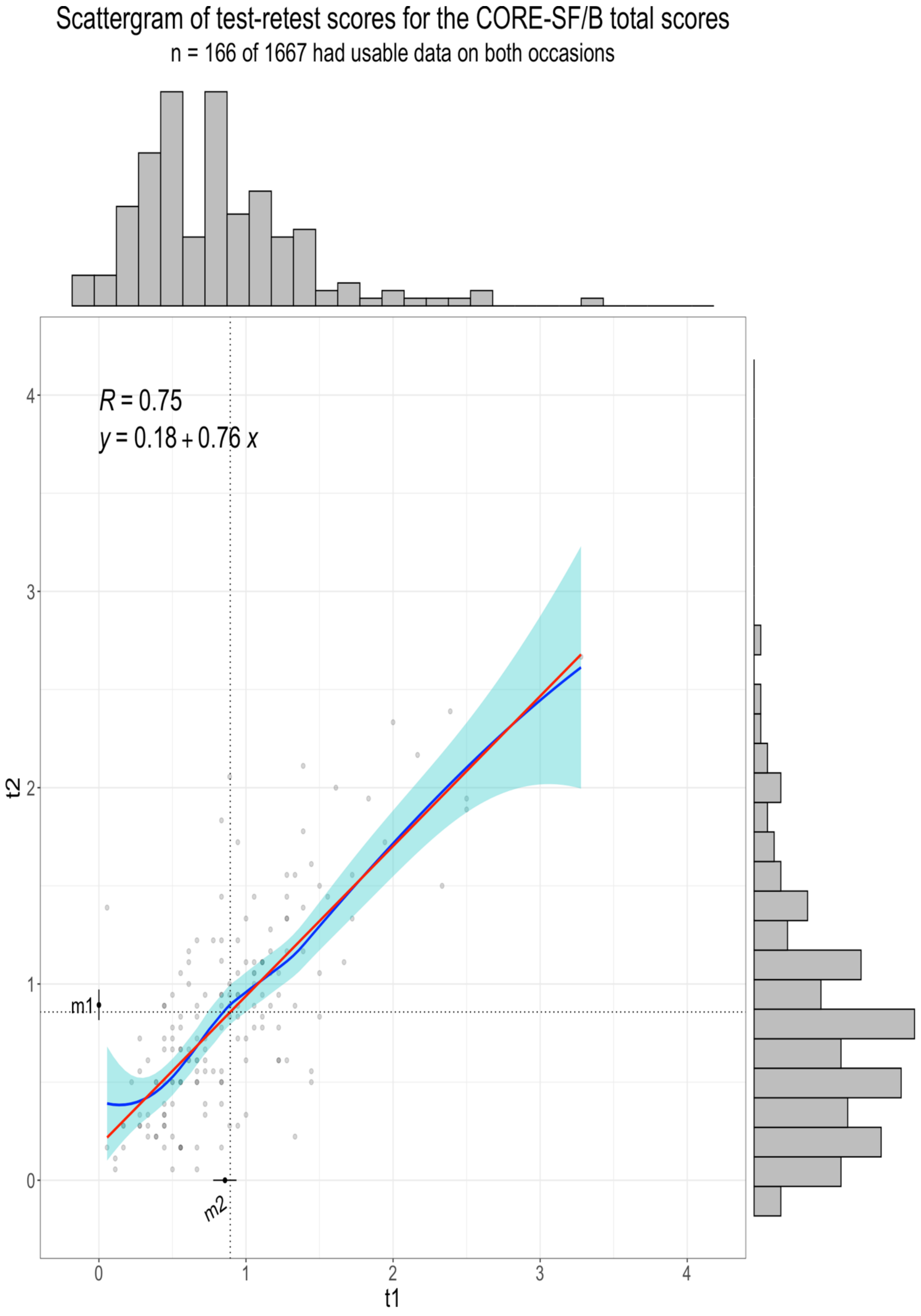

Scattergram of test-retest scores for the CORE-SF/B total scores
n = 166 of 1667 had usable data on both occasions
R = 0.75
y = 0.18 + 0.76 x
m1
m2
t1
t2
0
1
2
3
4

CORE-GP

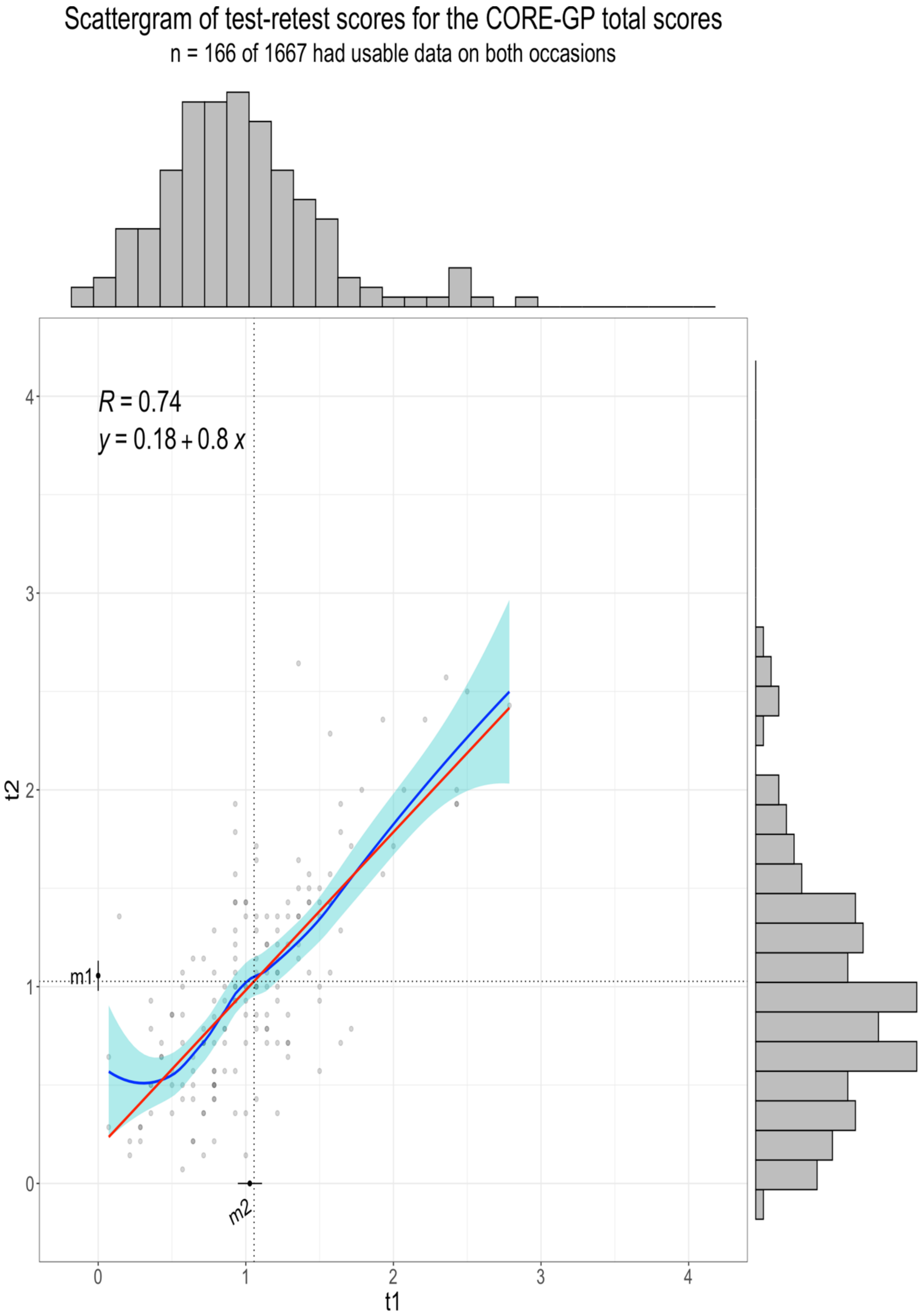

Scattergram of test-retest scores for the CORE-GP total scores
n = 166 of 1667 had usable data on both occasions
R = 0.74
y = 0.18 + 0.8 x
t2
t1
m1
m2
0
1
2
3
4
0
1
2
3
4

CORE-10

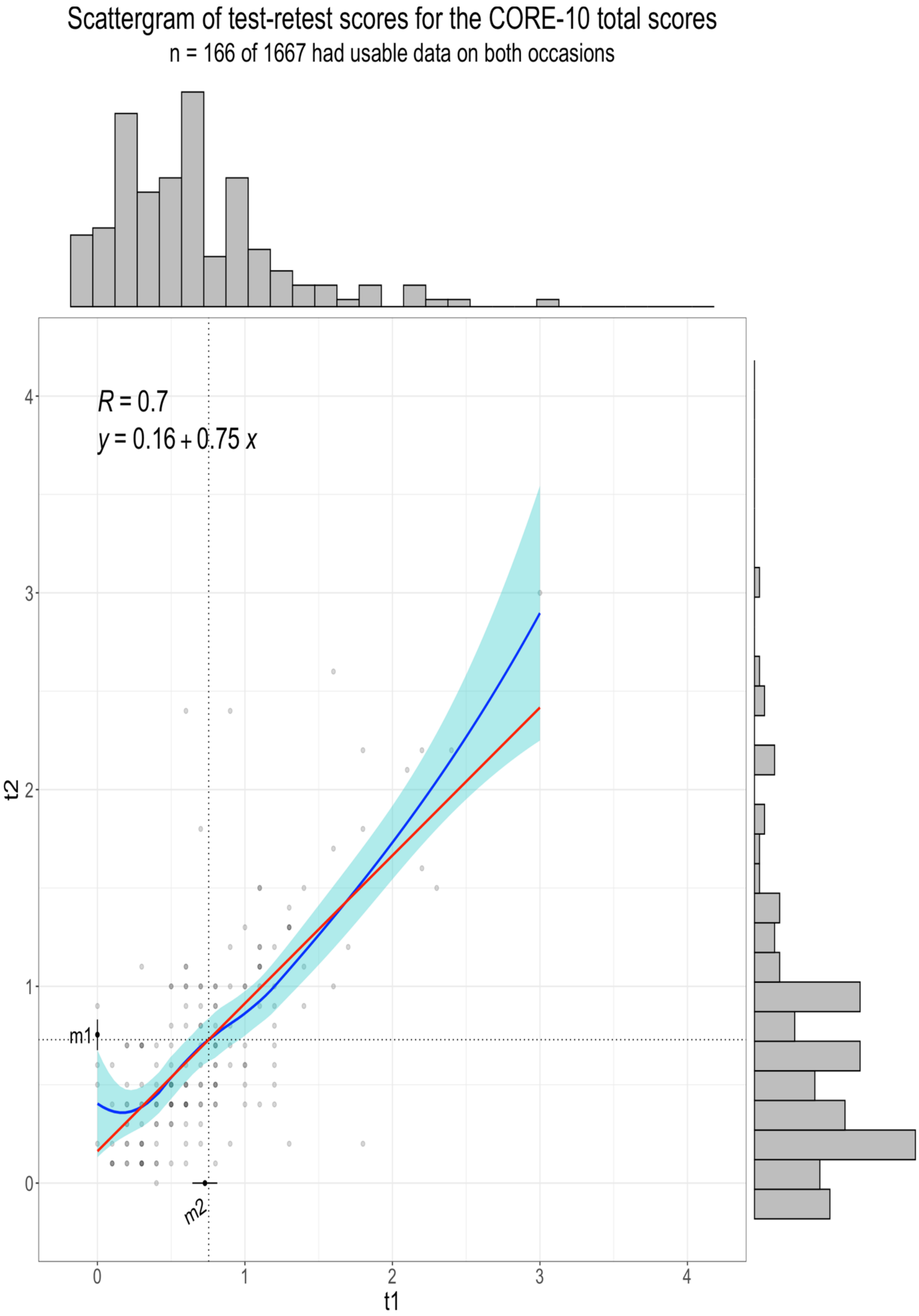
Scattergram of test-retest scores for the CORE-10 total scores
n = 166 of 1667 had usable data on both occasions
R = 0.7
y = 0.16 + 0.75 x
m1
m2
t1
t2
0
1
2
3
4

4. Description of the scores

Total

Description of the score by form

	n	min	max	sd	mean	LCL	UCL
CORE-OM	1666	0	3.06	0.51	0.8522679	0.82774	0.8760443
CORE-OM-NR	1666	0	3.36	0.58	1.003013	0.9734326	1.028273
CORE-SFA	1666	0	3.17	0.55	0.9081378	0.8820471	0.936575
CORE-SFB	1666	0	3.33	0.56	0.9590817	0.9324904	0.9856737
CORE-GP	1666	0	3.14	0.57	1.103311	1.076048	1.130218
CORE-10	1666	0	3.10	0.56	0.8342737	0.8074438	0.8621106

By gender

Plot describing the scores by gender

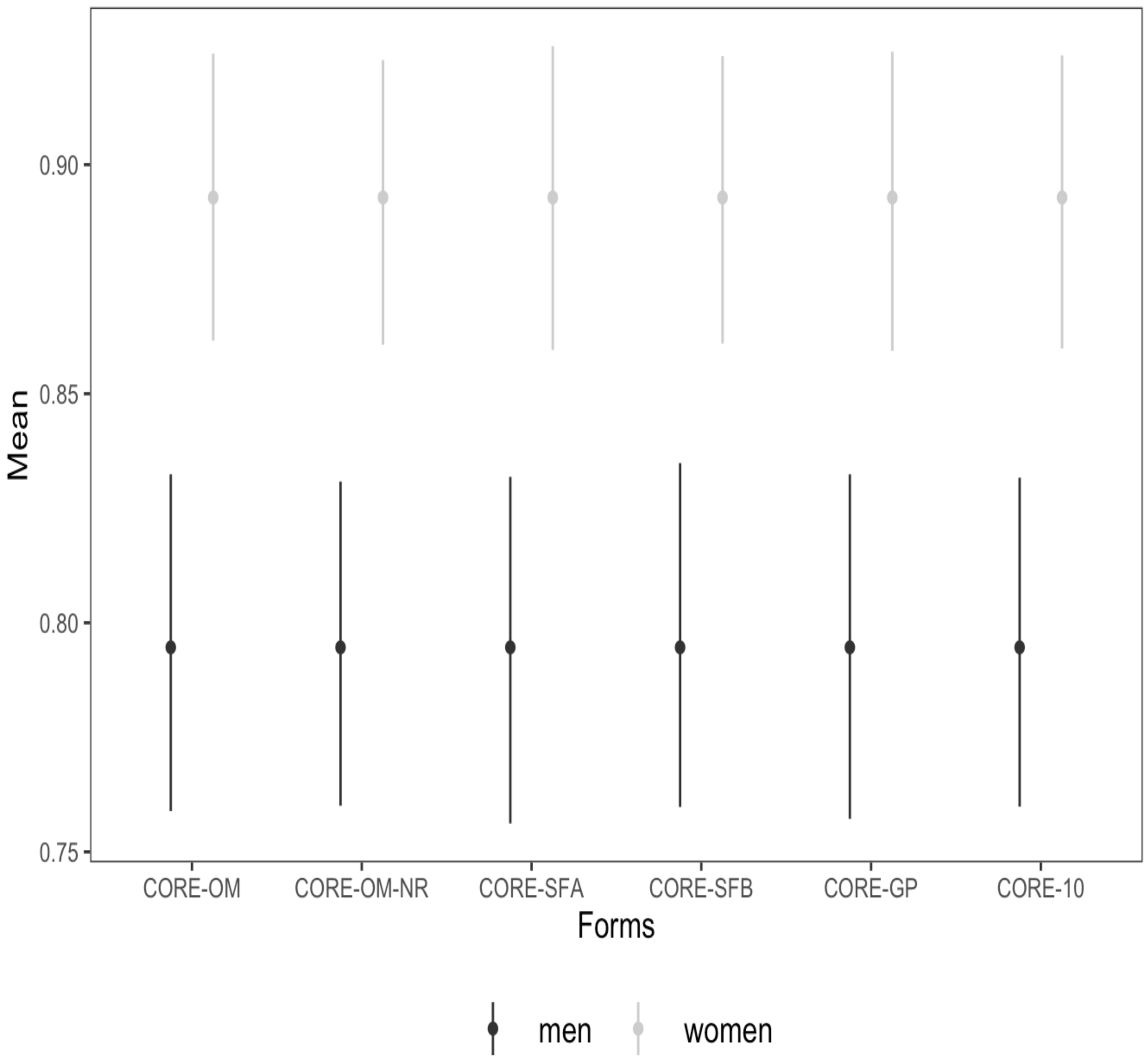

gender	n	min	max	sd	mean	LCL	UCL	form
men	681	0.03	3.06	0.49	0.79	0.76	0.83	CORE-OM
women	982	0.00	2.97	0.52	0.89	0.86	0.92	CORE-OM
men	681	0.04	3.36	0.55	0.79	0.76	0.83	CORE-OM-NR
women	982	0.00	3.11	0.59	0.89	0.86	0.92	CORE-OM-NR
men	681	0.00	2.89	0.52	0.79	0.76	0.83	CORE-SFA
women	982	0.00	3.17	0.56	0.89	0.86	0.93	CORE-SFA
men	681	0.00	3.33	0.53	0.79	0.76	0.83	CORE-SFB
women	982	0.00	2.94	0.57	0.89	0.86	0.92	CORE-SFB
men	681	0.00	3.14	0.55	0.79	0.76	0.83	CORE-GP
women	982	0.00	3.14	0.58	0.89	0.86	0.92	CORE-GP
men	681	0.00	3.10	0.53	0.79	0.76	0.83	CORE-10

gender	n	min	max	sd	mean	LCL	UCL	form
women	982	0.00	3.10	0.57	0.89	0.86	0.92	CORE-10

Table showing differences among gender

form	Diff	LCLdiff	UCLdiff
CORE-OM	0.10	0.05	0.15
CORE-OM-NR	0.12	0.07	0.18
CORE-SFA	0.13	0.07	0.18
CORE-SFB	0.11	0.06	0.16
CORE-GP	0.08	0.03	0.14
CORE-10	0.12	0.06	0.17

By age

Plot describing scores among age groups

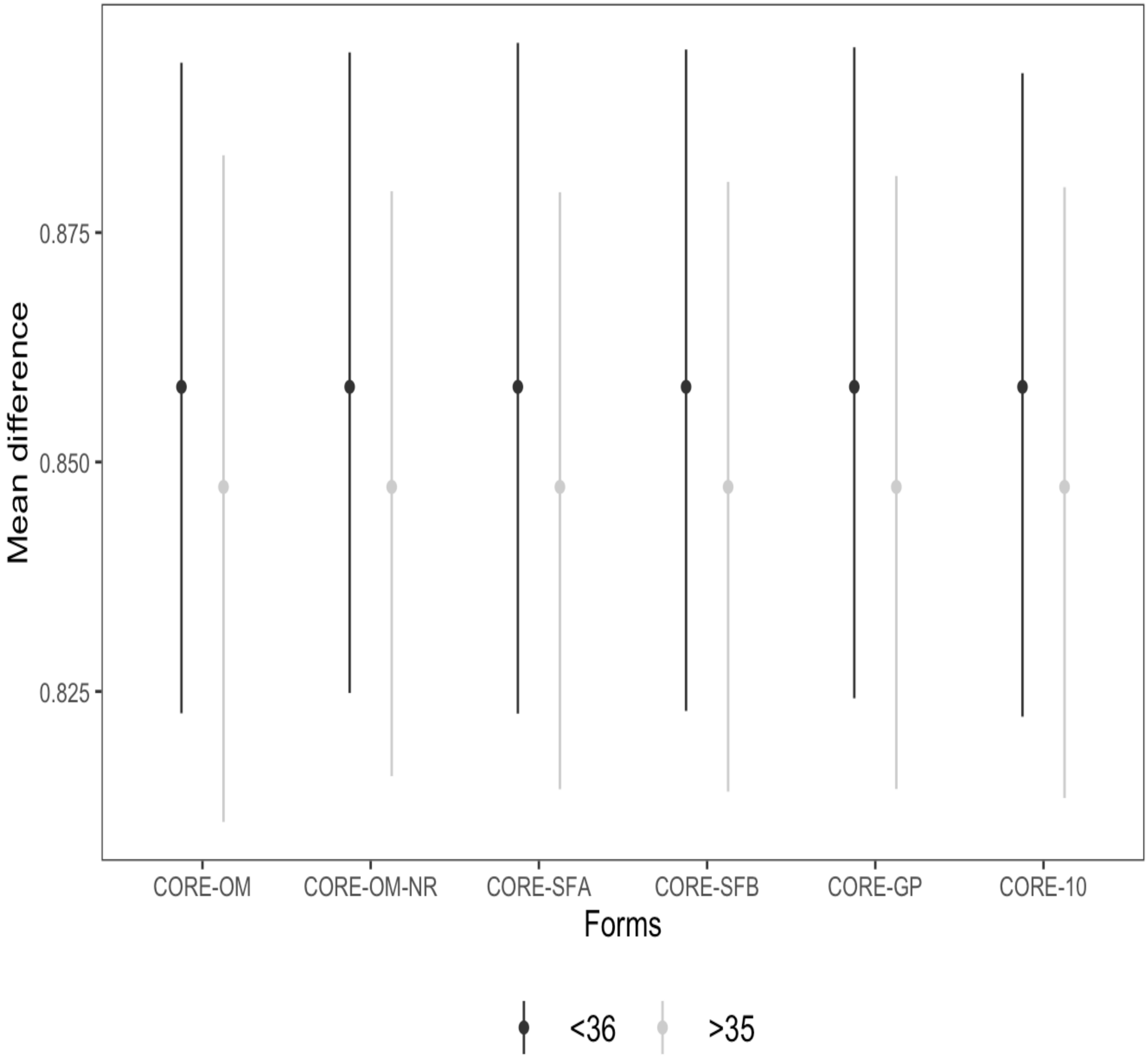

**age2**	**n**	**min**	**max**	**sd**	**mean**	**LCL**	**UCL**	**form**
<36	814	0	2.65	0.51	0.86	0.82	0.89	CORE-OM
>35	849	0	3.06	0.51	0.85	0.81	0.88	CORE-OM
<36	814	0	3.00	0.58	0.86	0.82	0.89	CORE-OM-NR
>35	849	0	3.36	0.57	0.85	0.82	0.88	CORE-OM-NR
<36	814	0	2.83	0.56	0.86	0.82	0.90	CORE-SFA
>35	849	0	3.17	0.54	0.85	0.81	0.88	CORE-SFA
<36	814	0	2.94	0.56	0.86	0.82	0.89	CORE-SFB
>35	849	0	3.33	0.56	0.85	0.81	0.88	CORE-SFB
<36	814	0	3.07	0.56	0.86	0.82	0.90	CORE-GP
>35	849	0	3.14	0.57	0.85	0.81	0.88	CORE-GP

age2	n	min	max	sd	mean	LCL	UCL	form
<36	814	0	3.10	0.56	0.86	0.82	0.89	CORE-10
>35	849	0	3.10	0.56	0.85	0.81	0.88	CORE-10

Table showing differences among age groups

form	Diff	LCLdiff	UCLdiff
CORE-OM	-0.01	-0.06	0.04
CORE-OM-NR	-0.02	-0.07	0.04
CORE-SFA	-0.02	-0.07	0.04
CORE-SFB	-0.02	-0.07	0.03
CORE-GP	0.01	-0.04	0.07
CORE-10	-0.01	-0.06	0.05

By level of education

Plot describing scores among participants with differen levels of education

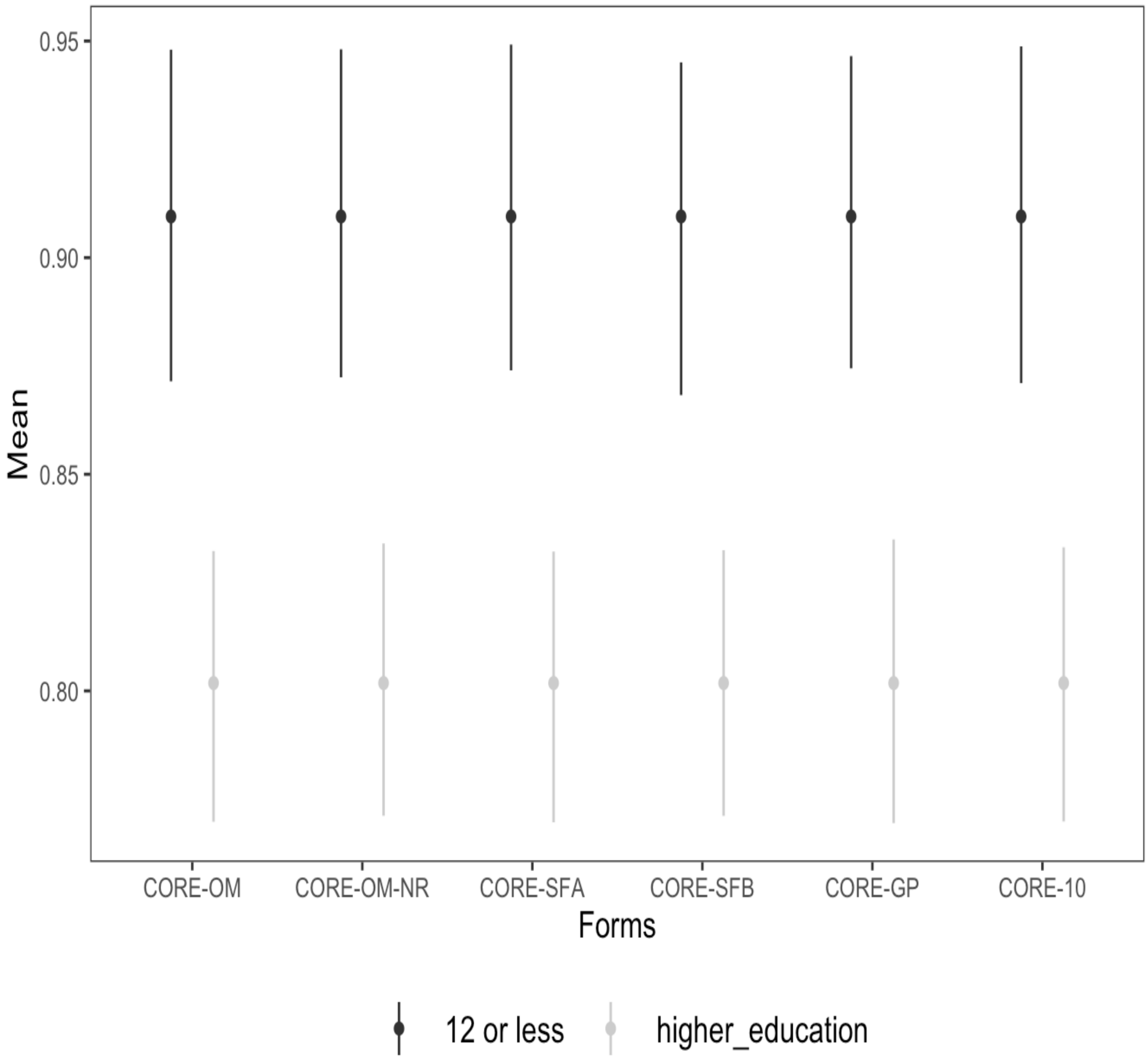

Table showing differences among participants with different levels of education

**form**	**Diff**	**LCLdiff**	**UCLdiff**
CORE-OM	-0.11	-0.16	-0.06
CORE-OM-NR	-0.12	-0.18	-0.07
CORE-SFA	-0.11	-0.16	-0.06
CORE-SFB	-0.12	-0.17	-0.06
CORE-GP	-0.11	-0.17	-0.05
CORE-10	-0.11	-0.17	-0.06

Distributions

By Gender

CORE-OM

Table of the distribution by gender

prob	n	nOK	nMiss	quantile	LCL	UCL	form	gender
0.05	681	681	0	0.1764706	0.1176471	0.2352941	CORE-OM	men
0.10	681	681	0	0.2647059	0.2352941	0.2941176	CORE-OM	men
0.15	681	681	0	0.3235294	0.2941176	0.3823529	CORE-OM	men
0.20	681	681	0	0.3823529	0.3529412	0.4411765	CORE-OM	men
0.30	681	681	0	0.5000000	0.4705882	0.5588235	CORE-OM	men
0.40	681	681	0	0.6176471	0.5588235	0.6470588	CORE-OM	men
0.50	681	681	0	0.7058824	0.6764706	0.7352941	CORE-OM	men
0.60	681	681	0	0.7941176	0.7647059	0.8529412	CORE-OM	men
0.70	681	681	0	0.9705882	0.9117647	1.0000000	CORE-OM	men
0.80	681	681	0	1.1176471	1.0588235	1.1764706	CORE-OM	men
0.90	681	681	0	1.4705882	1.3446659	1.5882353	CORE-OM	men
0.95	681	681	0	1.7941176	1.7033694	1.9117647	CORE-OM	men
0.05	982	982	0	0.2058824	0.1764706	0.2352941	CORE-OM	women
0.10	982	982	0	0.2941176	0.2647059	0.3235294	CORE-OM	women
0.15	982	982	0	0.3823529	0.3529412	0.4117647	CORE-OM	women
0.20	982	982	0	0.4352941	0.4117647	0.4705882	CORE-OM	women
0.30	982	982	0	0.5588235	0.5294118	0.6176471	CORE-OM	women
0.40	982	982	0	0.6764706	0.6470588	0.7058824	CORE-OM	women
0.50	982	982	0	0.8235294	0.7647059	0.8529412	CORE-OM	women
0.60	982	982	0	0.9411765	0.9117647	1.0000000	CORE-OM	women
0.70	982	982	0	1.1176471	1.0882353	1.1764706	CORE-OM	women
0.80	982	982	0	1.3000000	1.2647059	1.3529412	CORE-OM	women
0.90	982	982	0	1.5882353	1.5000000	1.6764706	CORE-OM	women
0.95	982	982	0	1.8823529	1.7834981	2.0000000	CORE-OM	women

Plot of the distributions by gender (black = men, red = women), quantiles= .05, .1, .15, .2, .3, .4, .5, .6, .7, .8, .9, .95

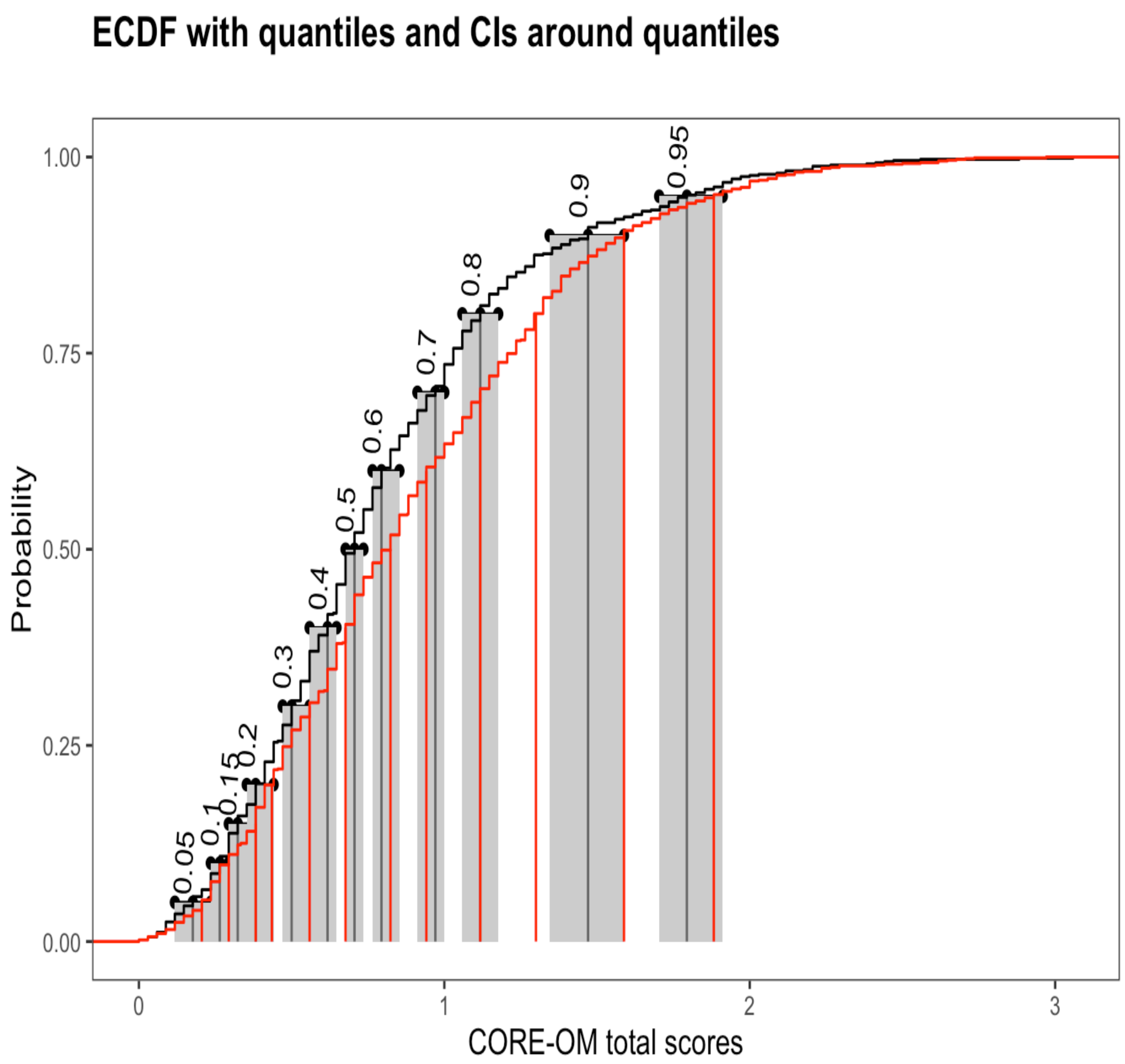

CORE-OM-NR

Table of the distribution by gender

prob	n	nOK	nMiss	quantile	LCL	UCL	form	gender
0.05	681	681	0	0.2142857	0.1428571	0.2500000	CORE-OM-NR	men
0.10	681	681	0	0.3214286	0.2857143	0.3571429	CORE-OM-NR	men
0.15	681	681	0	0.3928571	0.3571429	0.4285714	CORE-OM-NR	men

prob	n	nOK	nMiss	quantile	LCL	UCL	form	gender
0.20	681	681	0	0.4642857	0.4285714	0.5000000	CORE-OM-NR	men
0.30	681	681	0	0.6071429	0.5714286	0.6428571	CORE-OM-NR	men
0.40	681	681	0	0.7142857	0.6785714	0.7500000	CORE-OM-NR	men
0.50	681	681	0	0.8214286	0.7857143	0.8928571	CORE-OM-NR	men
0.60	681	681	0	0.9642857	0.9285714	1.0000000	CORE-OM-NR	men
0.70	681	681	0	1.1428571	1.0714286	1.2142857	CORE-OM-NR	men
0.80	681	681	0	1.3214286	1.2500000	1.3928571	CORE-OM-NR	men
0.90	681	681	0	1.6785714	1.5357143	1.7969413	CORE-OM-NR	men
0.95	681	681	0	2.0928571	1.9255199	2.2142857	CORE-OM-NR	men
0.05	982	982	0	0.2500000	0.2142857	0.2857143	CORE-OM-NR	women
0.10	982	982	0	0.3214286	0.3214286	0.3928571	CORE-OM-NR	women
0.15	982	982	0	0.4642857	0.4285714	0.4642857	CORE-OM-NR	women
0.20	982	982	0	0.5000000	0.4873747	0.5714286	CORE-OM-NR	women
0.30	982	982	0	0.6785714	0.6428571	0.7500000	CORE-OM-NR	women
0.40	982	982	0	0.8214286	0.7857143	0.8571429	CORE-OM-NR	women
0.50	982	982	0	0.9642857	0.8928571	1.0000000	CORE-OM-NR	women
0.60	982	982	0	1.1333333	1.0714286	1.2142857	CORE-OM-NR	women
0.70	982	982	0	1.3214286	1.2857143	1.3928571	CORE-OM-NR	women
0.80	982	982	0	1.5357143	1.5000000	1.6071429	CORE-OM-NR	women
0.90	982	982	0	1.8928571	1.7857143	1.9285714	CORE-OM-NR	women
0.95	982	982	0	2.1428571	2.0714286	2.2500000	CORE-OM-NR	women

Plot of the distributions by gender (black = men, red = women), quantiles = .05, .1, .15, .2, .3, .4, .5, .6, .7, .8, .9, .95

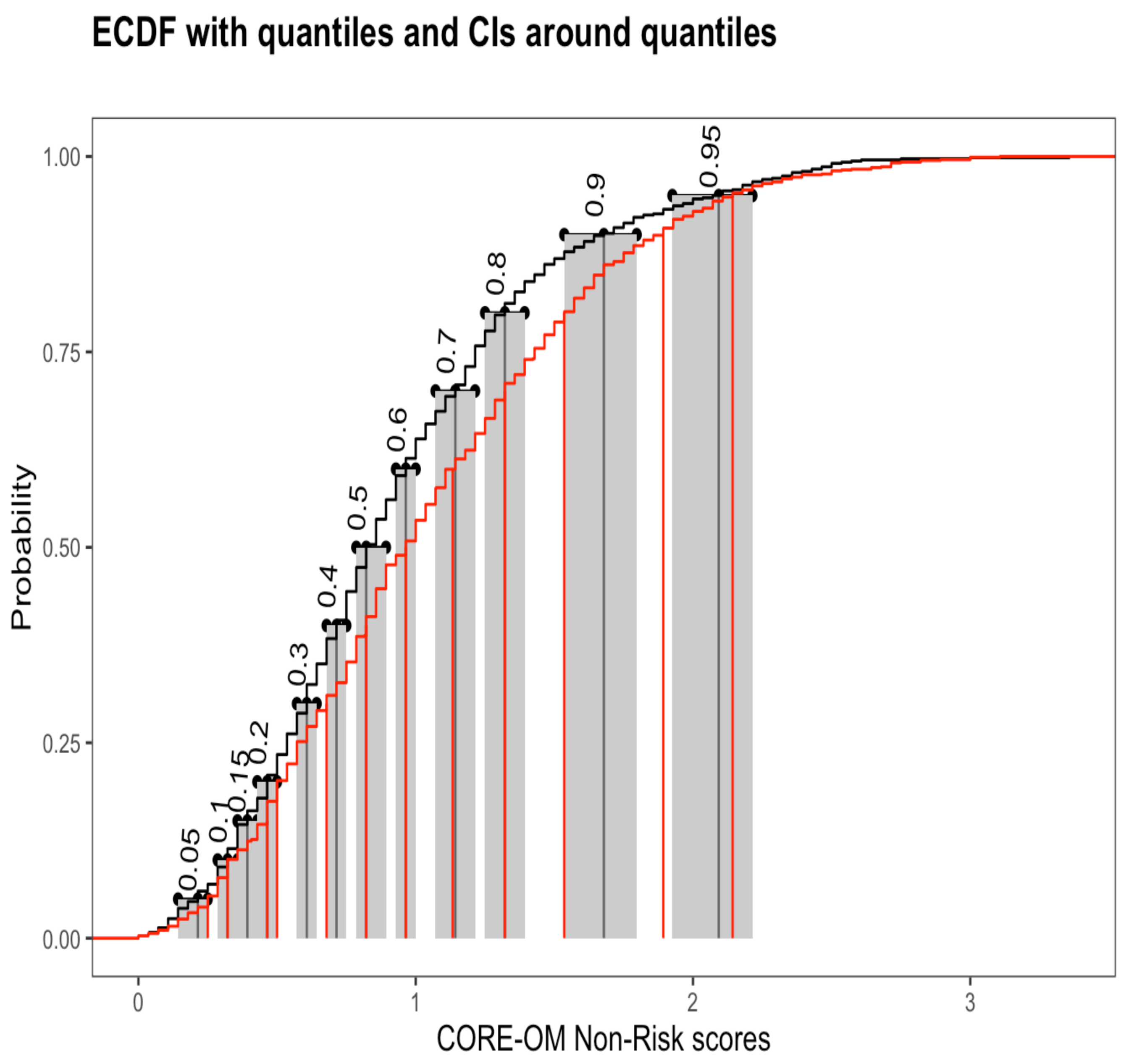

CORE-SFA

Table of the distribution by gender

prob	n	nOK	nMiss	quantile	LCL	UCL	form	gender
0.05	681	681	0	0.1666667	0.1111111	0.1666667	CORE-SFA	men
0.10	681	681	0	0.2777778	0.2222222	0.2777778	CORE-SFA	men
0.15	681	681	0	0.3333333	0.2777778	0.3888889	CORE-SFA	men
0.20	681	681	0	0.3888889	0.3333333	0.4444444	CORE-SFA	men
0.30	681	681	0	0.5000000	0.5000000	0.5555556	CORE-SFA	men

prob	n	nOK	nMiss	quantile	LCL	UCL	form	gender
0.40	681	681	0	0.6111111	0.5555556	0.6666667	CORE-SFA	men
0.50	681	681	0	0.7222222	0.7222222	0.7777778	CORE-SFA	men
0.60	681	681	0	0.8333333	0.8333333	0.8888889	CORE-SFA	men
0.70	681	681	0	1.0000000	0.9444444	1.0555556	CORE-SFA	men
0.80	681	681	0	1.1666667	1.1439458	1.2777778	CORE-SFA	men
0.90	681	681	0	1.5851852	1.4444444	1.6666667	CORE-SFA	men
0.95	681	681	0	1.9444444	1.7222222	2.0620810	CORE-SFA	men
0.05	982	982	0	0.2222222	0.1666667	0.2222222	CORE-SFA	women
0.10	982	982	0	0.3092593	0.2777778	0.3333333	CORE-SFA	women
0.15	982	982	0	0.3888889	0.3333333	0.4444444	CORE-SFA	women
0.20	982	982	0	0.4444444	0.4444444	0.5000000	CORE-SFA	women
0.30	982	982	0	0.6111111	0.5555556	0.6111111	CORE-SFA	women
0.40	982	982	0	0.7222222	0.6666667	0.7777778	CORE-SFA	women
0.50	982	982	0	0.8888889	0.8333333	0.9444444	CORE-SFA	women
0.60	982	982	0	1.0555556	1.0000000	1.1111111	CORE-SFA	women
0.70	982	982	0	1.2222222	1.1666667	1.2777778	CORE-SFA	women
0.80	982	982	0	1.4444444	1.3888889	1.5000000	CORE-SFA	women
0.90	982	982	0	1.7222222	1.6666667	1.7777778	CORE-SFA	women
0.95	982	982	0	1.9750000	1.8888889	2.0555556	CORE-SFA	women

Plot of the distributions by gender (black = men, red = women), quantiles= .05, .1, .15, .2, .3, .4, .5, .6, .7, .8, .9, .95

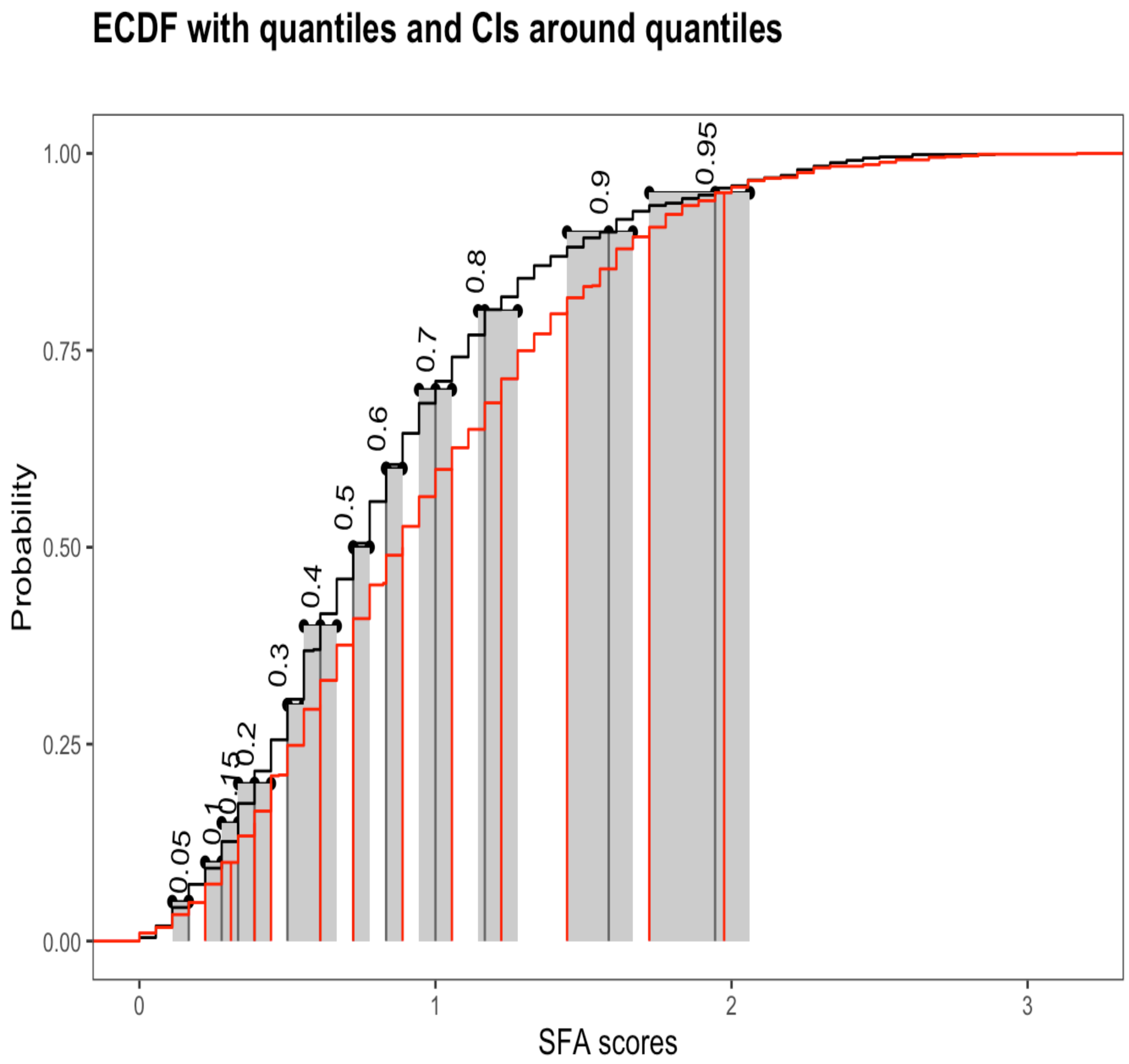

CORE-SFB

Table of the distribution by gender

prob	n	nOK	nMiss	quantile	LCL	UCL	form	gender
0.05	681	681	0	0.1666667	0.1111111	0.2222222	CORE-SFB	men
0.10	681	681	0	0.2777778	0.2603135	0.3333333	CORE-SFB	men
0.15	681	681	0	0.3888889	0.3333333	0.4444444	CORE-SFB	men
0.20	681	681	0	0.4444444	0.3888889	0.5000000	CORE-SFB	men
0.30	681	681	0	0.5555556	0.5555556	0.6111111	CORE-SFB	men

prob	n	nOK	nMiss	quantile	LCL	UCL	form	gender
0.40	681	681	0	0.7222222	0.6666667	0.7222222	CORE-SFB	men
0.50	681	681	0	0.7777778	0.7777778	0.8333333	CORE-SFB	men
0.60	681	681	0	0.9444444	0.8888889	1.0000000	CORE-SFB	men
0.70	681	681	0	1.0555556	1.0000000	1.1666667	CORE-SFB	men
0.80	681	681	0	1.2777778	1.2222222	1.3333333	CORE-SFB	men
0.90	681	681	0	1.5555556	1.4843690	1.7222222	CORE-SFB	men
0.95	681	681	0	1.9444444	1.7777778	2.1111111	CORE-SFB	men
0.05	982	982	0	0.2222222	0.1666667	0.2222222	CORE-SFB	women
0.10	982	982	0	0.3333333	0.2777778	0.3333333	CORE-SFB	women
0.15	982	982	0	0.3888889	0.3888889	0.4444444	CORE-SFB	women
0.20	982	982	0	0.5000000	0.4444444	0.5000000	CORE-SFB	women
0.30	982	982	0	0.6111111	0.5555556	0.6666667	CORE-SFB	women
0.40	982	982	0	0.8333333	0.7777778	0.8333333	CORE-SFB	women
0.50	982	982	0	0.9444444	0.8888889	0.9444444	CORE-SFB	women
0.60	982	982	0	1.1111111	1.0299732	1.1666667	CORE-SFB	women
0.70	982	982	0	1.2777778	1.2222222	1.3333333	CORE-SFB	women
0.80	982	982	0	1.4444444	1.3888889	1.5555556	CORE-SFB	women
0.90	982	982	0	1.7777778	1.7518950	1.8333333	CORE-SFB	women
0.95	982	982	0	2.0555556	1.9444444	2.1666667	CORE-SFB	women

Plot of the distributions by gender (black = men, red = women), quantiles= .05, .1, .15, .2, .3, .4, .5, .6, .7, .8, .9, .95

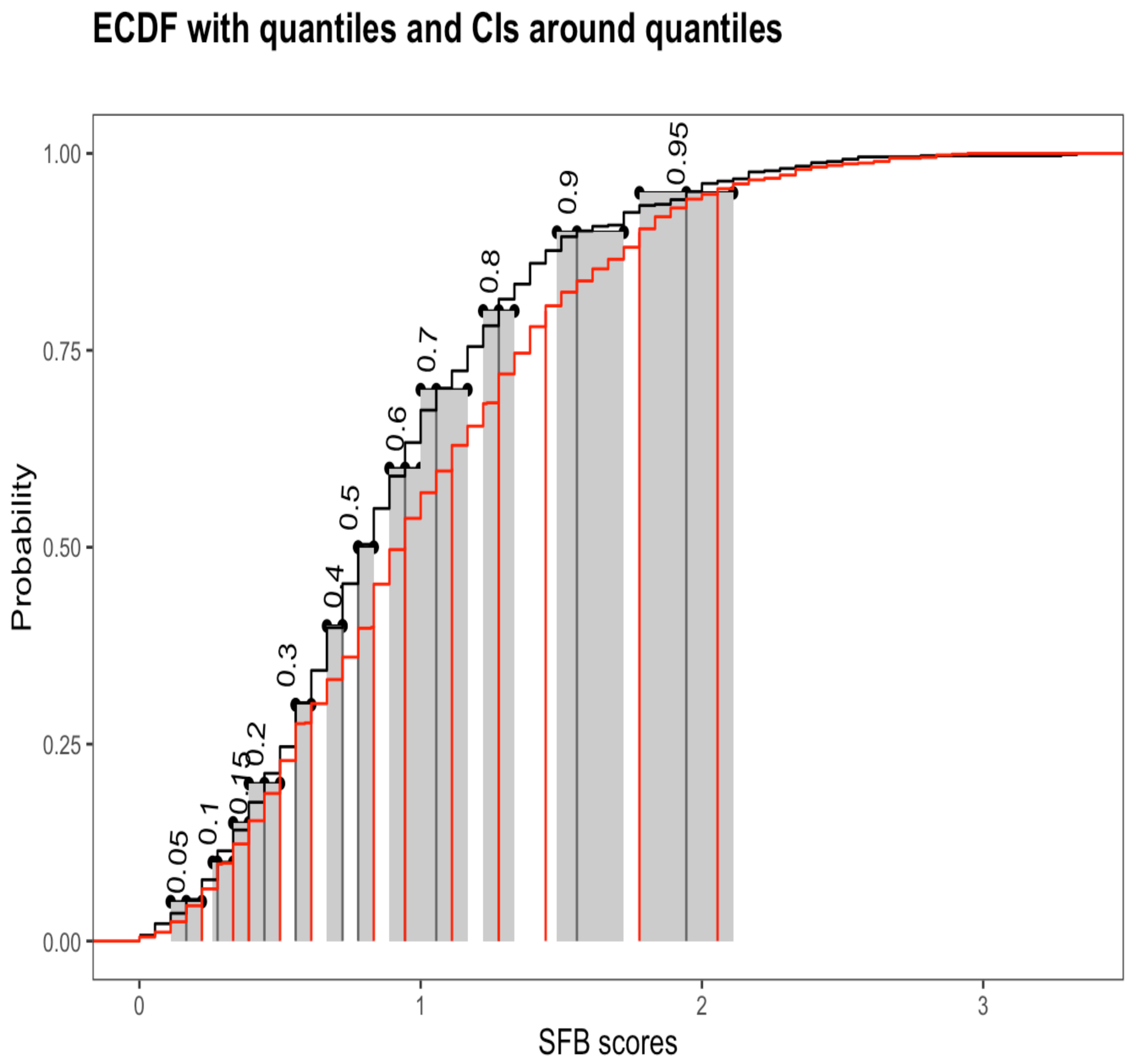

GP-CORE

Table of the distribution by gender

prob	n	nOK	nMiss	quantile	LCL	UCL	form	gender
0.05	681	681	0	0.2857143	0.2142857	0.2857143	GP-CORE	men
0.10	681	681	0	0.4285714	0.3571429	0.4285714	GP-CORE	men
0.15	681	681	0	0.5000000	0.4285714	0.5714286	GP-CORE	men
0.20	681	681	0	0.5714286	0.5714286	0.6428571	GP-CORE	men
0.30	681	681	0	0.7142857	0.7142857	0.7857143	GP-CORE	men

prob	n	nOK	nMiss	quantile	LCL	UCL	form	gender
0.40	681	681	0	0.8571429	0.8536452	0.9285714	GP-CORE	men
0.50	681	681	0	1.0000000	0.9285714	1.0714286	GP-CORE	men
0.60	681	681	0	1.1428571	1.0714286	1.1428571	GP-CORE	men
0.70	681	681	0	1.2857143	1.2142857	1.3155524	GP-CORE	men
0.80	681	681	0	1.4285714	1.3571429	1.5000000	GP-CORE	men
0.90	681	681	0	1.8571429	1.6428571	1.9285714	GP-CORE	men
0.95	681	681	0	2.0714286	2.0000000	2.2857143	GP-CORE	men
0.05	982	982	0	0.2857143	0.2142857	0.2857143	GP-CORE	women
0.10	982	982	0	0.4285714	0.3571429	0.5000000	GP-CORE	women
0.15	982	982	0	0.5714286	0.5000000	0.5714286	GP-CORE	women
0.20	982	982	0	0.6428571	0.6428571	0.7142857	GP-CORE	women
0.30	982	982	0	0.7857143	0.7142857	0.8150382	GP-CORE	women
0.40	982	982	0	0.9285714	0.8571429	1.0000000	GP-CORE	women
0.50	982	982	0	1.0714286	1.0000000	1.1428571	GP-CORE	women
0.60	982	982	0	1.2142857	1.1428571	1.2857143	GP-CORE	women
0.70	982	982	0	1.4285714	1.3571429	1.4285714	GP-CORE	women
0.80	982	982	0	1.6428571	1.5714286	1.6428571	GP-CORE	women
0.90	982	982	0	1.9285714	1.8571429	2.0000000	GP-CORE	women
0.95	982	982	0	2.2142857	2.0714286	2.2857143	GP-CORE	women

Plot of the distributions by gender (black = men, red = women), quantiles= .05, .1, .15, .2, .3, .4, .5, .6, .7, .8, .9, .95

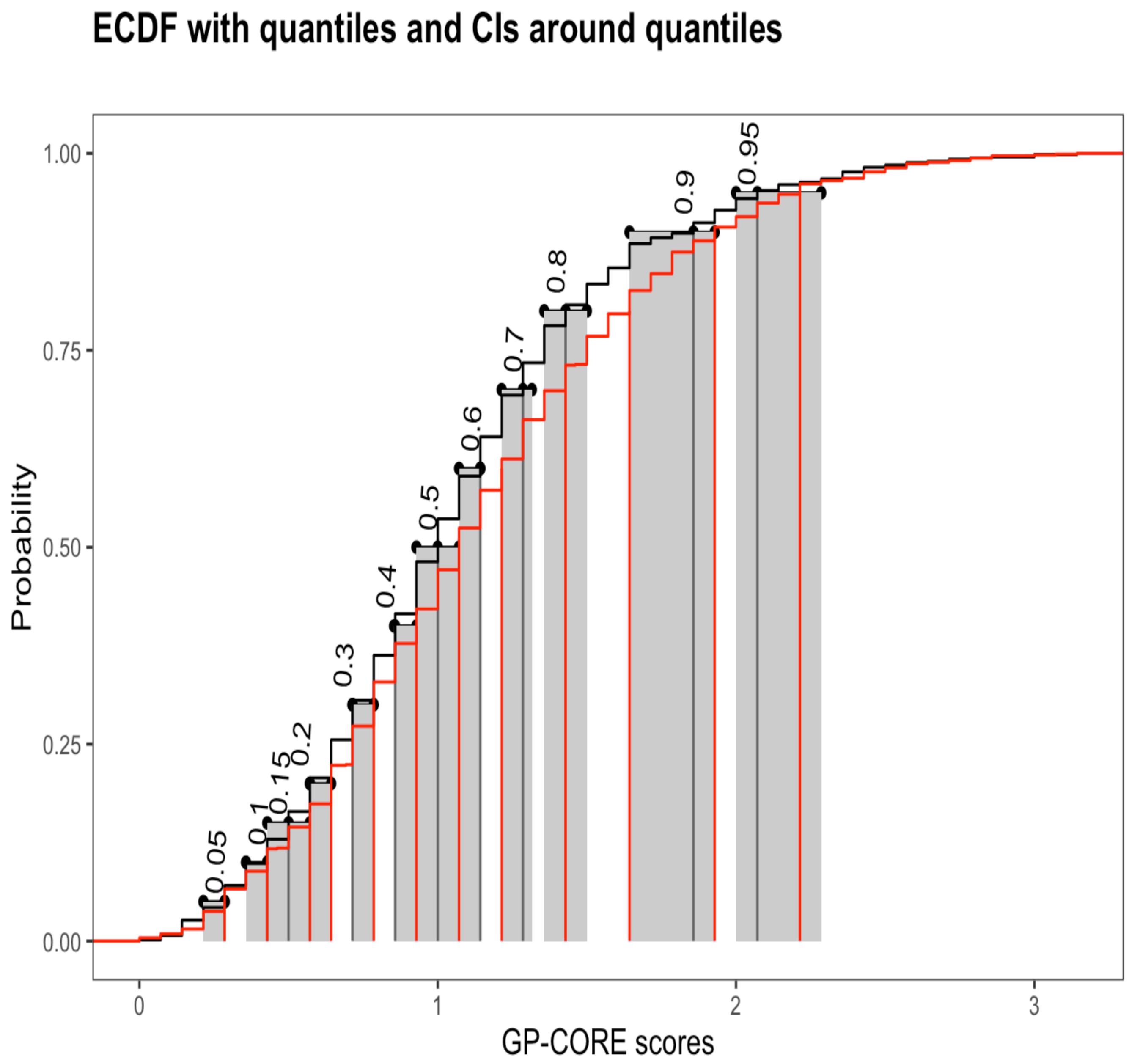

CORE-10

Table of the distribution by gender

prob	n	nOK	nMiss	quantile	LCL	UCL	form	gender
0.05	681	681	0	0.100	0.100000	0.1	CORE-10	men
0.10	681	681	0	0.200	0.100000	0.2	CORE-10	men
0.15	681	681	0	0.300	0.200000	0.3	CORE-10	men
0.20	681	681	0	0.300	0.300000	0.4	CORE-10	men
0.30	681	681	0	0.400	0.400000	0.5	CORE-10	men

prob	n	nOK	nMiss	quantile	LCL	UCL	form	gender
0.40	681	681	0	0.500	0.500000	0.6	CORE-10	men
0.50	681	681	0	0.700	0.600000	0.7	CORE-10	men
0.60	681	681	0	0.800	0.700000	0.8	CORE-10	men
0.70	681	681	0	1.000	0.900000	1.0	CORE-10	men
0.80	681	681	0	1.100	1.100000	1.2	CORE-10	men
0.90	681	681	0	1.500	1.371864	1.6	CORE-10	men
0.95	681	681	0	1.900	1.700000	2.0	CORE-10	men
0.05	982	982	0	0.100	0.100000	0.2	CORE-10	women
0.10	982	982	0	0.200	0.200000	0.3	CORE-10	women
0.15	982	982	0	0.300	0.300000	0.3	CORE-10	women
0.20	982	982	0	0.400	0.300000	0.4	CORE-10	women
0.30	982	982	0	0.500	0.500000	0.5	CORE-10	women
0.40	982	982	0	0.600	0.600000	0.7	CORE-10	women
0.50	982	982	0	0.800	0.700000	0.8	CORE-10	women
0.60	982	982	0	0.900	0.900000	1.0	CORE-10	women
0.70	982	982	0	1.100	1.100000	1.2	CORE-10	women
0.80	982	982	0	1.300	1.300000	1.4	CORE-10	women
0.90	982	982	0	1.700	1.600000	1.8	CORE-10	women
0.95	982	982	0	1.955	1.800000	2.1	CORE-10	women

Plot of the distributions by gender (black = men, red = women), quantiles= .05, .1, .15, .2, .3, .4, .5, .6, .7, .8, .9, .95

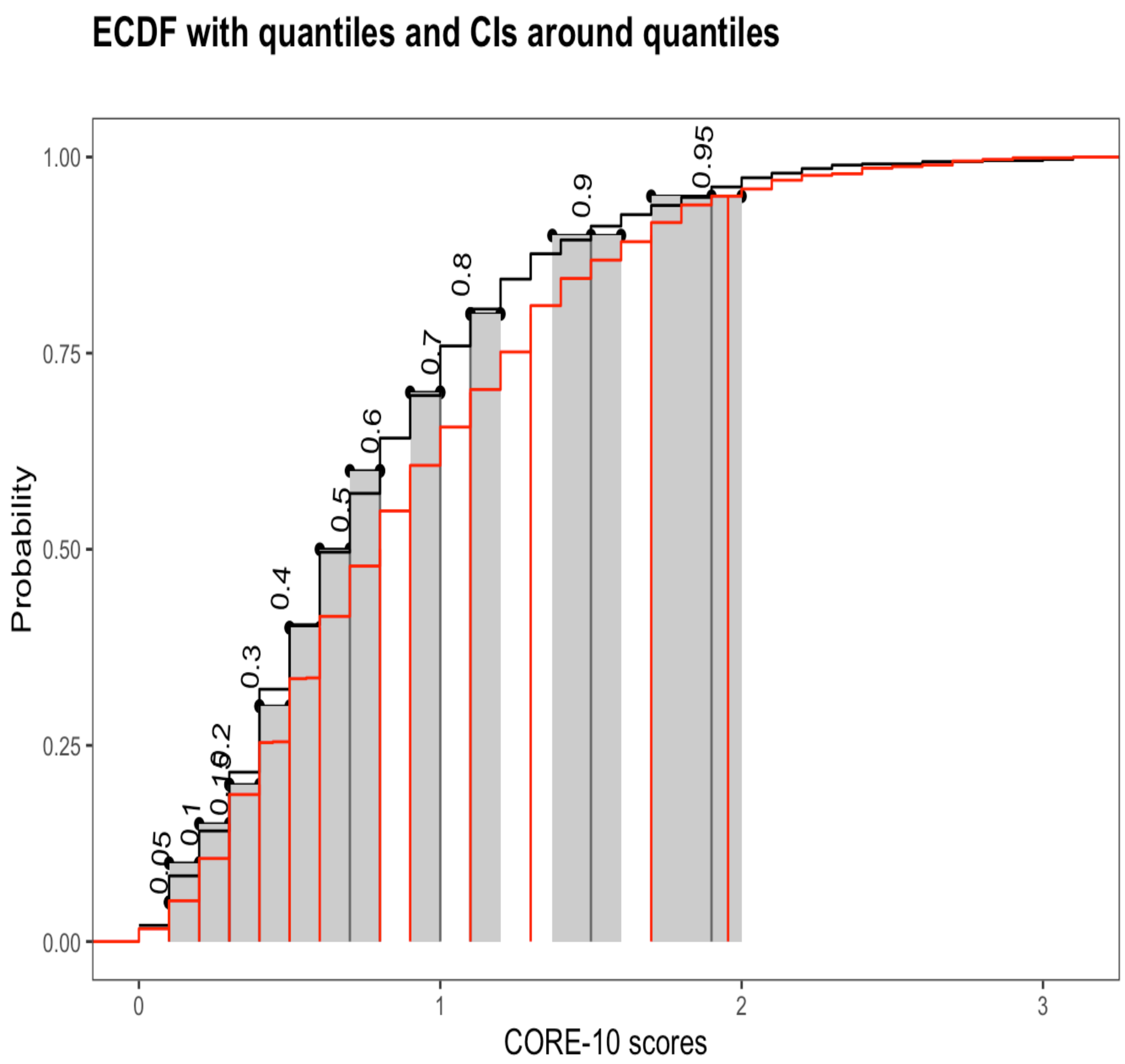

BY Education

CORE-OM

Table of the distribution by level of education

prob	n	nOK	nMiss	quantile	LCL	UCL	form	education
0.05	877	877	0	0.2500000	0.2142857	0.2857143	CORE-OM	higher education

prob	n	nOK	nMiss	quantile	LCL	UCL	form	education
0.10	877	877	0	0.3214286	0.2857143	0.3571429	CORE-OM	higher education
0.15	877	877	0	0.3928571	0.3571429	0.4642857	CORE-OM	higher education
0.20	877	877	0	0.4642857	0.4642857	0.5000000	CORE-OM	higher education
0.30	877	877	0	0.6071429	0.5714286	0.6428571	CORE-OM	higher education
0.40	877	877	0	0.7500000	0.7142857	0.7857143	CORE-OM	higher education
0.50	877	877	0	0.8571429	0.8214286	0.8928571	CORE-OM	higher education
0.60	877	877	0	1.0000000	0.9285714	1.0357143	CORE-OM	higher education
0.70	877	877	0	1.1785714	1.1071429	1.2500000	CORE-OM	higher education
0.80	877	877	0	1.3571429	1.3214286	1.4285714	CORE-OM	higher education
0.90	877	877	0	1.6785714	1.6071429	1.7500000	CORE-OM	higher education
0.95	877	877	0	2.0000000	1.8928571	2.0714286	CORE-OM	higher education
0.05	774	774	0	0.2142857	0.1715219	0.2857143	CORE-OM-NR	12 or less
0.10	774	774	0	0.3571429	0.3214286	0.3784118	CORE-OM-NR	12 or less
0.15	774	774	0	0.4642857	0.3928571	0.4642857	CORE-OM-NR	12 or less
0.20	774	774	0	0.5000000	0.4642857	0.5714286	CORE-OM-NR	12 or less
0.30	774	774	0	0.6785714	0.6428571	0.7500000	CORE-OM-NR	12 or less
0.40	774	774	0	0.8214286	0.7857143	0.8571429	CORE-OM-NR	12 or less
0.50	774	774	0	0.9821429	0.9285714	1.0357143	CORE-OM-NR	12 or less
0.60	774	774	0	1.1428571	1.0752562	1.2142857	CORE-OM-NR	12 or less
0.70	774	774	0	1.3214286	1.2500000	1.3928571	CORE-OM-NR	12 or less
0.80	774	774	0	1.5714286	1.5000000	1.6428571	CORE-OM-NR	12 or less
0.90	774	774	0	1.9285714	1.8358739	2.0714286	CORE-OM-NR	12 or less

prob	n	nOK	nMiss	quantile	LCL	UCL	form	education
0.95	774	774	0	2.2142857	2.1071429	2.3571429	CORE-OM-NR	12 or less

Plot of the distributions by level of education (black = higher education, red = less than 12 years of education), quantiles= .05, .1, .15, .2, .3, .4, .5, .6, .7, .8, .9, .95

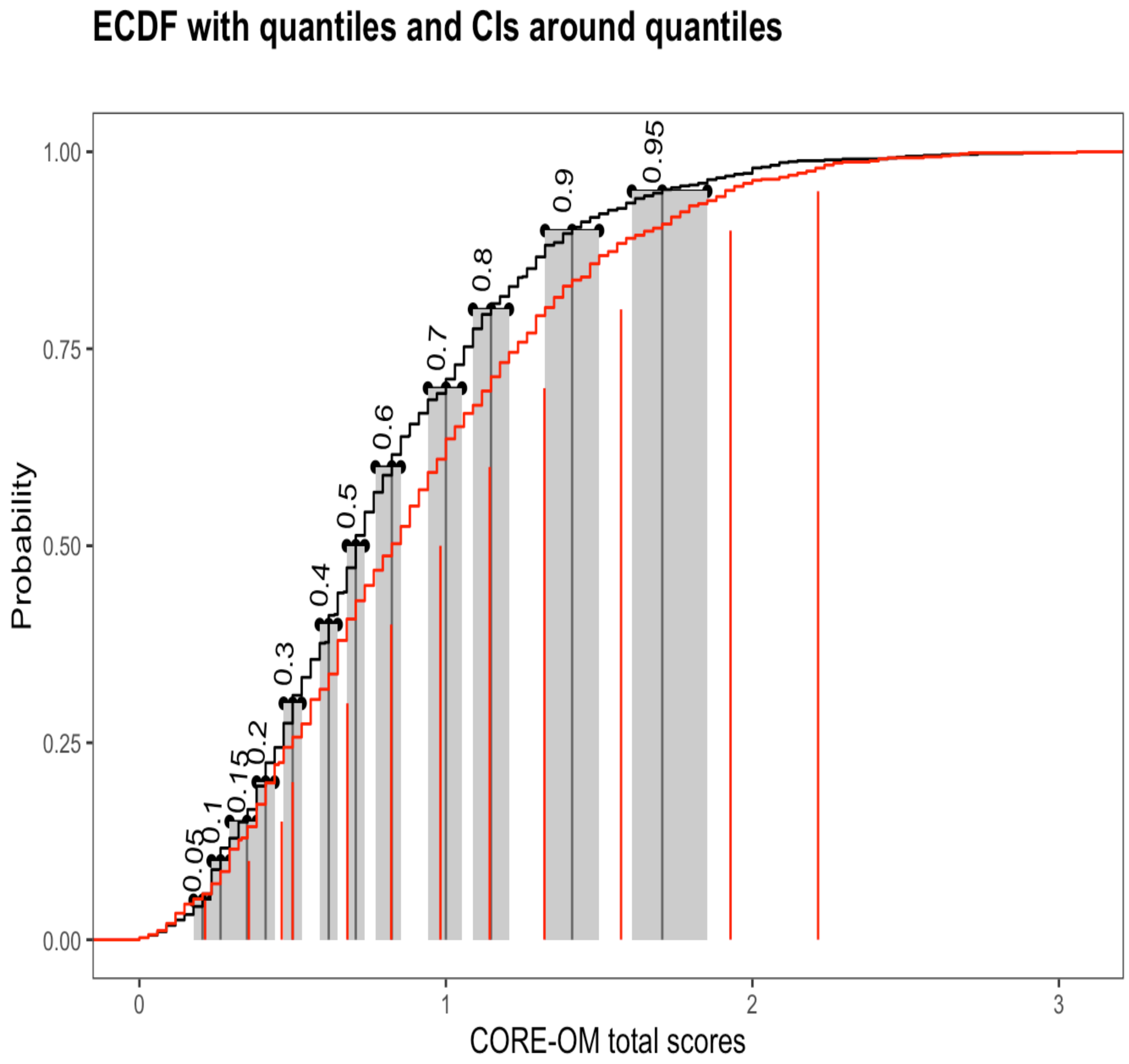

CORE-OM-NR

Table of the distribution by level of education

prob	n	nOK	nMiss	quantile	LCL	UCL	form	education
0.05	877	877	0	0.2500000	0.2142857	0.2857143	CORE-OM-NR	higher education
0.10	877	877	0	0.3214286	0.2857143	0.3571429	CORE-OM-NR	higher education
0.15	877	877	0	0.3928571	0.3571429	0.4642857	CORE-OM-NR	higher education
0.20	877	877	0	0.4642857	0.4642857	0.5000000	CORE-OM-NR	higher education
0.30	877	877	0	0.6071429	0.5714286	0.6428571	CORE-OM-NR	higher education
0.40	877	877	0	0.7500000	0.7142857	0.7857143	CORE-OM-NR	higher education
0.50	877	877	0	0.8571429	0.8214286	0.8928571	CORE-OM-NR	higher education
0.60	877	877	0	1.0000000	0.9285714	1.0357143	CORE-OM-NR	higher education
0.70	877	877	0	1.1785714	1.1071429	1.2500000	CORE-OM-NR	higher education
0.80	877	877	0	1.3571429	1.3214286	1.4285714	CORE-OM-NR	higher education
0.90	877	877	0	1.6785714	1.6071429	1.7500000	CORE-OM-NR	higher education
0.95	877	877	0	2.0000000	1.8928571	2.0714286	CORE-OM-NR	higher education
0.05	774	774	0	0.2142857	0.1715219	0.2857143	CORE-OM-NR	higher education
0.10	774	774	0	0.3571429	0.3214286	0.3784118	CORE-OM-NR	higher education
0.15	774	774	0	0.4642857	0.3928571	0.4642857	CORE-OM-NR	higher education
0.20	774	774	0	0.5000000	0.4642857	0.5714286	CORE-OM-NR	higher education
0.30	774	774	0	0.6785714	0.6428571	0.7500000	CORE-OM-NR	higher education
0.40	774	774	0	0.8214286	0.7857143	0.8571429	CORE-OM-NR	higher education
0.50	774	774	0	0.9821429	0.9285714	1.0357143	CORE-OM-NR	higher education
0.60	774	774	0	1.1428571	1.0752562	1.2142857	CORE-OM-NR	higher education
0.70	774	774	0	1.3214286	1.2500000	1.3928571	CORE-OM-NR	higher education
0.80	774	774	0	1.5714286	1.5000000	1.6428571	CORE-OM-NR	higher education

prob	n	nOK	nMiss	quantile	LCL	UCL	form	education
0.90	774	774	0	1.9285714	1.8358739	2.0714286	CORE-OM-NR	higher education
0.95	774	774	0	2.2142857	2.1071429	2.3571429	CORE-OM-NR	higher education

Plot of the distributions by level of education (black = higher education, red = less than 12 years), quantiles= .05, .1, .15, .2, .3, .4, .5, .6, .7, .8, .9, .95

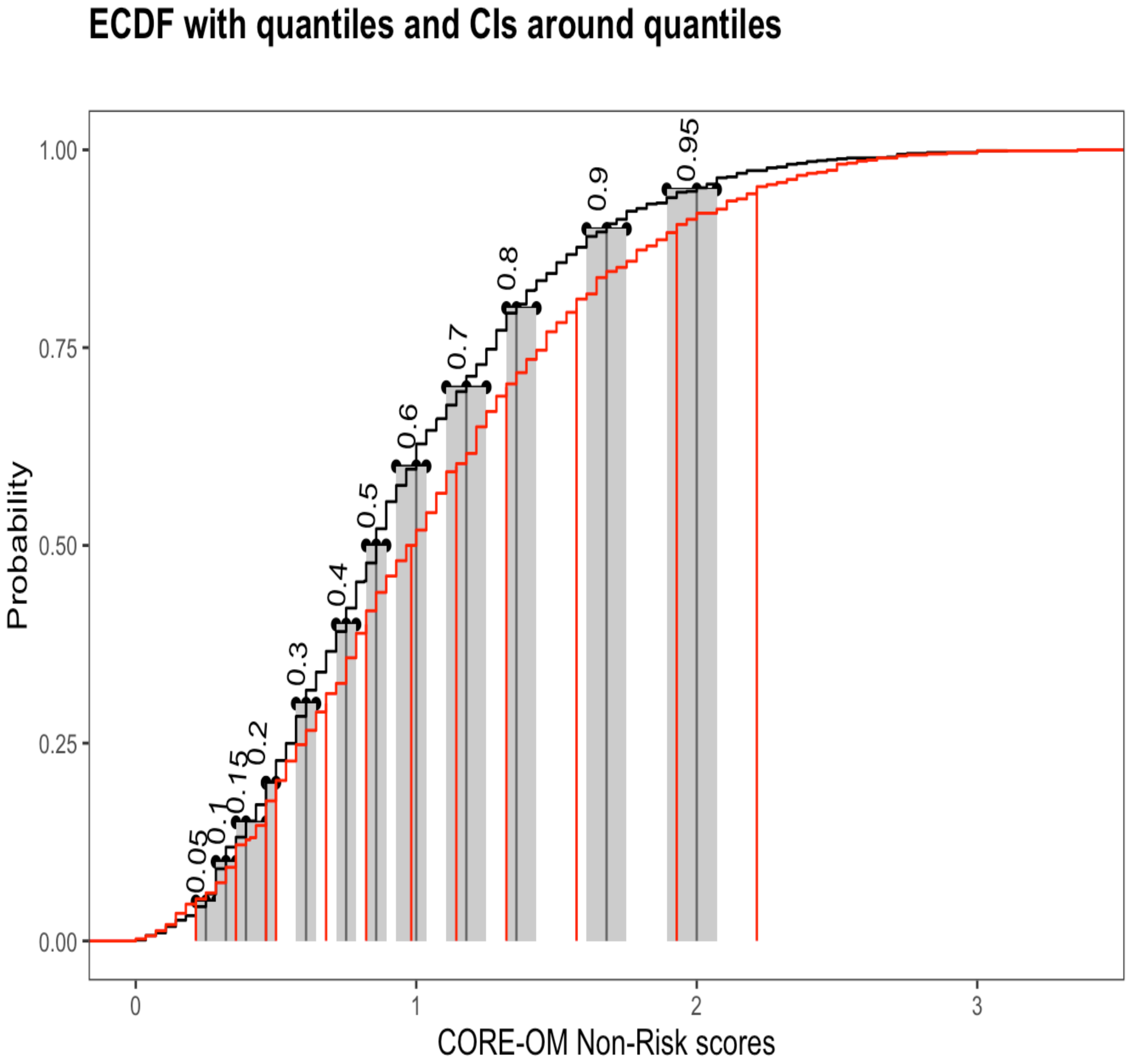

CORE-SFA

Table of the distribution by level of education

prob	n	nOK	nMiss	quantile	LCL	UCL	form	education
0.05	877	877	0	0.1666667	0.1111111	0.2222222	CORE-SFA	higher education
0.10	877	877	0	0.2777778	0.2777778	0.3333333	CORE-SFA	higher education
0.15	877	877	0	0.3333333	0.3333333	0.3888889	CORE-SFA	higher education
0.20	877	877	0	0.4444444	0.3888889	0.4444444	CORE-SFA	higher education
0.30	877	877	0	0.5555556	0.5000000	0.5555556	CORE-SFA	higher education
0.40	877	877	0	0.6666667	0.6111111	0.6666667	CORE-SFA	higher education
0.50	877	877	0	0.7777778	0.7222222	0.8235294	CORE-SFA	higher education
0.60	877	877	0	0.8888889	0.8333333	0.9444444	CORE-SFA	higher education
0.70	877	877	0	1.0555556	1.0000000	1.1666667	CORE-SFA	higher education
0.80	877	877	0	1.2777778	1.2222222	1.3333333	CORE-SFA	higher education
0.90	877	877	0	1.6074074	1.5000000	1.6554948	CORE-SFA	higher education
0.95	877	877	0	1.8333333	1.7222222	1.9903470	CORE-SFA	higher education
0.05	774	774	0	0.1666667	0.1557007	0.2222222	CORE-SFA	12 or less
0.10	774	774	0	0.2777778	0.2222222	0.3333333	CORE-SFA	12 or less
0.15	774	774	0	0.3888889	0.3333333	0.4092547	CORE-SFA	12 or less
0.20	774	774	0	0.4444444	0.3888889	0.5000000	CORE-SFA	12 or less
0.30	774	774	0	0.6111111	0.5555556	0.6666667	CORE-SFA	12 or less
0.40	774	774	0	0.7222222	0.6666667	0.7777778	CORE-SFA	12 or less
0.50	774	774	0	0.8888889	0.8333333	0.9444444	CORE-SFA	12 or less
0.60	774	774	0	1.0000000	1.0000000	1.0555556	CORE-SFA	12 or less
0.70	774	774	0	1.2222222	1.1666667	1.2777778	CORE-SFA	12 or less
0.80	774	774	0	1.4444444	1.3888889	1.5555556	CORE-SFA	12 or less
0.90	774	774	0	1.7777778	1.6666667	1.8888889	CORE-SFA	12 or less
0.95	774	774	0	2.0555556	1.9444444	2.2222222	CORE-SFA	12 or less

Plot of the distributions by level of education (black = higher education, red = less than 12 years), quantiles= .05, .1, .15, .2, .3, .4, .5, .6, .7, .8, .9, .95

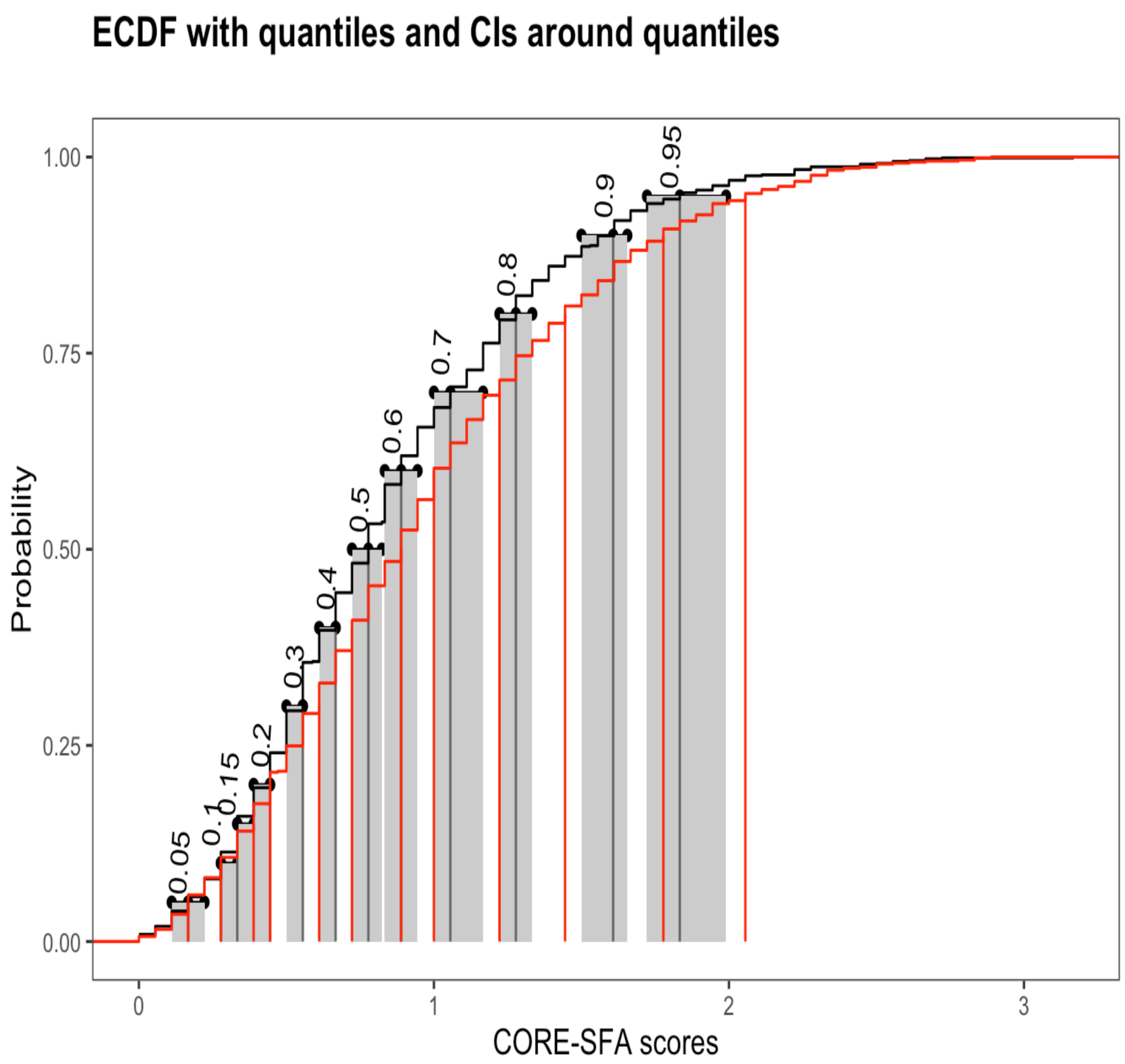

CORE-SFB

Table of the distribution by level of education

prob	n	nOK	nMiss	quantile	LCL	UCL	form	education
0.05	877	877	0	0.2222222	0.1666667	0.2222222	CORE-SFB	higher education
0.10	877	877	0	0.2777778	0.2777778	0.3333333	CORE-SFB	higher education
0.15	877	877	0	0.3888889	0.3333333	0.3937182	CORE-SFB	higher education

prob	n	nOK	nMiss	quantile	LCL	UCL	form	education
0.20	877	877	0	0.4444444	0.3888889	0.5000000	CORE-SFB	higher education
0.30	877	877	0	0.5555556	0.5555556	0.6111111	CORE-SFB	higher education
0.40	877	877	0	0.7222222	0.6666667	0.7777778	CORE-SFB	higher education
0.50	877	877	0	0.8333333	0.7777778	0.8888889	CORE-SFB	higher education
0.60	877	877	0	0.9444444	0.8888889	1.0000000	CORE-SFB	higher education
0.70	877	877	0	1.1111111	1.0555556	1.1666667	CORE-SFB	higher education
0.80	877	877	0	1.2777778	1.2777778	1.3888889	CORE-SFB	higher education
0.90	877	877	0	1.6111111	1.5000000	1.7222222	CORE-SFB	higher education
0.95	877	877	0	1.8888889	1.7777778	2.0000000	CORE-SFB	higher education
0.05	774	774	0	0.1666667	0.1557007	0.2222222	CORE-SFB	12 or less
0.10	774	774	0	0.3241830	0.2777778	0.3333333	CORE-SFB	12 or less
0.15	774	774	0	0.4157407	0.3888889	0.4444444	CORE-SFB	12 or less
0.20	774	774	0	0.5000000	0.4444444	0.5555556	CORE-SFB	12 or less
0.30	774	774	0	0.6666667	0.6111111	0.7222222	CORE-SFB	12 or less
0.40	774	774	0	0.7777778	0.7504786	0.8333333	CORE-SFB	12 or less
0.50	774	774	0	0.9444444	0.8888889	1.0000000	CORE-SFB	12 or less
0.60	774	774	0	1.1111111	1.0555556	1.1666667	CORE-SFB	12 or less
0.70	774	774	0	1.2777778	1.2222222	1.3333333	CORE-SFB	12 or less
0.80	774	774	0	1.5000000	1.4444444	1.6111111	CORE-SFB	12 or less
0.90	774	774	0	1.8333333	1.7777778	1.9444444	CORE-SFB	12 or less
0.95	774	774	0	2.1111111	2.0000000	2.2777778	CORE-SFB	12 or less

Plot of the distributions by level of education (black = higher education, red = less than 12 years), quantiles= .05, .1, .15, .2, .3, .4, .5, .6, .7, .8, .9, .95

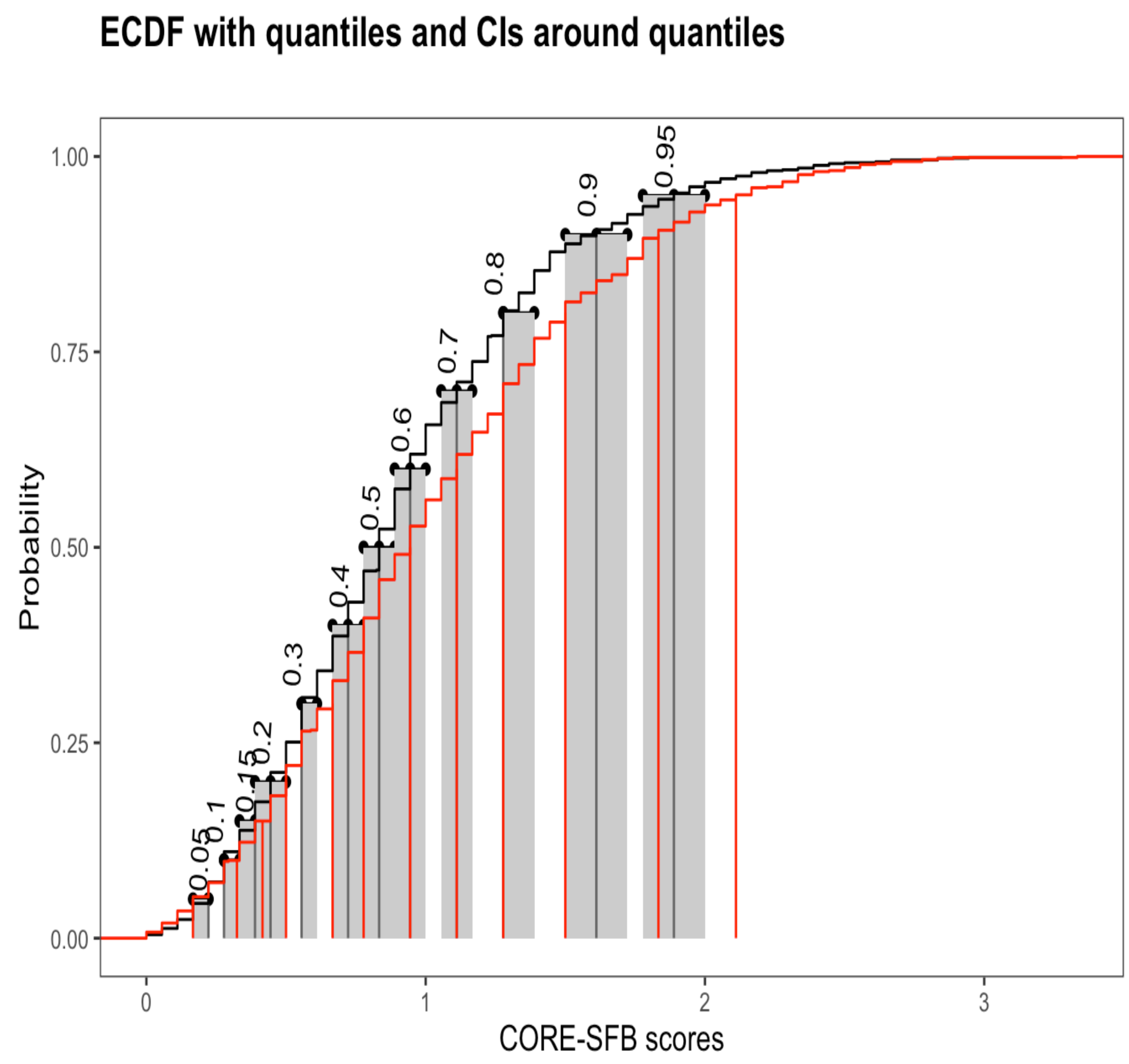

GP-CORE

Table of the distribution by level of education

prob	n	nOK	nMiss	quantile	LCL	UCL	form	education
0.05	877	877	0	0.2857143	0.2142857	0.2857143	GP-CORE	higher education
0.10	877	877	0	0.4285714	0.3571429	0.4285714	GP-CORE	higher education
0.15	877	877	0	0.5000000	0.4285714	0.5714286	GP-CORE	higher education

prob	n	nOK	nMiss	quantile	LCL	UCL	form	education
0.20	877	877	0	0.6428571	0.5714286	0.6428571	GP-CORE	higher education
0.30	877	877	0	0.7142857	0.7142857	0.7857143	GP-CORE	higher education
0.40	877	877	0	0.8571429	0.7857143	0.9285714	GP-CORE	higher education
0.50	877	877	0	1.0000000	0.9285714	1.0714286	GP-CORE	higher education
0.60	877	877	0	1.1428571	1.0714286	1.1428571	GP-CORE	higher education
0.70	877	877	0	1.2857143	1.2142857	1.3419963	GP-CORE	higher education
0.80	877	877	0	1.4285714	1.4285714	1.5000000	GP-CORE	higher education
0.90	877	877	0	1.7142857	1.6428571	1.8571429	GP-CORE	higher education
0.95	877	877	0	2.0714286	1.9285714	2.1428571	GP-CORE	higher education
0.05	774	774	0	0.2857143	0.2142857	0.3571429	GP-CORE	12 or less
0.10	774	774	0	0.4285714	0.3571429	0.5000000	GP-CORE	12 or less
0.15	774	774	0	0.5714286	0.5000000	0.5714286	GP-CORE	12 or less
0.20	774	774	0	0.6428571	0.5714286	0.7142857	GP-CORE	12 or less
0.30	774	774	0	0.7857143	0.7798474	0.8571429	GP-CORE	12 or less
0.40	774	774	0	0.9285714	0.9285714	1.0000000	GP-CORE	12 or less
0.50	774	774	0	1.0714286	1.0714286	1.1428571	GP-CORE	12 or less
0.60	774	774	0	1.2142857	1.2142857	1.2857143	GP-CORE	12 or less
0.70	774	774	0	1.4285714	1.3571429	1.5000000	GP-CORE	12 or less
0.80	774	774	0	1.6428571	1.5714286	1.7857143	GP-CORE	12 or less
0.90	774	774	0	2.0000000	1.9285714	2.0714286	GP-CORE	12 or less
0.95	774	774	0	2.2142857	2.1428571	2.4285714	GP-CORE	12 or less

Plot of the distributions by level of education (black = higher education, red = less than 12 years), quantiles= .05, .1, .15, .2, .3, .4, .5, .6, .7, .8, .9, .95

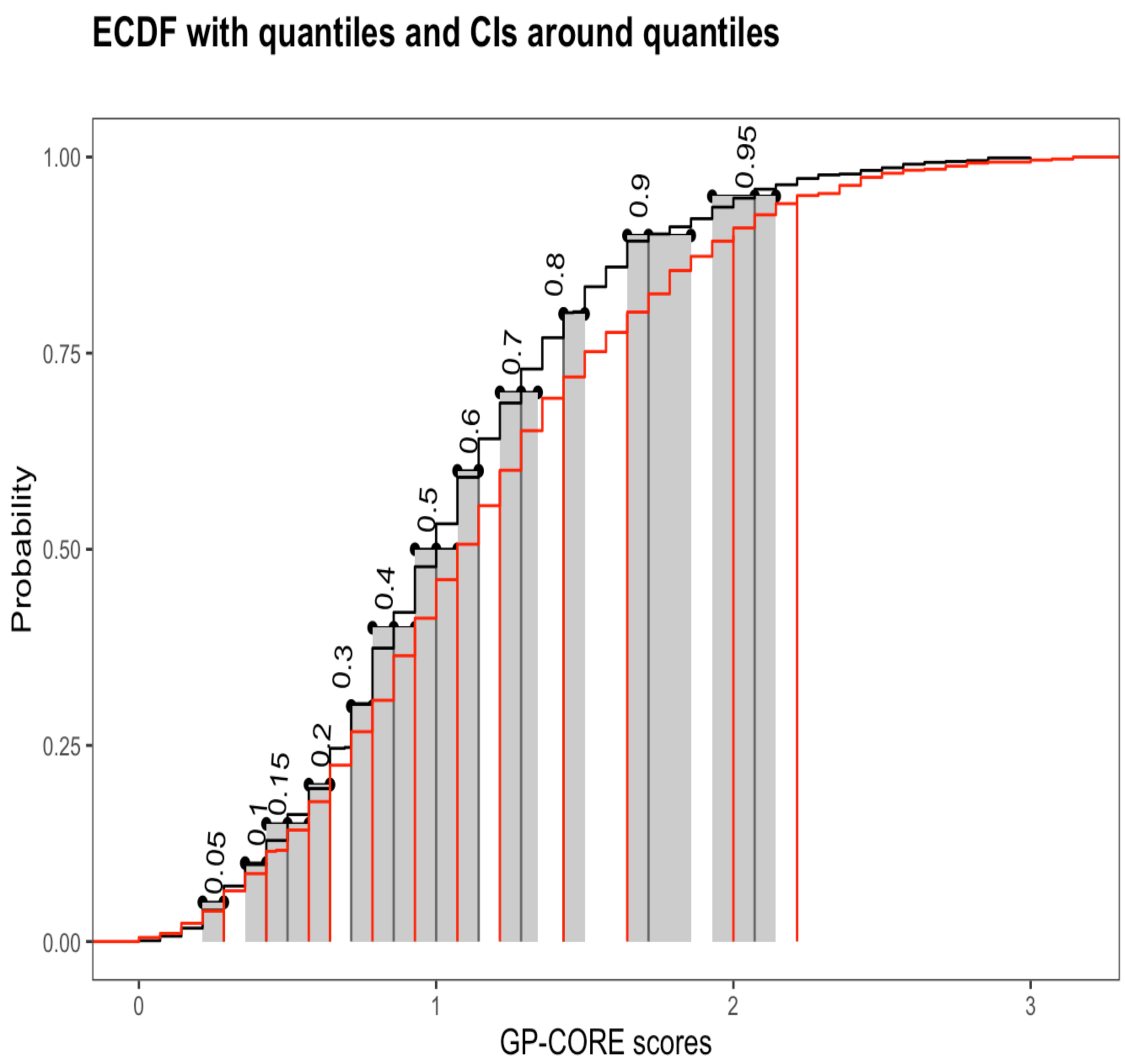

CORE-10

Table of the distribution by level of education

prob	n	nOK	nMiss	quantile	LCL	UCL	form	education
0.05	877	877	0	0.1	0.1	0.100000	CORE-10	higher education
0.10	877	877	0	0.2	0.2	0.200000	CORE-10	higher education
0.15	877	877	0	0.3	0.2	0.300000	CORE-10	higher education
0.20	877	877	0	0.3	0.3	0.400000	CORE-10	higher education
0.30	877	877	0	0.4	0.4	0.500000	CORE-10	higher education

prob	n	nOK	nMiss	quantile	LCL	UCL	form	education
0.40	877	877	0	0.6	0.5	0.600000	CORE-10	higher education
0.50	877	877	0	0.7	0.6	0.700000	CORE-10	higher education
0.60	877	877	0	0.8	0.8	0.900000	CORE-10	higher education
0.70	877	877	0	1.0	0.9	1.000000	CORE-10	higher education
0.80	877	877	0	1.2	1.1	1.300000	CORE-10	higher education
0.90	877	877	0	1.5	1.4	1.600000	CORE-10	higher education
0.95	877	877	0	1.8	1.7	1.982625	CORE-10	higher education
0.05	774	774	0	0.1	0.1	0.200000	CORE-10	12 or less
0.10	774	774	0	0.2	0.2	0.300000	CORE-10	12 or less
0.15	774	774	0	0.3	0.3	0.300000	CORE-10	12 or less
0.20	774	774	0	0.4	0.3	0.400000	CORE-10	12 or less
0.30	774	774	0	0.5	0.5	0.500000	CORE-10	12 or less
0.40	774	774	0	0.6	0.6	0.700000	CORE-10	12 or less
0.50	774	774	0	0.8	0.7	0.800000	CORE-10	12 or less
0.60	774	774	0	1.0	0.9	1.000000	CORE-10	12 or less
0.70	774	774	0	1.1	1.1	1.200000	CORE-10	12 or less
0.80	774	774	0	1.3	1.3	1.400000	CORE-10	12 or less
0.90	774	774	0	1.7	1.6	1.800000	CORE-10	12 or less
0.95	774	774	0	2.0	1.9	2.100000	CORE-10	12 or less

Plot of the distributions by level of education (black = higher education, red = less than 12 years), quantiles= .05, .1, .15, .2, .3, .4, .5, .6, .7, .8, .9, .95

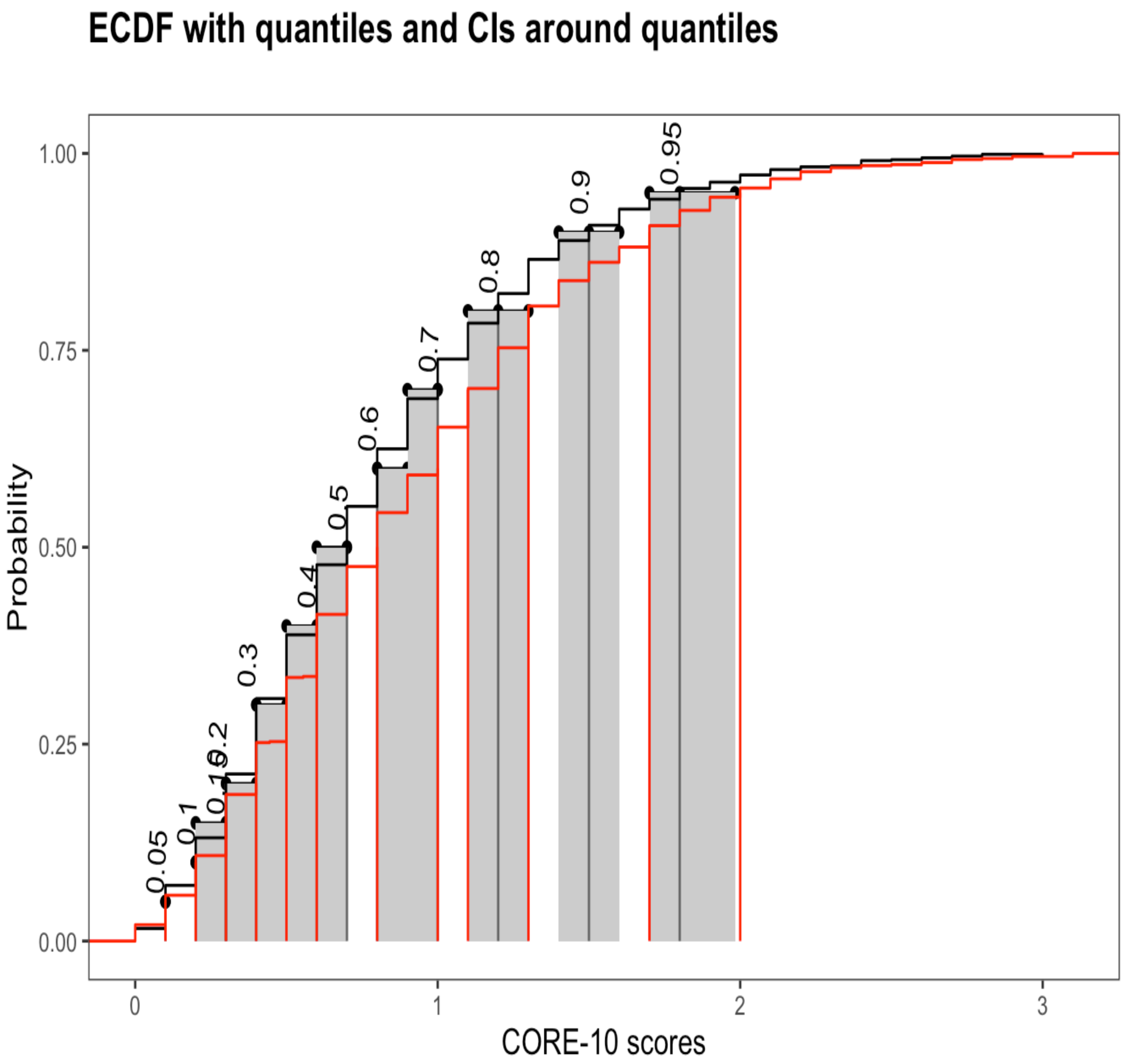
ECDF with quantiles and CIs around quantiles
Probability
CORE-10 scores
1.00
0.75
0.50
0.25
0.00
0
1
2
3
0.05
0.1
0.15
0.2
0.3
0.4
0.5
0.6
0.7
0.8
0.9
0.95
